# Chemical analysis of *Brasilimeria* Stach, 1949 (Hexapoda, Collembola, Neanuridae) hemolymphatic secretion, and description of a new species

**DOI:** 10.1371/journal.pone.0212451

**Published:** 2019-02-21

**Authors:** Douglas Zeppelini, Gabriel C. Queiroz, Norberto P. Lopes, Francisco J. B. Mendonça-Junior

**Affiliations:** 1 Laboratório de Sistemática de Colembolla e Conservação, Department of Biological Sciences, State University of Paraíba, João Pessoa—PB, Brazil; 2 Setor de Apterygota, Department of Entomology, Nacional Museum/Federal University of Rio de Janeiro, Rio de Janeiro––RJ, Brasil; 3 Núcleo de Pesquisa em Produtos Naturais e Sintéticos, Department of Physics and Chemistry, School of Pharmaceutical Sciences of Ribeirão Preto, University of São Paulo, Ribeirão Preto—SP, Brazil; 4 Laboratório de Síntese e Vetorização de Moléculas, Department of Biological Sciences, State University of Paraíba, João Pessoa—PB, Brazil; Montana State University Bozeman, UNITED STATES

## Abstract

Though Collembola is a widespread hexapod its use of chemical compounds for defense has been reported for only a few European species. Chemical composition analyses of the hemolymphatic secretion of Neotropical Collembola using Gas Chromatography with Mass Spectrometry (GC-MS) has been performed for the first time. The GC-MS analysis revealed 32 constituents, such as aliphatic and aromatic hydrocarbons, esters, alcohols, a phenol, an aldehyde and a ketone. Benzyl benzoate, the main component (at 46.98%), is a compound with known acaricide and insecticide properties. This is the first report on chemical constituents produced by Neotropical Pseudachorutinae, genus *Brasilimeria*, and will permit future secretion comparisons for Collembola. The taxonomic description of the species producing the secretion analyzed is provided; *Brasilimeria assu* sp. nov. (Collembola, Neanuridae, Pseudachorutinae) is the third known species of the genus; an updated diagnosis of the genus, an identification key, and further remarks on the species *Brasilimeria* Stach, 1949 are provided.

## Introduction

Chemical defense is widespread among terrestrial arthropods but it is especially diverse within Hexapoda [1, 2]. In Hexapoda, much attention has been given to insects, due to their extraordinary diversity, ecological, and economic importance. Collembola are basal hexapods, they represent one of the first Pancrustacea groups to conquer land [3]. Collembola have also been studied regarding excretion of chemical deterrent compounds [4–7] which are usually related to the soil and leaf litter profiles of their dwelling locations; and to their feedings on fungi, decaying litter, and organic debris. All of these play important roles in soil production and fungi dispersion.

Collembola is subdivided into four major groups: Entomobryomorpha, Poduromorpha, Symphypleona, and Neelipleona, which are strikingly different in morphology and body tagmosis [8]. Messner et al. [7] compiled species of Collembola which had already been reported as unpalatable or toxic to their predators. Most of such deterrent Collembola species reported belong to Poduromorpha, the most conserved Collembola. According to Messner et al. [7], to avoid predation it is possible that all Collembola poduromorphs present at least some type of deterring substance. However, studies so far have been limited to five of the 11 poduromorph families (Table 1): Onychiuridae, Tullbergiidae, Hypogastruridae, Poduridae and Neanuridae. Fluids, secretions and other hemolymphatic substances have not until now been chemically analyzed for Neotropical Collembola.

**Table 1:** Poduromorpha species reported as toxic or unpalatable to arthropod predators. Modified and taxonomically updated from Messner et al. [7].

**Onychiuridae**
*Onychiurus fimetarius* Denis, 1938
*Protaphorura* cf. *armata* (Tullberg, 1869)
*Protaphorura fimata* (Gisin, 1952)
*Tetrodontophora bielanensis* (Waga, 1842)
**Tullbergiidae**
*Mesaphorura krausbaueri* Börner, 1901
**Hypogastruridae**
*Ceratophysella sigillata* (Uzel, 1891)
*C*. *bengtssoni* (Agren, 1904)
*Hypogastrura denticulata* (Bagnall, 1941)
*H*. *viatica* (Tullberg, 1872)
*Mesachorutes quadriocellatus* Absolon, 1900
*Schoettella ununguiculata* (Tullberg, 1869)
*Xenylla humicola* (Fabricius, 1780)
*X*. *grisea* (Axelson, 1900)
**Neanuridae**
**Pseudachorutinae**
*Anurida maritima (Guérin-Méneville*, *1836)*
**Neanurinae**
*Neanura muscorum (Templeton*, *1836)*
**Poduridae**
*Podura aquatica Linnaeus*, *1758*

Belonging in Onychiuroidae, the families Onychiuridae and Tullbergiidae are characterized by the presence of special cuticular structures called pseudocelli, which secrete repellant substances [9]. The Neanuridae family subdivided into six subfamilies: Caputanurininae, Morulininae, Uchidanurinae, Frieseinae, Neanurinae *sensu* Cassagnau [10] and Pseudachorutinae, these last two are the most diversified [8, 11]. The Neotropical Poduromorpha fauna can be largely characterized by Pseudachorutinae, which presents many endemic taxa, including the genus *Brasilimeria* Stach, 1949 [12]; defined by Stach [13] and characterized by the absence of PAO and furca, the presence of 6+6 eyes disposed in a semi-circle, and large sized adult specimens. Today it includes only two species, *Brasilimeria anura* (Arlé, 1939) and *Brasilimeria wygodzinskyi* (Arlé, 1943), which have been described in Southeastern Brazil. The species herein described; *Brasilimeria assu* sp. nov., was found in the same Brazilian region, a well-known biodiversity hotspot of the Brazilian Atlantic Tropical Forest.

Mass spectrometry (MS) is one of the most popular analytical techniques for chemical composition identification [14, 15]. Currently, it is applied to identify "small molecules", (compounds with less than 500Da of weight), isolated from plants [16], fungi [17], algae, [18], animal gland secretions [19], bacteria and microorganisms [20]. Its use also includes diagnostic tests in biological fluids [21], doping tests [22], identification of pesticides or contaminants in the environment [23], and food and beverage quality control [24].

In this context, since there are so few studies identifying and quantifying chemical metabolites in Neotropical Collembola secretions, GC-MS chemical/analytical techniques were considered appropriate to analyze *Brasilimeria assu* sp. nov., (Collembola, Neanuridae, Pseudachorutinae). A taxonomic description of the new species and an identification key for species of the genus are also provided.

## Materials and methods

### Sampling

**Fig 1:**
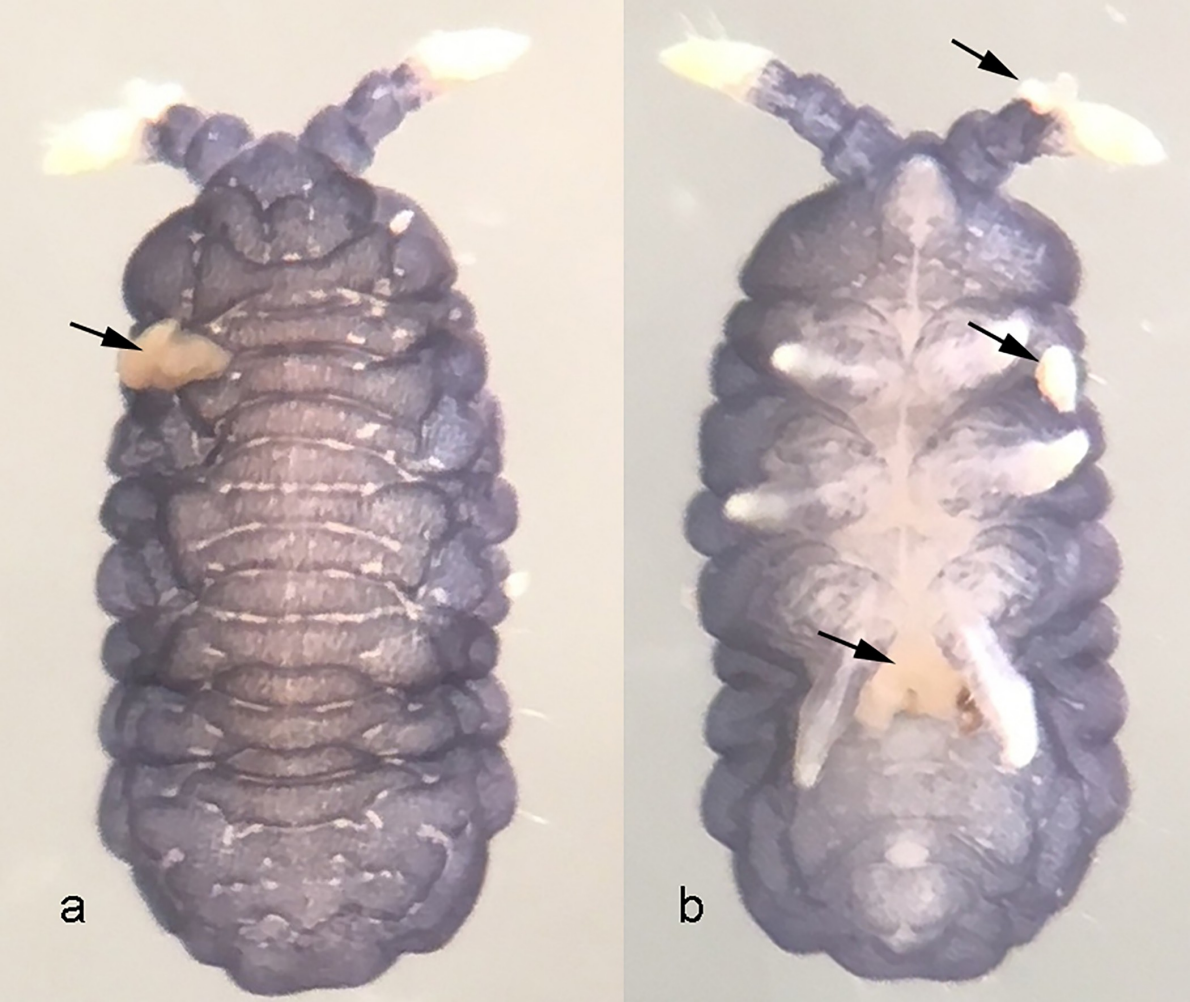
*Brasilimeria anura* (Arlé, 1939). (A) Dorsal view. (B) Ventral view. Arrows indicate reflex bleeding sites and hemolymphatic secretion droplets.

Specimens were retrieved from the environment passively; a soil and litter sample was taken from the site, processed in a Berlese funnel for seven days, and the specimens were fixed in ethanol 92°GL. Hemolymphatic droplets were released through weak spots in the cuticle, also known as reflex bleeding sites (Fig 1).

Sampling site: *Serra dos Órgãos* National Park, Teresópolis municipality, Rio de Janeiro, Brazil. The biotope is soil with a litter of high altitude vegetation on rocky Southeastern Brazil mountains; also known as the Brazilian *Páramos*. Queiroz, G.C. leg. 14.iii. 2012. Altitude: 2129 m above sea level. Local coordinates: 22°27'31.21"S 43°1'43.21"W. Collections were performed under license MMA-ICMBio-SISBIO number 25054–3.

The samples (droplets) of the *Brasilimeria assu* sp. nov., secretions were collected directly from the specimen with the aid of a capillary tube, and before being analyzed were kept in a glass vial under refrigeration.

### Nomenclatural Acts

The electronic edition of this article conforms to the requirements of the amended International Code of Zoological Nomenclature, and the new names contained within the electronic edition of this article are available using this same code. This published work and the nomenclature acts it contains have been registered with ZooBank, the online registration system for the ICZN. The ZooBank LSIDs (Life Science Identifiers) are available and the associated information can viewed through any standard web browser by appending the LSID to the prefix “http://zoobank.org/”. The LSID for this publication is: urn:lsid:zoobank.org:pub: 0D70962F-7F24-4EF3-8160-7B145F2C7555. The electronic edition of this work was published in a journal with an ISSN, and has been archived and is available from the following digital repositories: PubMed Central, LOCKSS.

#### Taxonomic Abbreviations

**Antennae**: AIIIO–antennal III organ; Ant.–antennal segment; ms–dorsolateral S-microchaeta; Sgd–dorsal guard S-chaeta of antennal III organ; Sgv–ventral guard S-chaeta of antennal III organ. **Head**: PAO–post-antennal organ. **Thorax and abdomen**: Abd.–abdominal segment; Di–dorsal internal group of chaetae; De–dorsal external group of chaetae; DL–dorsolateral group of chaetae; Th.–thoracic segment; tita–tibiotarsus; VT–ventral tube.

### GC-MS analysis

Chemical composition analysis of the secretions was performed using a Gas Chromatograph Mass Spectrometer (GC-MS), Shimadzu, model QP 2010. Separation of the chemical constituents was carried out using a DB-5MS column [(5%-phenyl)-methylpolysiloxane] brand Agilent J&W GC Columns, 30 m long, 0.25 mm internal diameter, thickness of the film 0.25 μm. The carrier gas was helium. The gas chromatograph operating conditions were: column internal pressure of 84 kPa, total flow 10 ml min^-1^, column flow of gas at 1.4 ml.min^-1^; Linear Velocity 42.7 cm.sec^-1^, Purge Flow: 3.0 mL.min^-1^, column ion source temperature 250°C, low vacuum 7.4 Pa, high vacuum 5.9^−4^ Pa, Injection Mode: Split-less, detector temperature at the (GC/MS) interface: 320°C. The initial column temperature was 40°C for 4 min, followed by increases of 8°C min^-1^ up to 320°C (being then kept constant for 59 min). The split ratio was 5:1. The mass spectrometry was programmed to perform readings in a range of 35 to 600 Da at intervals of 0.25 s, with the ionization EI (electrospray ionization) mode and ionization energy at 70 eV. One μl of secretion was injected (1 drop was dissolved in 2 ml of ethyl acetate). A mixture of linear hydrocarbons (C9-C20, C21-C40) was injected under the same conditions in order to help identify the chemical constituents. The identification of the constituents was based on data libraries; analyzing and comparing mass spectra (FFNSC1.3.lib, WILEY7.LIB, NIST08s.LIB, MY LIBRARY.lib). The GC/MS equipment indices presented similarities of ≥ 90%. Relative quantification of each constituent was obtained from the relative area of the peaks in the chromatogram.

## Results and discussion

### Taxonomy

           Collembola

                      Poduromorpha

                                 Neanuridae

                                            Pseudachorutinae

                                                       *Brasilimeria* Stach, 1949

**Genus Diagnosis** (modified and extended from Stach [13] and Massoud [25]).

Habitus similar to *Tijucameria* Mendonça & Fernandes, 2005. Dark blue to black coloration covering the entire body, or with yellow/white antennal segments and spots on body; the legs and ventral abdomen are paler. Body: stout, dorsoventrally flattened, well-developed paratergites and secondary granulation. Adults: always larger than 3mm. Antennae: smaller than head diagonal. Ant. III–IV dorsal chaetotaxy of *Handschinurida* type sensu Queiroz & Zeppelini [12]: presenting S1–S4, S8 and S10, ms absent, x chaetae between a1 and a3; trilobed apical bulb, subapical organite present; Ant. I with 10 chaetae or more. Eyes: 6+6, disposed in a semi-circle; PAO absent. Labial chaetae C and D: apically displaced. Elongate mandible, with two strong basal teeth and about 17 smaller apical teeth; maxilla styliform; juveniles present fewer mandibular teeth. Elongate legs; ratio Claw:Tita = 1:1.5, M chaetae basally displaced; claw with inner tooth and no lateral teeth. Frequent plurichaetosis and no heterochaetosis; S-chaetae formula by half tergite: 022/22211 or 022/11111; S-chaetae of body generally much longer than ordinary chaetae. Reduced ventral chaetotaxy, VT with 3+3 chaetae. Tenaculum and furca absent. Abd. VI not dorsally visible, being hidden under Abd. V.

#### Species Description

***Brasilimeria assu*** Queiroz & Zeppelini **sp. nov.** [urn:lsid:zoobank.org:act:71CA9B58-E2FE-4BC5-9D8B-C7CAB214167D] Holotype female, Brazil. *Serra dos Órgãos* National Park, Teresópolis municipality, Rio de Janeiro: litter and soil of Brazilian *Páramos* (high altitude) 2129m above sea level, 22°27'31.21"S 43°1'43.21"W, 2.Feb.2019, Queiroz, G.C. leg. Sample 82 Collembola Collection/MNRJ. Paratype: same data as Holotype.

Additional material: one male and one female. Brazil. *Petrópolis*, *Castelos do Açu*, RJ: litter and soil of Brazilian *Páramos* (high altitude), 2149m above sea level, 22°29'1.85"S 43° 3'36.67"W, 12.Oct.2013, Queiroz, G.C. leg. Sample 2349 CM/MNRJ. One juvenile, Brazil. *Serra dos Órgãos* National Park, Teresópolis municipality, Rio de Janeiro: litter and soil of Brazilian *Páramos* (high altitude) 2129m above sea level, 22°27'31.21"S 43°1'43.21"W, 14.Mar.2012, Queiroz, G.C. leg. Sample 2305 CM/MNRJ. Collection license MMA-ICMBio-SISBIO number 25054–3.

Body length: Holotype 4.9 mm; body length of paratype: 4.3 mm. General body color in ethanol: dark blue, with paler or almost white legs and ventral abdomen. Antennae smaller than cephalic diagonal. Buccal cone: short and forming a small beak. Secondary granules well developed. Well-developed paratergites, similar to *Tijucameria*.—Abd. VI; being hidden under Abd. V; is not dorsally visible.

**Fig 2:**
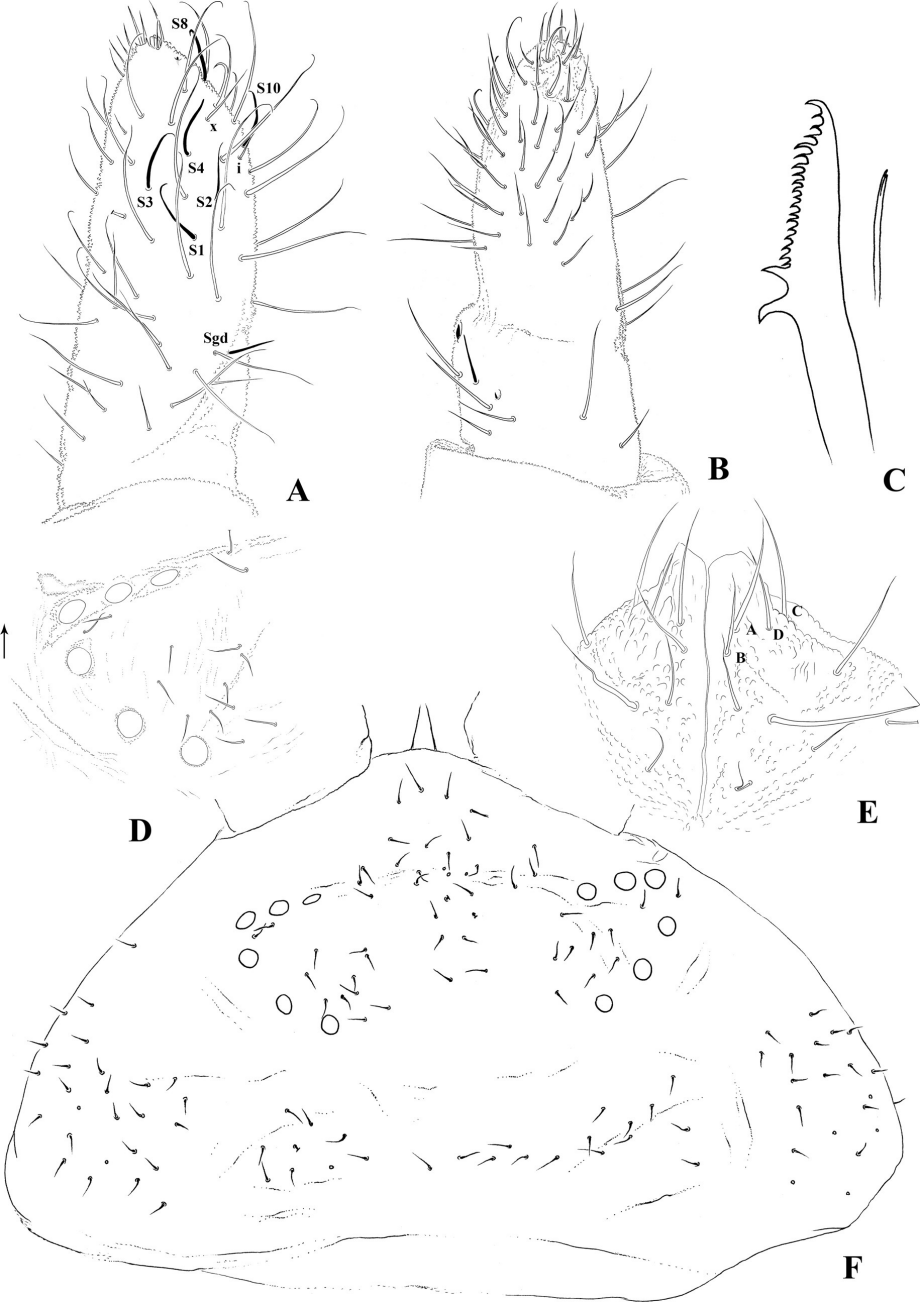
*Brasilimeria assu* sp. nov. head chaetotaxy and cephalic appendages. (A) Ant. III–IV dorsal view. (B) Ant. III–IV ventral view. (C) mandible and maxillae. (D) Detail of left eyes and smooth PAO area. (E) Labium. (F) Dorsal head chaetotaxy.

Antennae: Ant. IV presents trilobed apical bulb; subapical organite present; ms absent; x chaetae between a1 and a3; six S-chaetae: S1–S4, S8 and S10; a set of three to five thickened chaetae located dorsolaterally next to S10; no ventral sensorial field (Fig 2A and 2B). AIIIO composed of five S-chaetae: Sgd and Sgv sub-equal and smaller than Ant. IV S-chaetae, two small internal rod-like S-microchaetae in a tegument depression, with ventral S-microchaeta (Fig 2B). S-chaetae are slender, and little differentiated from ordinary chaetae. Ant. II and I respectively present 11 and 10 chaetae.

Head: Eyes 6+6, disposed in a semi-circle; pigmentation under each eye, but not in the periorbital; PAO absent yet substituted with shallow tegumentary depression composed of primary granules (Fig 2D). Dorsal head chaetotaxy: pluri-chaetotic and asymmetrical, rows of chaetae not clearly defined (Fig 2F). Labium with chaetae A–G, C and D apically displaced (Fig 2E). Mandibles: long, with about 20 teeth, presenting two strong basal teeth and about 17 smaller apical teeth, with the five most apical teeth longer than the others; maxillae: stylet-like (Fig 2C).

**Fig 3:**
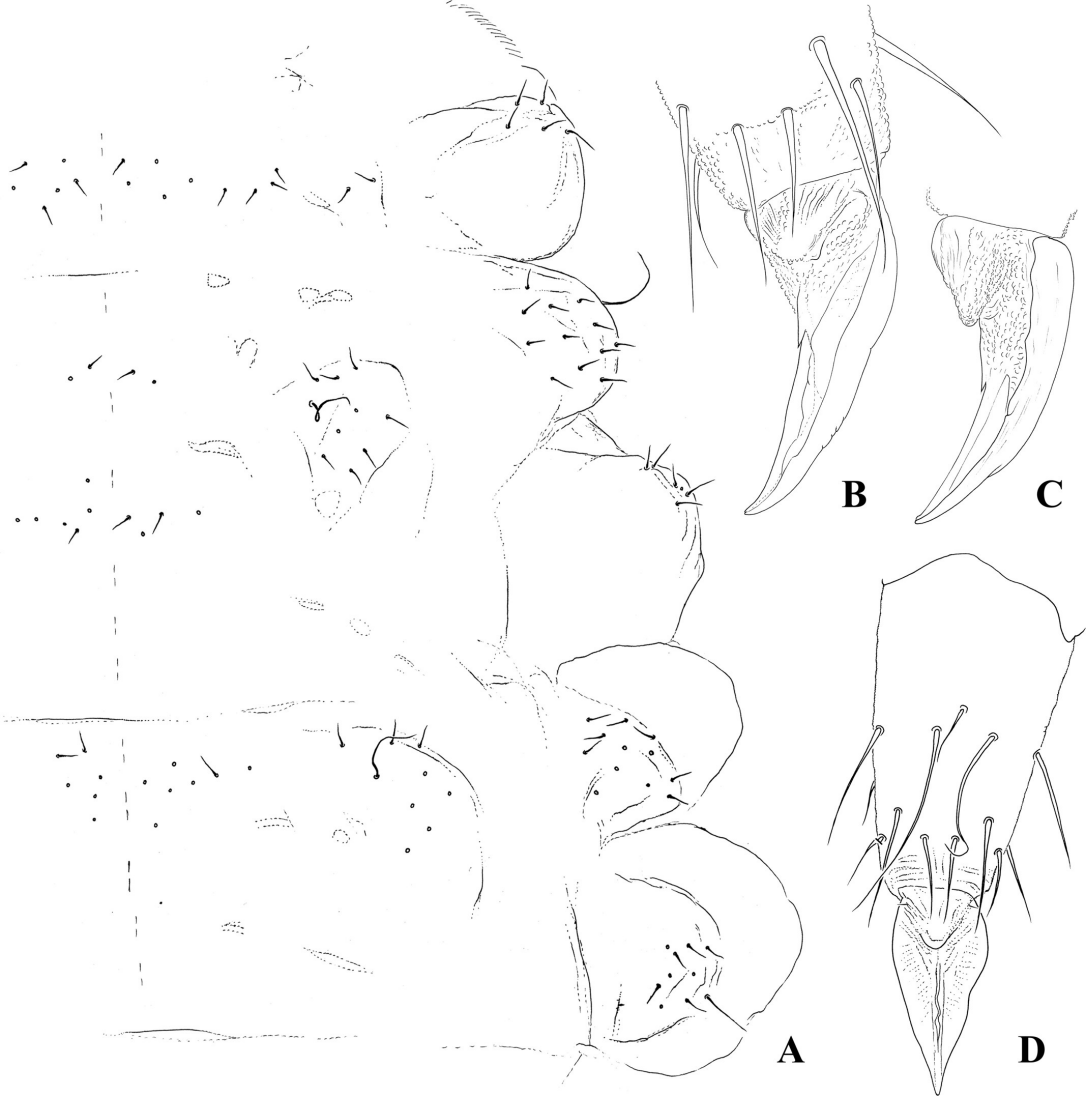
*Brasilimeria assu* sp. nov. thoracic and appendages chaetotaxy. (A) Half dorsal chaetotaxy of Th. I–III. (B) Ventral view of distal area of tibiotarsus and unguis. (C) Lateral view of unguis. (D) Ventral view of tibiotarsus and unguis.

**Fig 4:**
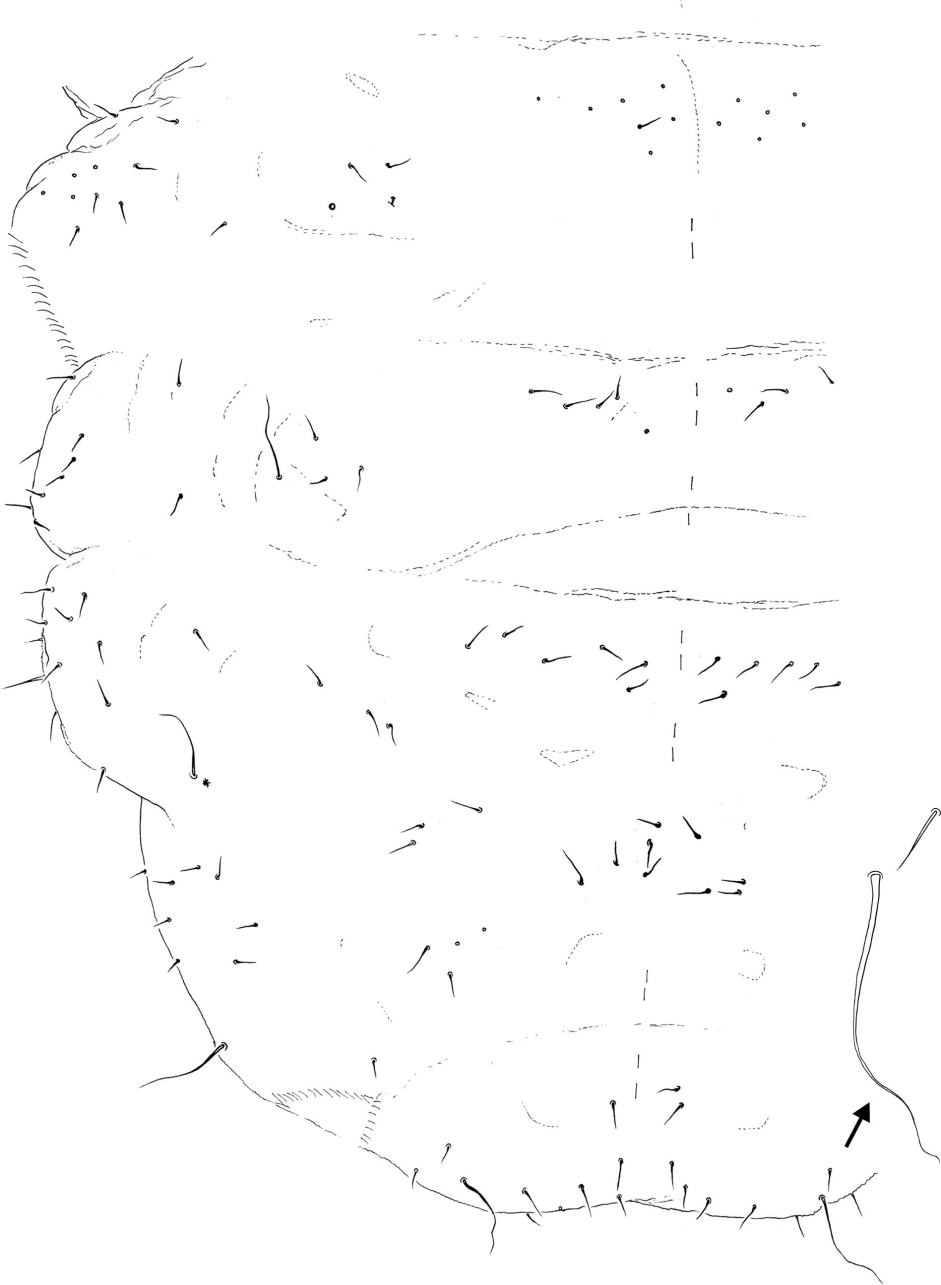
*Brasilimeria assu* sp. nov. dorsal abdominal chaetotaxy. Half dorsal chaetotaxy of Abd. I–V. Arrow indicates detail of S-chaeta and ordinary Abd. V chaeta.

Dorsal chaetotaxy: Body pluri-chaetotic, with groups of chaetae Di, De, DL and especially L presenting a high number of chaetae; L: groups of chaetae positioned in well-developed paratergites on thorax and abdomen. Th. I with 11+11 or more chaetae. Th. II and III with 6–7 Di chaetae, 8–9 + S De chaetae, DL and L apparently fused, with 12 or more chaetae + S-chaetae; ms on Th. II not visualized (Fig 3A). Abd. I–III with 5–7 Di chaetae, 3+S De chaetae, 1–2 DL chaetae, and 10 or more L chaetae on paratergites; Abd. IV presents 4–5 Di, 7 De, and 8–10 chaetae + S-chaetae on DL. Abd. V presents 6–7 Di chaetae, and 2–3 chaetae + S-chaetae on DL; Abd. VI ventral. S-chaetae formula by half tergite = 022/11111. Half-dorsal Th.I–III and Abd. I–V chaetotaxy is respectively presented in Figs 3A and 4. Ordinary chaetae are long, stiff, and almost spine-like. Ratio ordinary chaetae:S-chaetae = 1:4 (see detail of Fig 4).

Legs: Respective chaetotaxy of legs I–III, is as follows: Subcoxa I– 1,2?,2?; Subcoxa II– 0,2,2; Coxa– 3,6,7–8?; Trochanter– 6,6,6; Femur– 13,12,11; Tibiotarsus– 19,19,18. Tita I–III with M chaeta basally displaced, B4 slightly longer than B5; Claw with basal inner tooth; Claw:Tita ratio = 1:1.5 (Fig 3B–3D).

**Fig 5:**
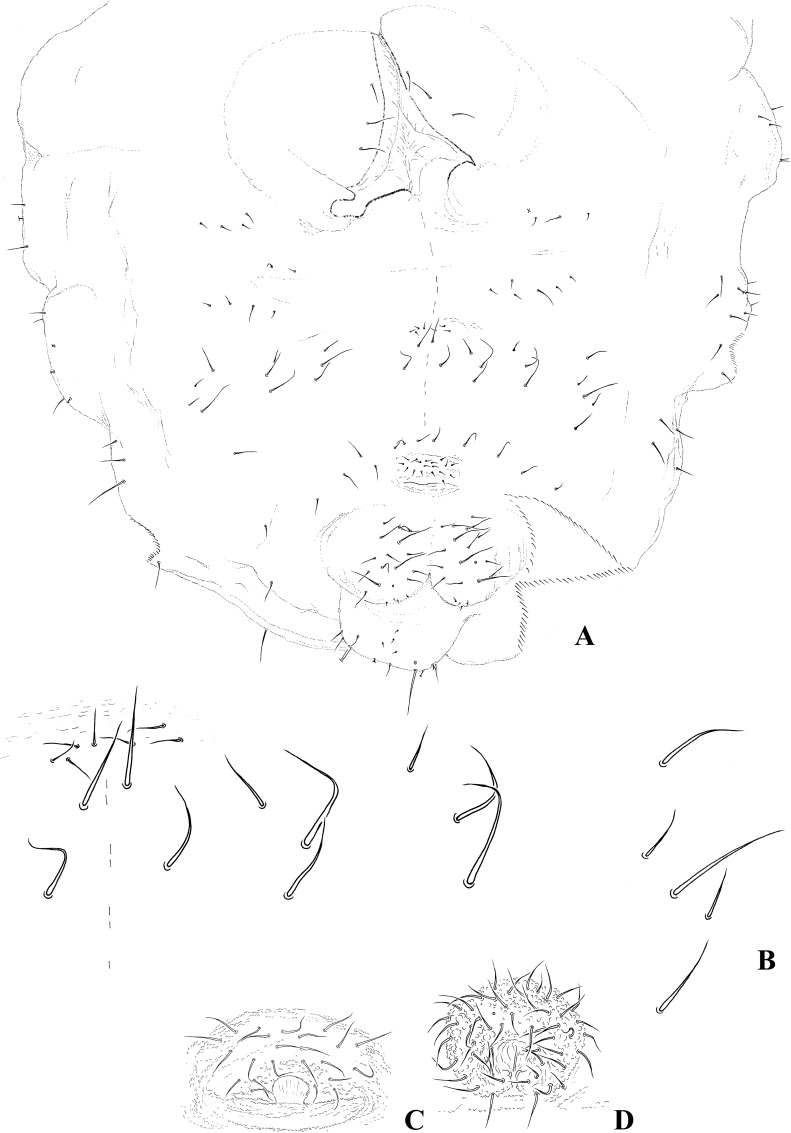
*Brasilimeria assu* sp. nov. ventral abdominal chaetotaxy. (A) Ventral view of Abd. I–VI. (B) Half chaetotaxy detail of furcal chaetae remains. (C) Female genital plate. (D) Male genital plate.

Ventral abdomen: VT presents 3+3 chaetae. Abd. II presents 4+4 chaetae. Abd. III presents 6+6 chaetae; tenaculum absent. Furca absent: 3–4+3–4 chaetae present as remains of dens, and 8+8 chaetae present as remains of manubrium; 5+5 ventrolateral chaetae on Abd. IV. Abd. V presents 5–7+5–7 ante-genital chaetae (Fig 5A); Vestigial furcal chaetae in Fig 5B. Female and male genital plate in Fig 5C and 5D, respectively. Abd. VI terminal anal valve presents 6+6 chaetae and 4 hr; ventral anal valves with 16 chaetae and 4 hr each.

#### Etymology

As a reference to one the locations where the specimens were found, “*Castelos do Açu*”, the word from the indigenous Brazilian *Tupi* language “*Açu*” Latinized to “*assu”*, which means big, was considered appropriate given the large size of the new species.

#### Remarks

Analysis of specimens deposited at the Collembola Collection of the Museu Nacional/UFRJ–Rio de Janeiro-Brazil, (two previously known species: *Brasilimeria*, *B*. *anura* and *B*. *wygodzinskyi*), revealed important characteristics not mentioned in the original descriptions. In addition to conspicuous coloration patterns that clearly characterize the species, these also presented a distinctive S-chaetae body formula: 22/22211, i.e. besides the 2+2 S-chaetae on Th. II and III they also present 2+2 S-chaetae on Abd. I–III; being one pair on the De chaetae group and another on the DL group. The new species *Brasilimeria assu* sp. nov., presents neither patches of white or yellow on its dark blue body, nor a pair of DL S-chaetae on Abd. I–III, and the new species presents rather long ordinary body chaetae; with an ordinary chaeta, S-chaeta ratio = 1:4.This remains in contrast to a ratio of at least 1:9 in the other two species (assuming equal length S-chaetae in all three species).

We note that none of the analyzed specimens present DL S-chaetae on Abd. I–III, suggesting that the absence of such chaetae; in contrast to what has been observed in other species of the genus the presence of a reduced S-chaetae formula (022/11111) is diagnostic for *Brasilimeria assu* sp. nov. Two different S-chaetae formulas are proposed as characteristic for the genus, and thus attention must be given to the presence of two pairs of S-chaetae on Abd. I–III. The formula 22/22211 is thus not restricted to Pseudachorutinae, though in Neanuridae as a whole, it is both remarkable and uncommon.

According to Queiroz & Zeppelini [12] the antennal chaetotaxy of *Brasilimeria* is similar to that of *Handschinurida*, which is corroborated in the description of *Brasilimeria assu* sp. nov. Further, antenna scheme differences encompass a group of 3–4 latero-external (almost ventral), smaller, and slender chaetae similar to S-chaetae of Ant. IV, between S8 and S10. These groups of chaetae were observed on all three species of the genus. Another remarkable feature is the number of chaetae on Ant. I, between 10–12 chaetae, higher than the usual 7 chaetae seen in the Pseudachorutinae genera [26].

An interesting age-related aspect in specimens of this genus regards their mandibles: juveniles, (generally up to 1.5mm), have mandibles with only 10–12 teeth. This was observed in about ten juvenile specimens. In fact, the description of *B*. *anura*, Arlé [27] illustrated the mandible of a juvenile with only ten teeth. Afterwards, Cassagnau & Rapoport [28] depicted a *B*. *anura* mandible based on specimens from the *Tijuca* forest in Rio de Janeiro, (its type locality) with twenty teeth. *B*. *wygodzinskyi* illustrations in turn, reveal the actual shape of an adult *Brasilimeria* mandible. This is the first report of mandible size and shape modification within Pseudachorutinae, and perhaps in Collembola as a whole throughout post-embryonic development. Within the genus *Brasilimeria* however, adult mandible shape and number of teeth is very stable, as verified in all three species.

#### Identification Key for *Brasilimeria* Stach 1949 species

**1.** Color dark blue to black over entire body; stiff and long ordinary chaetae on body, especially on paratergites; ordinary chaeta: S-chaeta = 1:4; S-chaetae formula = 022/11111… … *Brasilimeria assu* sp. nov.

**1’.** Yellow or white spots on body; very small and thin ordinary chaetae on body; ratio of ordinary chaeta:S-chaeta > 1:9; S-chaetae formula = 022/22211 ……………………………………………………………. . . . . **2**

**2.** Body color dark blue to black; with yellow Ant. III–IV. . . . . . . . . . . . . . . . . . . . . . . . . . . . . . *Brasilimeria anura* (Arlé 1939)

**2’.** Body color dark blue to black with patches of yellow on Ant. IV apex, the entire Ant. I, axially on head basis, dorso-external and dorsal-lateral spots on Th. II, dorsal-external spots on Th. III, Abd. II with both an axial spot, and a dorso-external spot extending ventrally, dorso-external small spots on Abd. IV, and dorso-external spots on Abd. V .…………………………………. . . . . . . . . . . . . . . . . *Brasilimeria wygodzinskyi* (Arlé 1943)

#### Chemical Analysis

Analyses of *Brasilimeria assu* sp. nov. secretions (GC-MS) revealed 32 constituents (Table 2) in a mixture of different classes of compounds including aliphatic and aromatic hydrocarbons, esters, alcohols, phenol, aldehyde, and ketone.

**Table 2:** Constituents identified from the hemolymphatic secretion of *Brasilimeria assu* sp. nov. (Collembola, Neanuridae) using GC-MS.

Retention Time (min)	Identified compound	Molecular weight	Relative Amount (%)	Chemical Class
4.522	Isoamyl alcohol	88	0.63	Alcohol
19.368	3,8-dimethyl-undecane	184	1.03	Hydrocarbon
19.608	3-Methyl-undecane	170	0.34	Hydrocarbon
19.697	2,4-Di-*tert*-butyl-phenol	206	2.98	Phenol
21.004	3-(*Z*)-Hexadecene	224	0.26	Hydrocarbon
21.105	*n*-Hexadecane	212	1.91	Hydrocarbon
21.841	Benzophenone	182	0.02	Ketone
22.275	1,6-dimethy-4-Isopropyl-naphthalene	198	0.57	Hydrocarbon
22.461	*n*-Nonadecane	268	0.35	Hydrocarbon
22.513	2-Ethyl-2-methyl-1-tridecanol	242	0.99	Alcohol
22.545	*n*-Heptadecane	212	1.25	Hydrocarbon
22.588	7-Methyl-heptadecane	254	0.43	Hydrocarbon
22.767	2,2,4,6,6-Pentamethyl-heptane	170	0.29	Hydrocarbon
22.832	*n*-Hexadecanal	240	0.57	Aldehyde
23.579	Benzyl Benzoate	212	46.98	Ester
23.910	*n*-Octadecane	212	3.62	Hydrocarbon
24.280	Isopropyl myristate	270	1.20	Ester
24.789	Isobutyl phthalate	278	5.29	Ester
25.225	*n*-Nonadecane	380	0.89	Hydrocarbon
25.378	1-Methyl-nonadecyl-benzene	358	1.66	Hydrocarbon
25.434	2,2,11,11-Tetramethyl-dodecane	226	0.67	Hydrocarbon
25.583	Methyl palmitate	270	2.58	Ester
26.118	Phthalic acid, butyl undecyl ester	376	1.85	Ester
26.414	Palmitic acid, ethyl ester	284	4.12	Ester
26.469	*n*-Octadecane	254	1.66	Hydrocarbon
26.792	Isopropyl-hexadecanoate	298	0.60	Ester
28.752	Stearic Acid, ethyl ester	312	4.92	Ester
31.976	Lauric acid, *n*-octyl-ester	312	0.56	Ester
32.312	Di-*n*-octyl phthalate	390	1.82	Ester
33.887	*n*-Octyl ether	242	0.66	Ether
34.895	Squalene	410	8.46	Hydrocarbon
37.671	9-(*Z*)-Hexadecenoic acid, octadecyl ester	507	0.83	Ester

Most of the compounds were aliphatic hydrocarbons and medium-long chain esters (C10-C20). There were also two alcohols (isoamyl alcohol and 2-ethyl-2-methyl-1-tridecanol), 1 phenol (2,4 di-*tert*-buthyl-phenol), 1 aldehyde (*n*-hexadecanal) and 1 ketone (benzophenone). All of the molecular ion peaks [M^•+^], base peaks, and respective fragmentations for all of the identified compounds are available in the supplementary material.

The main constituents, which presented larger areas were: benzyl benzoate (46.98%), squalene (8.46%), isobutyl phthalate (5.29%), stearic acid, ethyl ester (4.92%), palmitic acid, ethyl ester (4.12%), *n*-octadecane (3.62%), and 2,4-di-*tert*-butyl-phenol (2.98%).

Benzyl benzoate is the main constituent of *Cananga odorata* (Lam.) Hook & Thomson (ylang-ylang) [29] and *Aniba hostmanniana* (Nees) Mez (29.3%; GC-MS) essential oils [30], of volatile oils isolated from *Solanum xanthocarpum* Schrad & Wendl fruit (21.7%; GC-MS) [31], and from *Senna hirsuta* (L.), Irwin & Barneby (24.7%; GC-MS) [32]. Benzyl benzoate is a natural product known to be found in the essential oils of rosewood (*Dalbergia sissoo* L.), cinnamon (*Cinnamomun verum* Presl) and in balsam of Peru (*Myroxylon balsamum* (L.) Harms).

Benzyl benzoate is used in insect and acari repellents, and for chiggers, ticks and mosquitoes [33, 34]. As medicine used to treat scabies and lice [35, 36], it has been known for more than 100 years [34]. Recent studies have shown that cinnamon oil (containing benzyl benzoate 9.99%) presents insecticidal activity against *Tenebrio molitor* Linnaeus, 1758 [37] and *Sitophilus granarius* (Linnaeus, 1758) affecting respiratory rates and hindering or reducing insect mobility [38].

In our study of *Brasilimeria assu* sp. nov. secretions we also identified long chain fatty acids: palmitic acid (C_16_) and stearic acid (C_18_), in their esterified forms, being methyl, ethyl, and isopropyl esters of palmitic acid, and an ethyl ester of stearic acid. In a study conducted by Dettner *et al*. [4], these same long chain fatty acids (palmitic acid and stearic acid) were identified by GC-MS analysis as major components. Dettner *et al*. [4] also identified three pyrido-pyridazine alkaloids: 2,3-dimethoxy-pyrido[2,3-*b*]pyridazine, 3-*iso*propyl-2-methoxy-pyrido[2,3-*b*]pyridazine, and 2-methoxy-4*H*-pyrido[2,3-*b*]pyridazine in the pseudocellar fluid of *Tetrodontophora bielanensis* (Waga, 1842), which has been shown to present repellent properties against *Nebria brevicolis* (Fabricius, 1792) (a common predator), causing beetle disorientation and cleansing behavior.

Certain aromatic substances were identified in *Brasilimeria assu* sp. nov. secretions such as the benzene derivative (1-methyl-nonadecyl-benzene), benzophenone, and phenol (2,4-di-*tert*-butyl-phenol), which are also chemical constituents of *Catunaregam spinosa* (Thunb.) Tirveng essential oil, (mountain pomegranate) [39]; the fruit presents toxicity to fish [40] and known emetic effects. Messer et al. [7], identified the aromatic compounds (2,4-dimethoxyaniline, 1,3-dimethoxybenzene, 2-aminophenol, phenol) in *Neanura muscorum* (Templeton, 1835) (Collembola, Neanuridae). Of these compounds, 2-aminophenol presents repellent properties against *Pergamasus norvegicus* (Berlese, 1905) (Acarina, Parasitidae).

The genus *Brasilimeria* is characterized by the absence of furca (ventral abdominal appendages of segment IV which are modified for jumping), and by large adult specimens, being generally longer than 3 mm, and even reaching 4 mm. The species thus presents reduced mobility and little potential for predator evasion, depending entirely on short cursorial legs that result in clumsy movements. In this sense, the large presence of insecticidal and acaricide substances, such as benzyl benzoate, may be considered as a great ecological/evolutionary advantage against predators.

*Brasilimeria assu* sp. nov., and the two previously known species of the genus, *B*. *anura* (Arlé, 1939) and *B*. *wygodzinskyi* (Arlé, 1943), have distinctive color patterns: being a dark blue body contrasted by bright yellow or white spots in differing arrangements over the whole body, this with a bright ventral body and appendages.

As pointed out by Bellinger, such color patterns can be an aposematic indication for unpalatability [41]. The Neotropics present many brightly colored and patterned Pseudachorutinae species (see Oliveira & Deharveng [42] for *Pseudachorutes* Tullberg, 1871 species; and Queiroz & Mendonça [43] for *Arlesia* Handschin, 1942, and *Handschinurida* Queiroz, 2015 species). Nevertheless, in order to relate unpalatable or repellent components in the hemolymph to aposematism, additional comparative studies will be required.

The presence of benzyl benzoate, in *Brasilimeria assu* sp. nov. secretions is primarily acknowledged as a consequence of food ingestion and consequent incorporation to the hemolymph. In addition to other substances, decaying leaves and twigs of plants that synthesize the compound may be ingested by the species. This is corroborated by the distribution of two plant species that produce benzyl benzoate, *Myroxylon balsamum* (L.) Harms, and *S*. *hirsuta*; as well as many species of the genus *Solanum* [44], which are congruent with that of *Brasilimeria assu* sp. nov.

## Conclusions

The taxonomic description of *Brasilimeria assu* sp. nov., together with analyses of *B*. *anura*, and *B*. *wygodzinskyi* revealed for the first time (within Collembola) important post-embryonic modifications in the shape and size of the mandibles, with teeth numbers increasing from juveniles to adults. Other original data involved the presence of ten or more chaetae on (Ant. I) of *Brasilimeria*, as well as *B*. *anura* and *B*. *wygodzinskyi* which present two pairs of S-chaetae on (Abd. I–III); being unique features within Neanuridae. Detailed analyses and descriptions of larger species in the tropics, such as those of *Brasilimeria*, will be needed for a better understanding of Pseudachorutinae taxonomic characters as well as generating reliable evolutionary scenarios and phylogenetic hypotheses.

Analysis of *Brasilimeria assu* sp. nov., secretions (using GC-MS) permitted detection of 32 constituents. The major component at 46.98% of the sample volume was benzyl benzoate, which is known for its insecticidal action. It represents a defense mechanism against predation.

Our results on the chemical composition of *Brasilimeria assu* sp. nov., secretion permit future comparisons of metabolites secreted by other Collembola species, and allow conclusions to be drawn regarding both chemotaxonomic classification and evolutionary history.

## Supporting information

S1 Supporting information Gas Chromatography/Mass Spectrometry (CG-MS) conditions. (PDF)

S2 Supporting information Gas Chromatogram of the hemolynphatic secretion of *Brasilimeria assu* sp. nov. with all peaks, and Mass Spectrometry (MS) of main peaks with fragmentation. (PDF)
