# Supplementary material for: Chemical analysis of Brasilimeria Stach, 1949 (Hexapoda, Collembola, Neanuridae) hemolymphatic secretion, and description of a new species
Source: PLoS One. 2019 Feb 21;14(2):e0212451. doi: 10.1371/journal.pone.0212451 (PMC6383892; doi:10.1371/journal.pone.0212451)

Sample Information

Analyzed by : Admin
Analyzed : 22/11/2017 14:54:07
Sample Type : Unknown
Level # : 1
Sample Name : 2304_CM/MN_hemolinfa
Sample ID : 2304_CM/MN_hemolinfa
IS Amount : [1]=1
Sample Amount : 1
Dilution Factor : 1
Vial # : 1
Injection Volume : 1.00
Data File : C:\Amostras GCMS\Norberto\Hemolinfa\2304_CMMN_hemolinfa3.qgd
Org Data File : C:\Amostras GCMS\Norberto\Hemolinfa\2304_CMMN_hemolinfa3.qgd
Method File : C:\Amostras GCMS\Norberto\R. Adams.qgm
Org Method File : C:\Amostras GCMS\Norberto\R. Adams.qgm
Report File :
Tuning File : C:\GCMSsolution\System\Tune1\2017\17-11-2017.qgt
Modified by : Admin
Modified : 24/11/2017 14:20:09

Method

Gas Chromatograph Mass Spectrometer- Shimadzu -
Model: QP-2010.

Column: DB-5MS (30m x 0.25mm x 0.25um) Agilent Technologies

===== Analytical Line 1 =====

[GC-2010]
Column Oven Temp. :40.0 °C
Injection Temp. :260.00 °C
Injection Mode :Splitless
Sampling Time :2.00 min
Flow Control Mode :Linear Velocity
Pressure :77.3 kPa
Total Flow :10.0 mL/min
Column Flow :1.40 mL/min
Linear Velocity :42.7 cm/sec
Purge Flow :3.0 mL/min
Split Ratio :4.0
High Pressure Injection :OFF
Carrier Gas Saver :ON
Carrier Gas Saver Split Ratio :5.0
Carrier Gas Saver Time :1.00 min
Oven Temp. Program

Rate	Temperature(°C)	Hold Time(min)
-	40.0	4.00
8.00	320.0	20.00

< Ready Check Heat Unit >
Column Oven : Yes
SPL1 : Yes
MS : Yes
< Ready Check Detector(FTD) >
< Ready Check Baseline Drift >
< Ready Check Injection Flow >
SPL1 Carrier : Yes

SPL1 Purge : Yes
< Ready Check APC Flow >
< Ready Check Detector APC Flow >
External Wait :No
Equilibrium Time :1.0 min

[GCMS-QP2010]
IonSourceTemp :250.00 °C
Interface Temp. :320.00 °C
Solvent Cut Time :3.50 min
Detector Gain Mode :Relative
Detector Gain :+0.00 kV
Threshold :1000

[MS Table]
--Group 1 - Event 1--
Start Time :4.50min
End Time :59.00min
ACQ Mode :Scan
Event Time :0.25sec
Scan Speed :2500
Start m/z :35.00
End m/z :600.00

Sample Inlet Unit :GC

[MS Program]
Use MS Program :OFF

Tuning

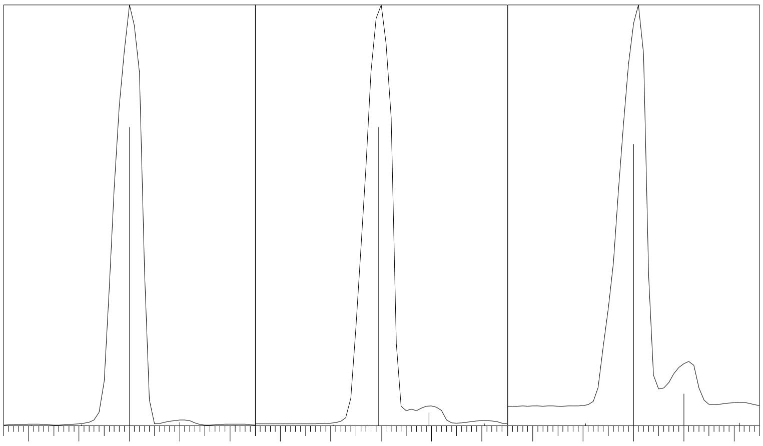

m/z : 69.00	m/z : 219.00	m/z : 502.00
Factor : 12.10	Factor : 32.26	Factor : 308.85
Inten. : 614786	Inten. : 230743	Inten. : 22734
Ratio. : 100.00	Ratio. : 37.53	Ratio. : 3.70
FWHM : 0.60	FWHM : 0.60	FWHM : 0.59

Ionization Mode : EI
Tuning Date : 17/11/2017 10
Filament# : 1
Lens1 : -0.3 V
Lens2 : -26.0 V
Lens3 : -0.3 V
Lens4 : -17.0 V
RF Gain : 4701
RF Offset : 4924
Pre-rod : -3.5 V
Deflector : -20.0 V
Detector : 1.27 kV

Ionization voltage : 70 V
Emission current : 60 uA
Main-rod : -3.5 V
Conversion dynode: -10 kV

IonSourceTemp : 250 °C
Low Vacuum : 7.4e+000 Pa
High Vacuum : 5.9e-004 Pa
Interface Temp. : 260 °C
Oven Temp. : 70 °C
Column Pressure : 84 kPa
Column Flow : 1.3 mL/min
Column Diameter : 0.25 mm
Column Length : 30.0 m

Scan Range: 10.00 - 700.00 Scan Speed : 78
Base Peak : 69.00 (605097)

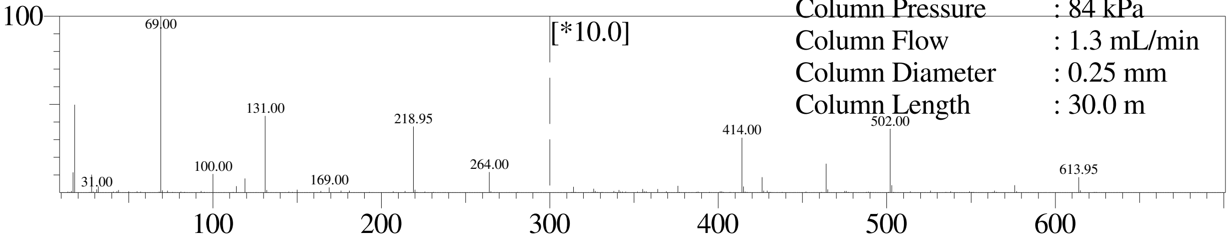

Supplement: S1 Supporting information — (PDF) [file pone.0212451.s001.pdf]
