# Supplementary material for: Chemical analysis of Brasilimeria Stach, 1949 (Hexapoda, Collembola, Neanuridae) hemolymphatic secretion, and description of a new species
Source: PLoS One. 2019 Feb 21;14(2):e0212451. doi: 10.1371/journal.pone.0212451 (PMC6383892; doi:10.1371/journal.pone.0212451)

Sample Information

Analyzed by	: Admin
Analyzed	: 22/11/2017 14:54:07
Sample Type	: Unknown
Level #	: 1
Sample Name	: 2304_CM/MN_hemolinfa
Sample ID	: 2304_CM/MN_hemolinfa
IS Amount	: [1]=1
Sample Amount	: 1
Dilution Factor	: 1
Vial #	: 1
Injection Volume	: 1.00
Data File	: C:\Amostras GCMS\Norberto\Hemolinfa\2304_CMMN_hemolinfa3.qgd
Org Data File	: C:\Amostras GCMS\Norberto\Hemolinfa\2304_CMMN_hemolinfa3.qgd
Method File	: C:\Amostras GCMS\Norberto\R. Adams.qgm
Org Method File	: C:\Amostras GCMS\Norberto\R. Adams.qgm
Report File	:
Tuning File	: C:\GCMSsolution\System\Tune1\2017\17-11-2017.qgt

$EndIf$Modified by	: Admin
Modified	: 23/11/2017 17:41:59

Chromatogram 2304_CM/MN_hemolinfa

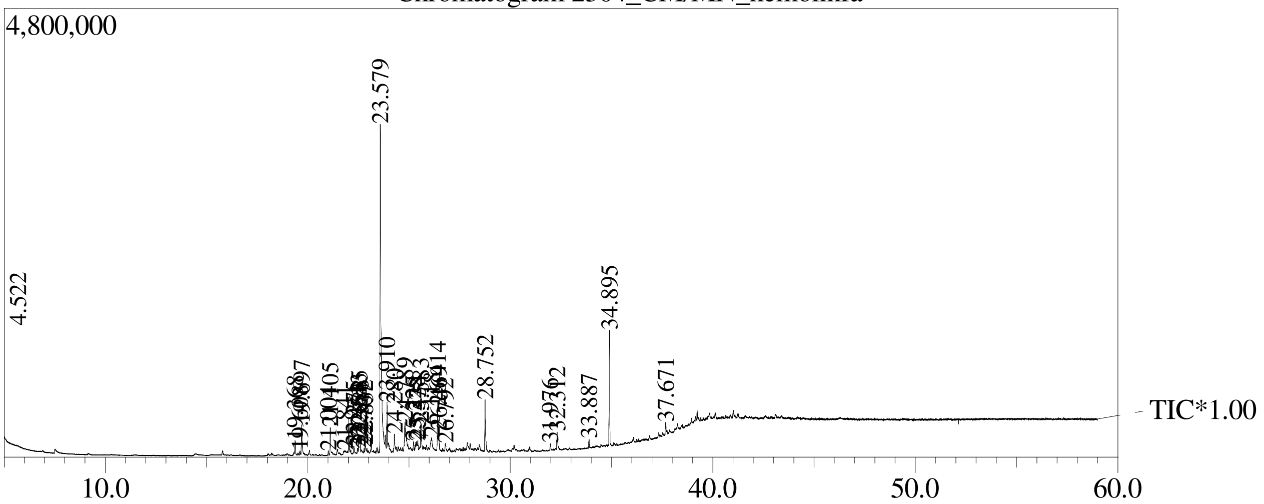

Peak Report TIC

Peak#	R.Time	Area	Area%	Name
1	4.522	171743	0.63	Isoamyl alcohol
2	19.368	282181	1.03	Undecane, 3,8-dimethyl-
3	19.608	92108	0.34	Undecane, 3-methyl- (CAS) 3-Methylun[illegible]
4	19.697	815128	2.98	Phenol, 2,4-bis(1,1-dimethylethyl)-
5	21.004	72220	0.26	3-Hexadecene, (Z)- (CAS)
6	21.105	521622	1.91	Hexadecane (CAS) n-Hexadecane
7	21.841	6284	0.02	Benzophenone
8	22.275	157066	0.57	Naphthalene, 1,6-dimethyl-4-(1-methyle[illegible]
9	22.461	95563	0.35	Nonadecane
10	22.513	272111	0.99	Tridecanol, 2-ethyl-2-methyl-

Peak#	R.Time	Area	Area%	Name
11	22.545	340849	1.25	Heptadecane (CAS) n-Heptadecane
12	22.588	117429	0.43	Heptadecane, 7-methyl-
13	22.767	79732	0.29	Heptane, 2,2,4,6,6-pentamethyl- (CAS) 2
14	22.832	156569	0.57	Hexadecanal
15	23.579	12858256	46.98	Benzyl Benzoate
16	23.910	991816	3.62	Octadecane (CAS) n-Octadecane
17	24.280	328042	1.20	Isopropyl myristate
18	24.789	1448328	5.29	1,2-Benzenedicarboxylic acid, bis(2-metl
19	25.225	243199	0.89	Nonadecane (CAS) n-Nonadecane
20	25.378	454915	1.66	Benzene, (1-methylnonadecyl)- (CAS) E
21	25.434	183655	0.67	DODECANE, 2,2,11,11-TETRAMETHY
22	25.583	707318	2.58	Hexadecanoic acid, methyl ester (CAS) M
23	26.118	507282	1.85	Phthalic acid, butyl undecyl ester
24	26.414	1126541	4.12	Hexadecanoic acid, ethyl ester (CAS) Etl
25	26.469	454722	1.66	
26	26.792	164222	0.60	Hexadecanoate <isopropyl->
27	28.752	1346347	4.92	Octadecanoic acid, ethyl ester
28	31.976	153566	0.56	LAURIC ACID, N-OCTYL ESTER
29	32.312	497692	1.82	Di-n-octyl phthalate
30	33.887	181557	0.66	Octane, 1,1'-oxybis- (CAS) n-Octyl ether
31	34.895	2314154	8.46	Squalene
32	37.671	227503	0.83	9-Hexadecenoic acid, octadecyl ester, (Z)
		27369720	100.00	

Library

<< Target >>
Line#:1 R.Time:19.367(Scan#:3569) MassPeaks:26
RawMode:Averaged 19.363-19.371(3568-3570) BasePeak:57.05(25952)
BG Mode:Calc. from Peak

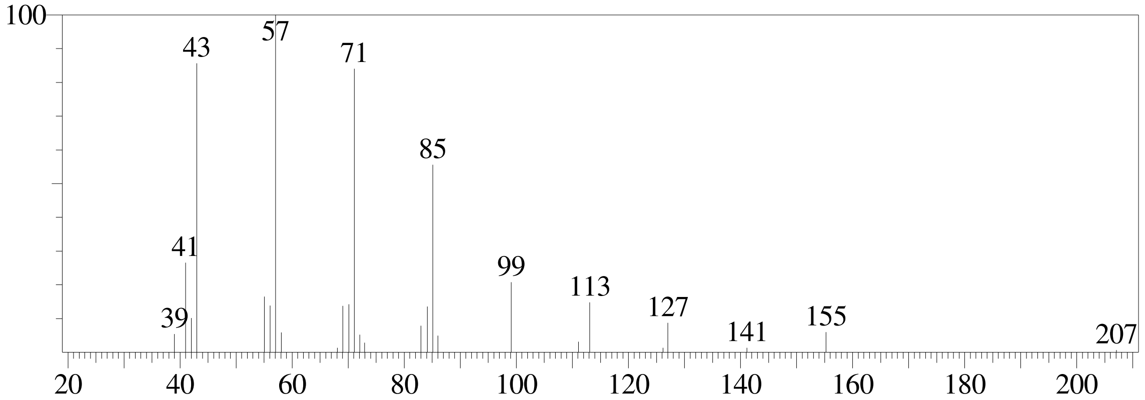

Hit#:1 Entry:34157 Library:NIST11.lib
SI:93 Formula:C13H28 CAS:17301-30-3 MolWeight:184 RetIndex:1185
CompName:Undecane, 3,8-dimethyl- $$ 3,8-Dimethylundecane # $$

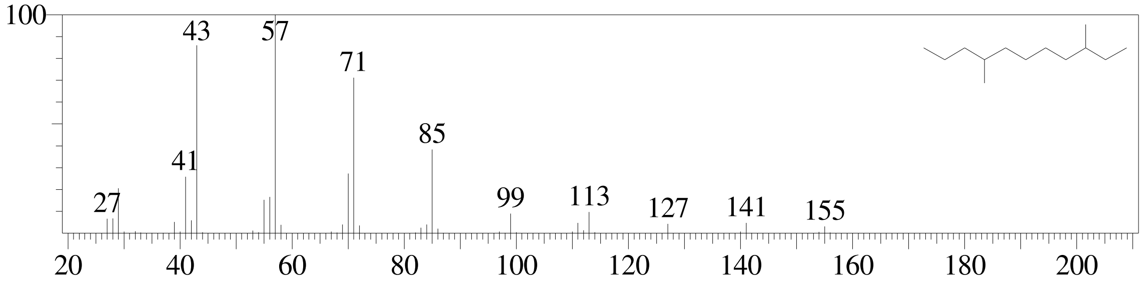

Hit#:2 Entry:76415 Library:WILEY7.LIB
SI:93 Formula:C13 H28 CAS:17301-30-3 MolWeight:184 RetIndex:0
CompName:Undecane, 3,8-dimethyl- (CAS)

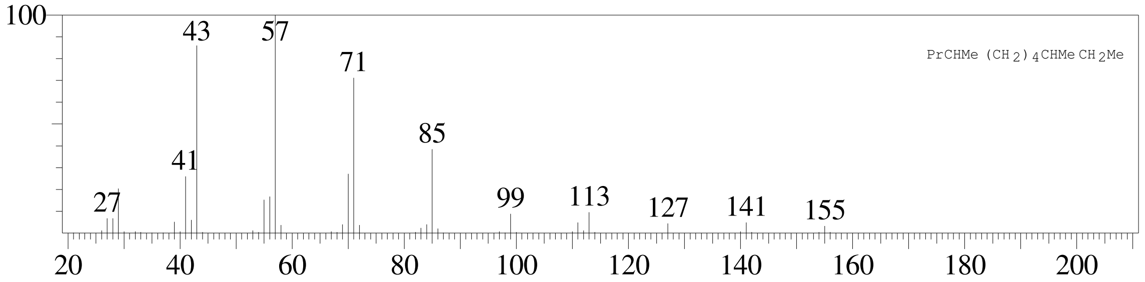

Hit#:3 Entry:42961 Library:NIST11.lib
SI:92 Formula:C14H30 CAS:61141-72-8 MolWeight:198 RetIndex:1285
CompName:Dodecane, 4,6-dimethyl- $$ 4,6-Dimethyldodecane # $$

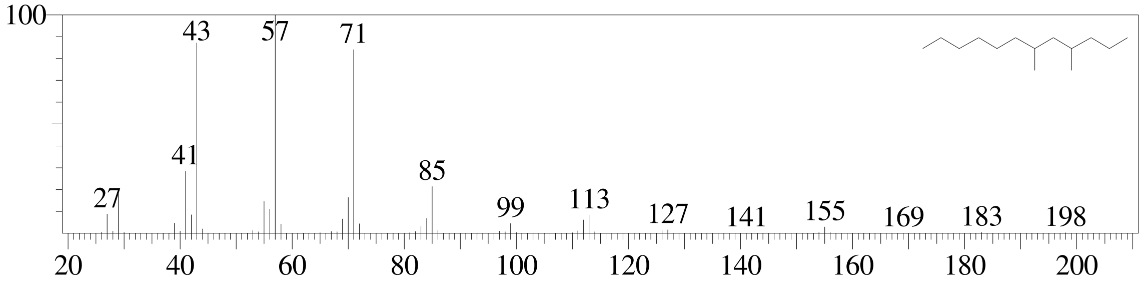

<< Target >>
Line#:2   R.Time:19.608(Scan#:3627) MassPeaks:20
RawMode:Averaged 19.604-19.613(3626-3628) BasePeak:57.10(10086)
BG Mode:Calc. from Peak

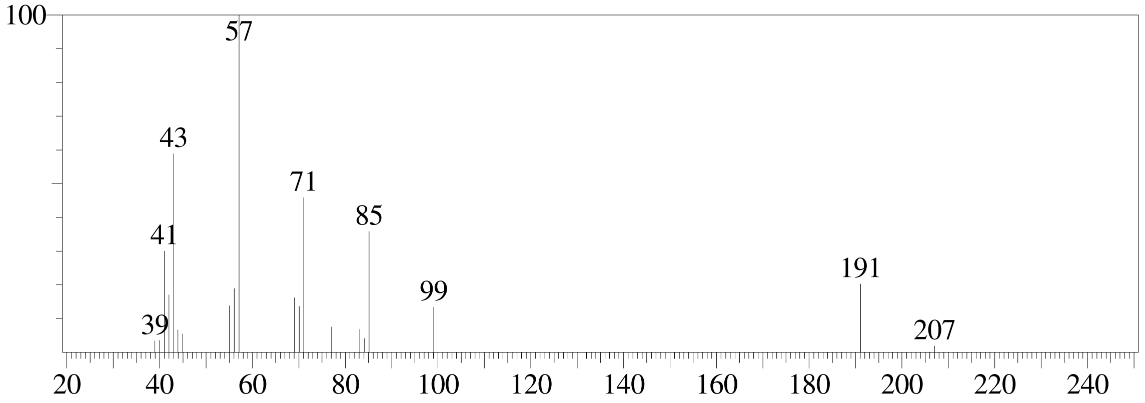

Hit#:1 Entry:61347 Library:WILEY7.LIB
SI:87 Formula:C12 H26 CAS:1002-43-3 MolWeight:170 RetIndex:0
CompName:Undecane, 3-methyl- (CAS) 3-Methylundecane $$

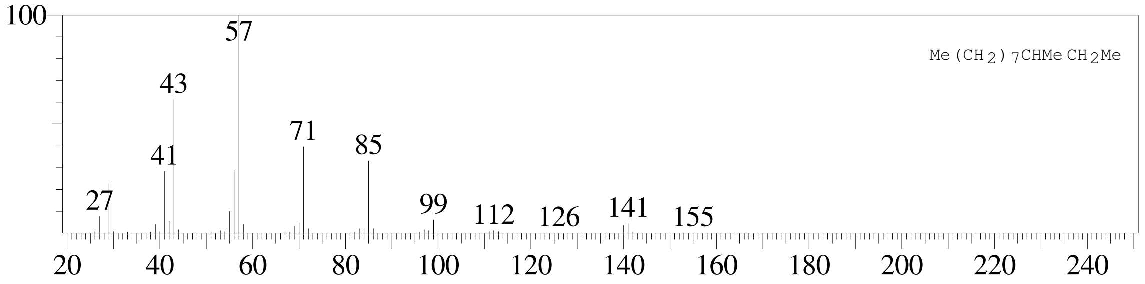

Hit#:2 Entry:146232 Library:WILEY7.LIB
SI:87 Formula:C17 H36 CAS:629-78-7 MolWeight:240 RetIndex:0
CompName:Heptadecane (CAS) n-Heptadecane $$ Normal-heptadecane $$

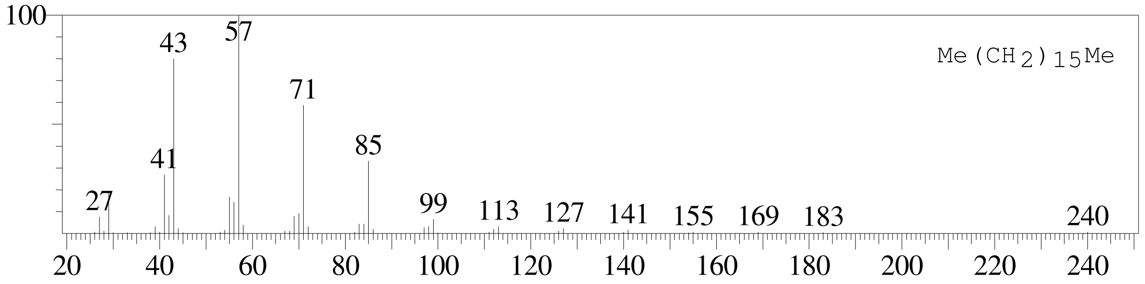

Hit#:3 Entry:647 Library:FFNSC1.3.lib
SI:87 Formula:C10 H22 CAS:124-18-5 MolWeight:142 RetIndex:1000
CompName:Decane <n->

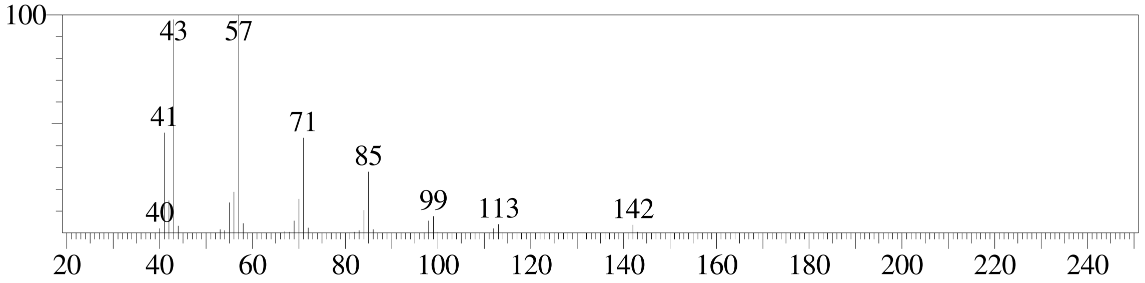

<< Target >>
Line#:3 R.Time:19.696(Scan#:3648) MassPeaks:62
RawMode:Averaged 19.692-19.700(3647-3649) BasePeak:191.10(89965)
BG Mode:Calc. from Peak

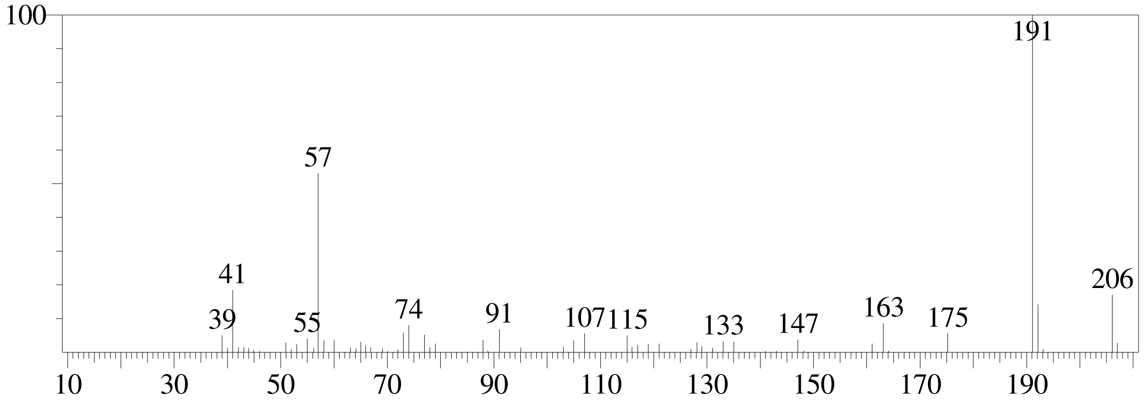

Hit#:1 Entry:48049 Library:NIST11.lib
SI:93 Formula:C14H22O CAS:96-76-4 MolWeight:206 RetIndex:1555
CompName:Phenol, 2,4-bis(1,1-dimethylethyl)- $$ Phenol, 2,4-di-tert-butyl- $$ 2,4-Di-tert-butylphe

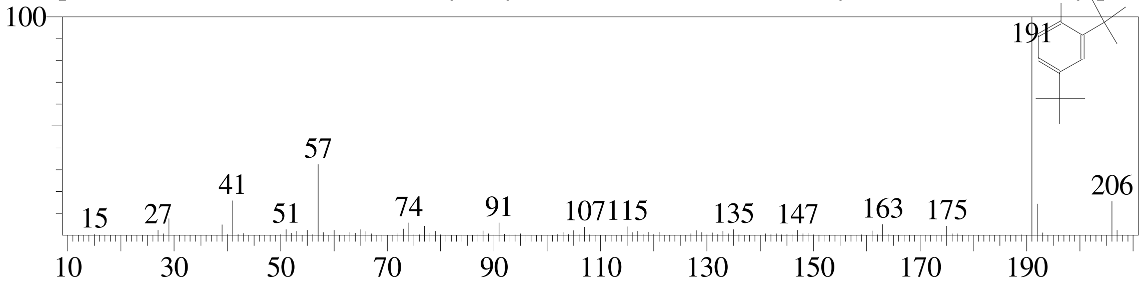

Hit#:2 Entry:18383 Library:NIST11s.lib
SI:93 Formula:C14H22O CAS:1138-52-9 MolWeight:206 RetIndex:1555
CompName:Phenol, 3,5-bis(1,1-dimethylethyl)- $$ Phenol, 3,5-di-tert-butyl- $$ 3,5-Di-tert-butylphe

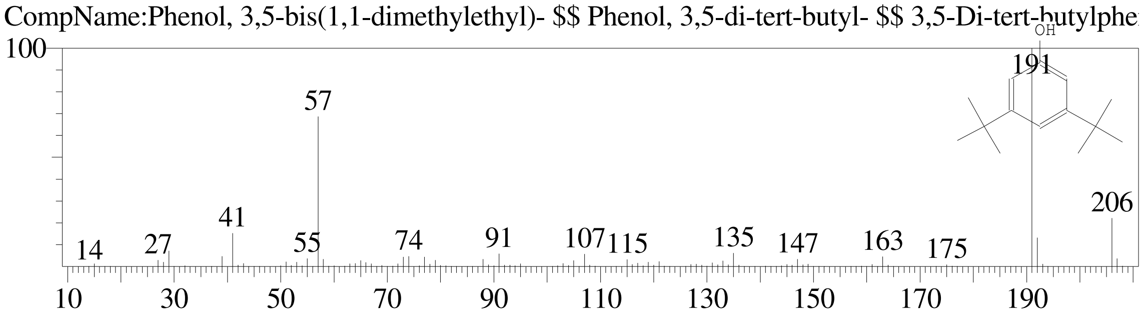

Hit#:3 Entry:103643 Library:WILEY7.LIB
SI:93 Formula:C14 H22 O CAS:96-76-4 MolWeight:206 RetIndex:0
CompName:Phenol, 2,4-bis(1,1-dimethylethyl)- (CAS) 2,4-Di-tert-butylphenol $$ 2,4-BIS(TERT-BU

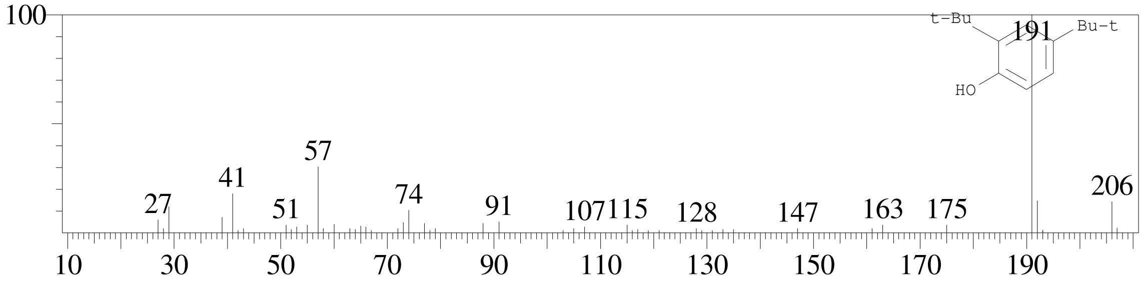

<< Target >>
Line#:4   R.Time:21.004(Scan#:3962) MassPeaks:22
RawMode:Averaged 21.000-21.008(3961-3963) BasePeak:43.05(3952)
BG Mode:Calc. from Peak

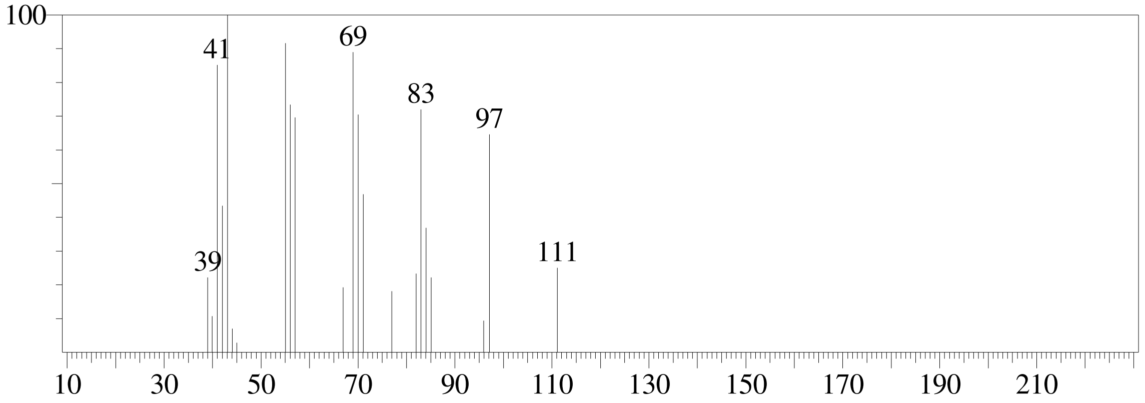

Hit#:1 Entry:126345 Library:WILEY7.LIB
SI:89 Formula:C16 H32 CAS:34303-81-6 MolWeight:224 RetIndex:0
CompName:3-Hexadecene, (Z)- (CAS)

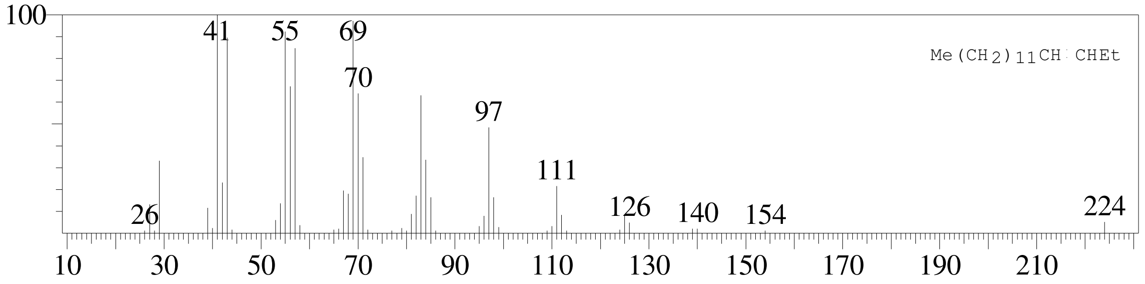

Hit#:2 Entry:14796 Library:NIST11s.lib
SI:89 Formula:C13H26 CAS:2437-56-1 MolWeight:182 RetIndex:1304
CompName:1-Tridecene $$ n-Tridec-1-ene $$ 1-C13H26 $$ Tridecene-1 $$ .alpha.-Tridecene $$ Tr

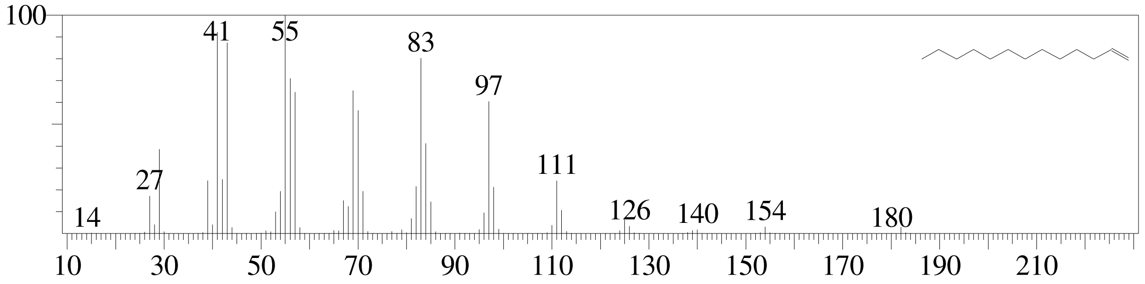

Hit#:3 Entry:60963 Library:NIST11.lib
SI:89 Formula:C16H32 CAS:34303-81-6 MolWeight:224 RetIndex:1620
CompName:3-Hexadecene, (Z)- $$ (3Z)-3-Hexadecene $$ cis-3-Hexadecene $$ (Z)-3-Hexadecene $

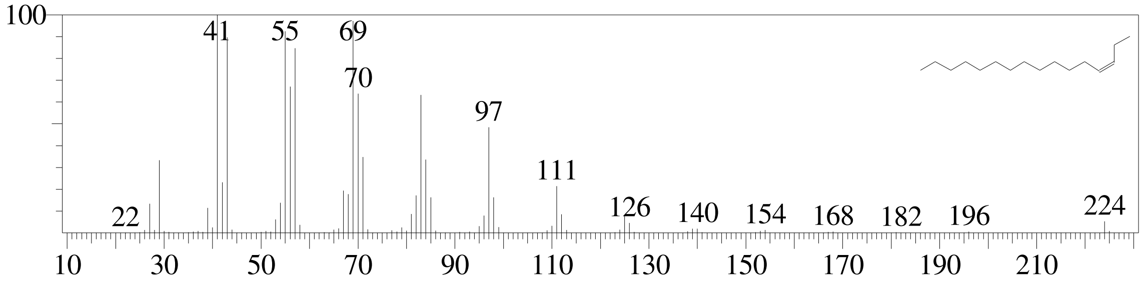

<< Target >>
Line#:5   R.Time:21.104(Scan#:3986) MassPeaks:38
RawMode:Averaged 21.100-21.108(3985-3987) BasePeak:57.05(60591)
BG Mode:Calc. from Peak

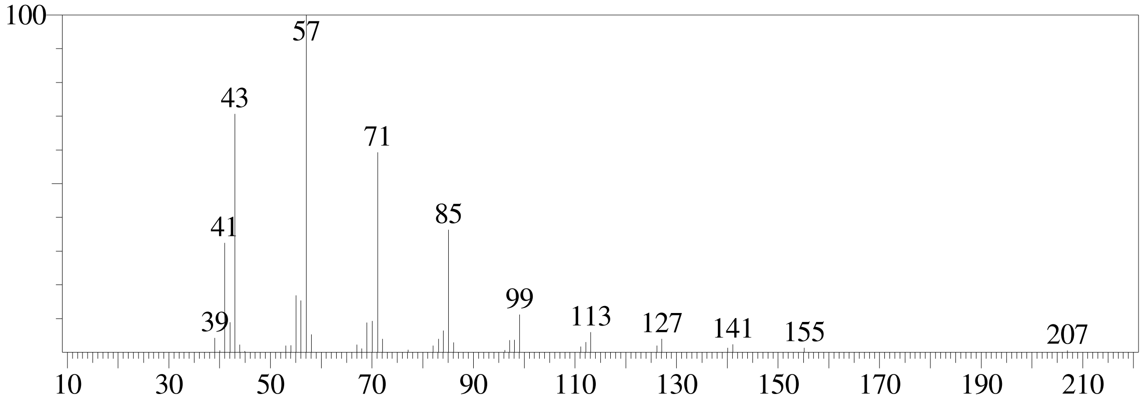

Hit#:1 Entry:111398 Library:WILEY7.LIB
SI:98 Formula:C15 H32 CAS:629-62-9 MolWeight:212 RetIndex:0
CompName:Pentadecane (CAS) n-Pentadecane $$ CH3(CH2)13CH3 $$

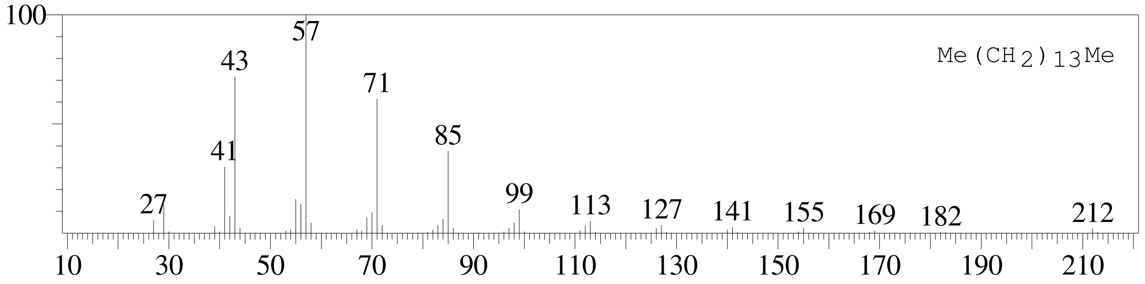

Hit#:2 Entry:795 Library:FFNSC1.3.lib
SI:97 Formula:C14 H30 CAS:629-59-4 MolWeight:198 RetIndex:1400
CompName:Tetradecane <n->

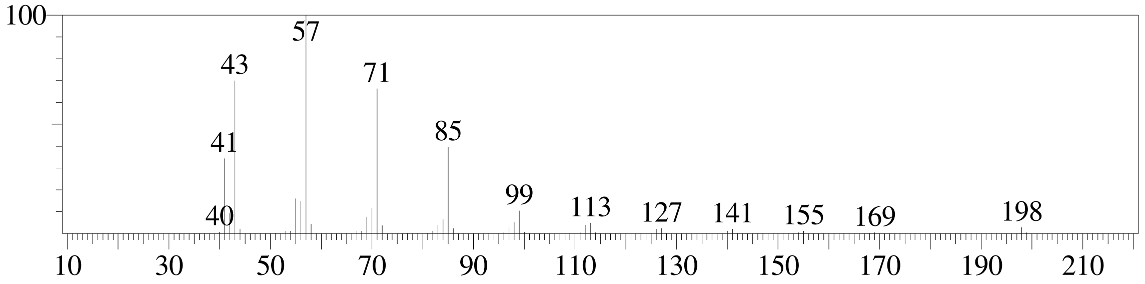

Hit#:3 Entry:52483 Library:NIST11.lib
SI:97 Formula:C15H32 CAS:629-62-9 MolWeight:212 RetIndex:1512
CompName:Pentadecane $$ n-Pentadecane $$ CH3(CH2)13CH3 $$

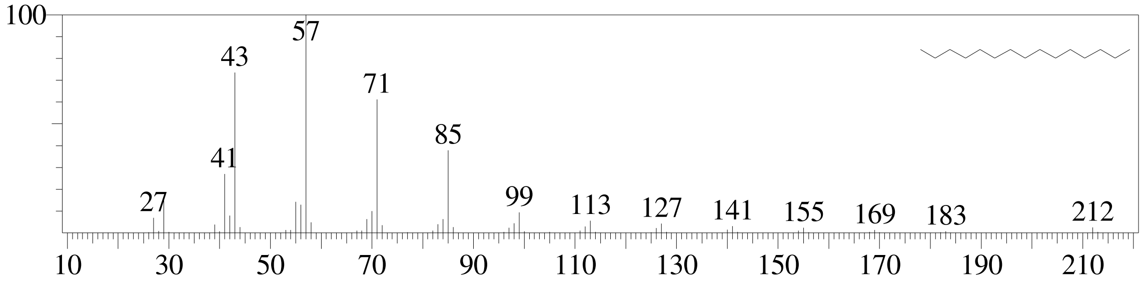

<< Target >>
Line#:6   R.Time:22.275(Scan#:4267) MassPeaks:40
RawMode:Averaged 22.271-22.279(4266-4268) BasePeak:183.10(15908)
BG Mode:Calc. from Peak

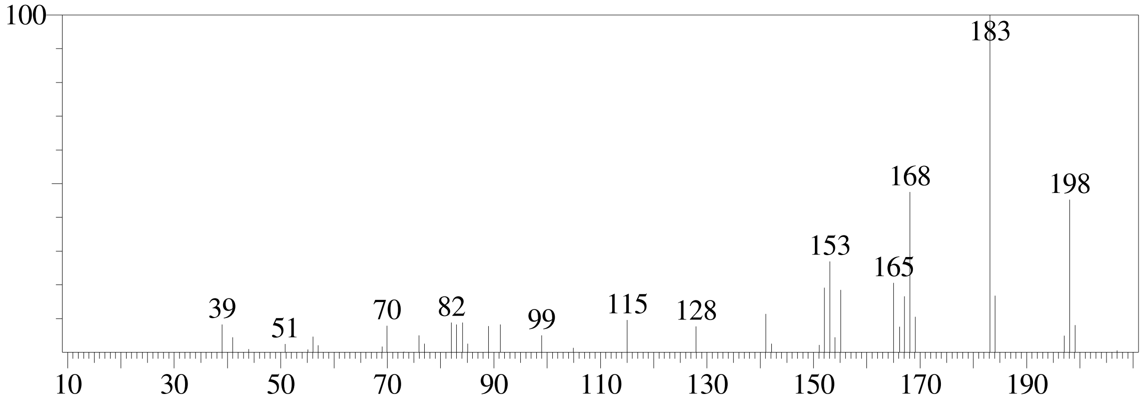

Hit#:1 Entry:17335 Library:NIST11s.lib
SI:87 Formula:C15H18 CAS:483-78-3 MolWeight:198 RetIndex:1706
CompName:Naphthalene, 1,6-dimethyl-4-(1-methylethyl)- $$ Naphthalene, 4-isopropyl-1,6-dimethy

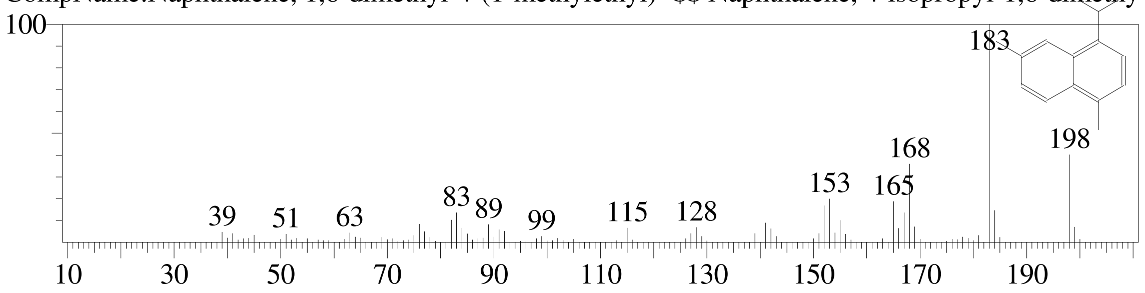

Hit#:2 Entry:42975 Library:NIST11.lib
SI:86 Formula:C15H18 CAS:483-78-3 MolWeight:198 RetIndex:1706
CompName:Naphthalene, 1,6-dimethyl-4-(1-methylethyl)- $$ Naphthalene, 4-isopropyl-1,6-dimethy

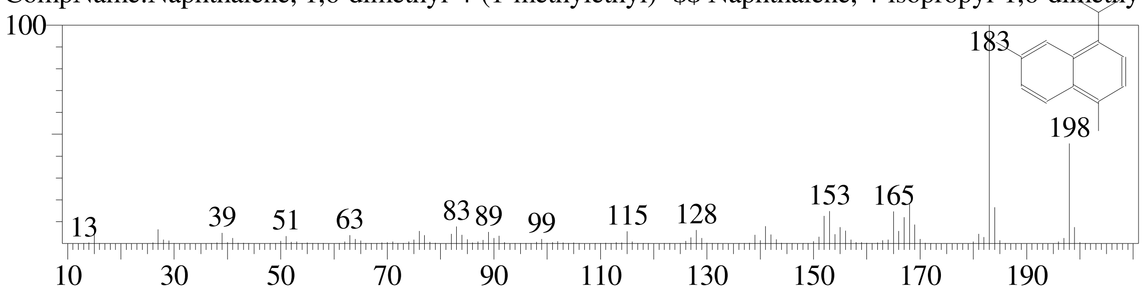

Hit#:3 Entry:93767 Library:WILEY7.LIB
SI:86 Formula:C15 H18 CAS:483-78-3 MolWeight:198 RetIndex:0
CompName:Naphthalene, 1,6-dimethyl-4-(1-methylethyl)- (CAS) Cadalin $$ 1,6-DIMETHYL-4-IS

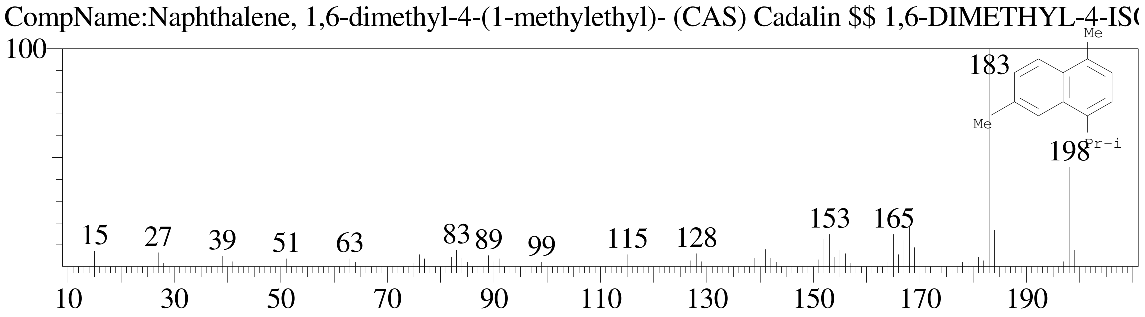

<< Target >>
Line#:7   R.Time:22.463(Scan#:4312) MassPeaks:27
RawMode:Averaged 22.458-22.467(4311-4313) BasePeak:57.05(5590)
BG Mode:Calc. from Peak

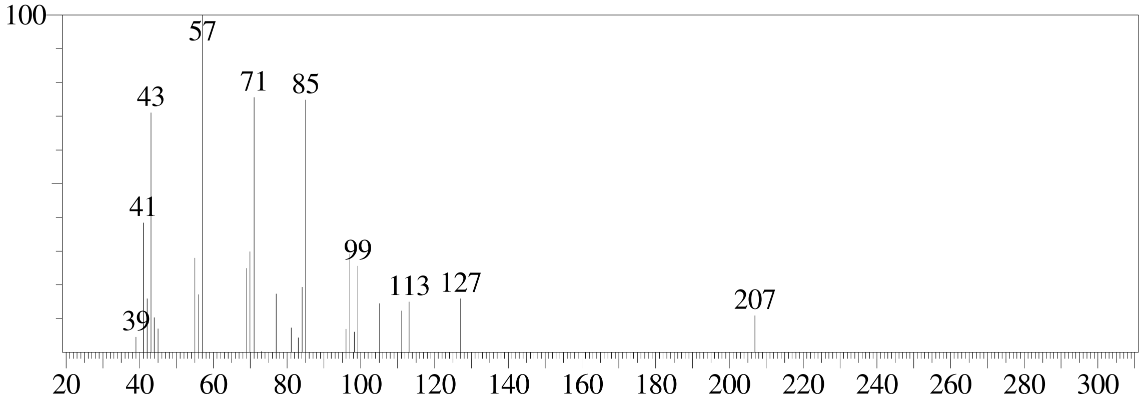

Hit#:1 Entry:24149 Library:NIST11s.lib
SI:85 Formula:C19H40 CAS:629-92-5 MolWeight:268 RetIndex:1910
CompName:Nonadecane $$ n-Nonadecane $$

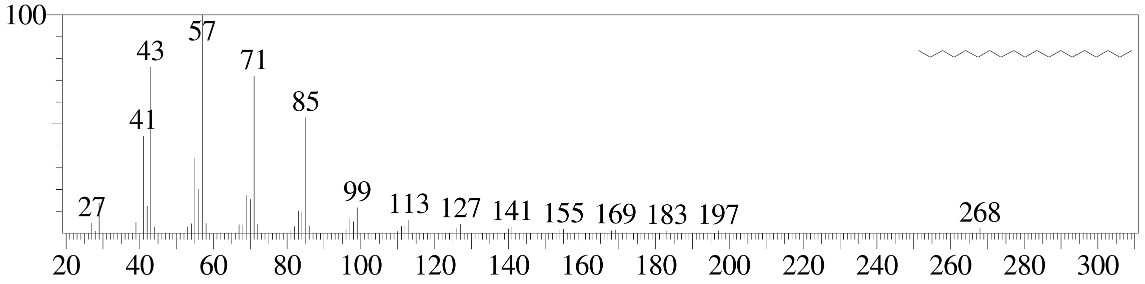

Hit#:2 Entry:139959 Library:NIST11.lib
SI:84 Formula:C19H36O4 CAS:0-00-0 MolWeight:328 RetIndex:2116
CompName:Oxalic acid, 6-ethyloct-3-yl heptyl ester

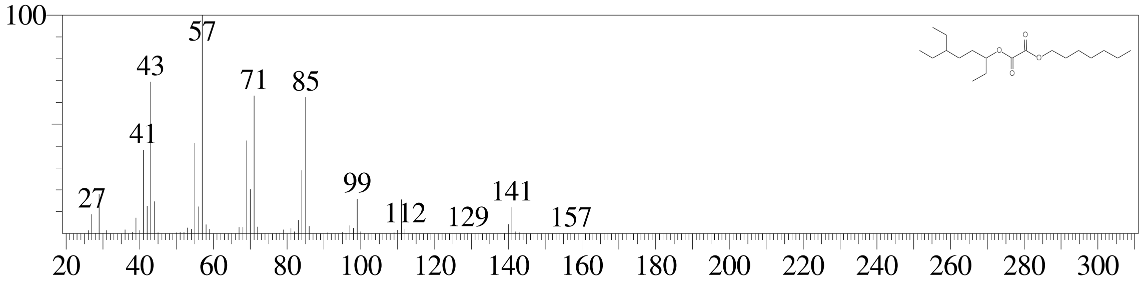

Hit#:3 Entry:221426 Library:WILEY7.LIB
SI:84 Formula:C22 H46 CAS:629-97-0 MolWeight:310 RetIndex:0
CompName:Docosane (CAS) n-Docosane $$ C22H46 STANDARD $$ Normal-docosane $$

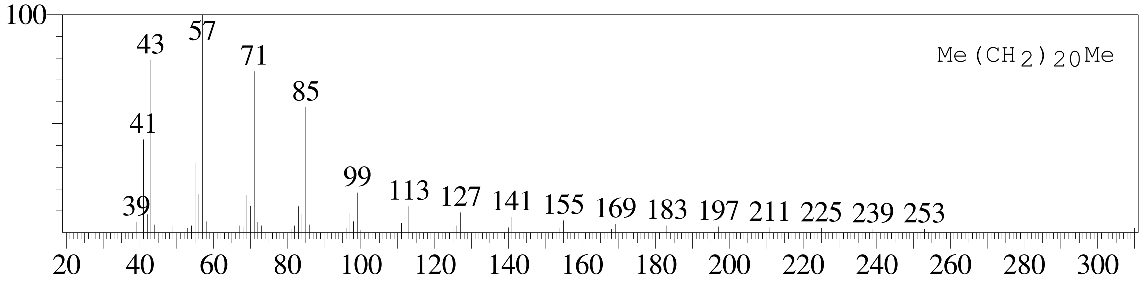

<< Target >>
Line#:8   R.Time:22.513(Scan#:4324) MassPeaks:29
RawMode:Averaged 22.508-22.517(4323-4325) BasePeak:57.05(10132)
BG Mode:Calc. from Peak

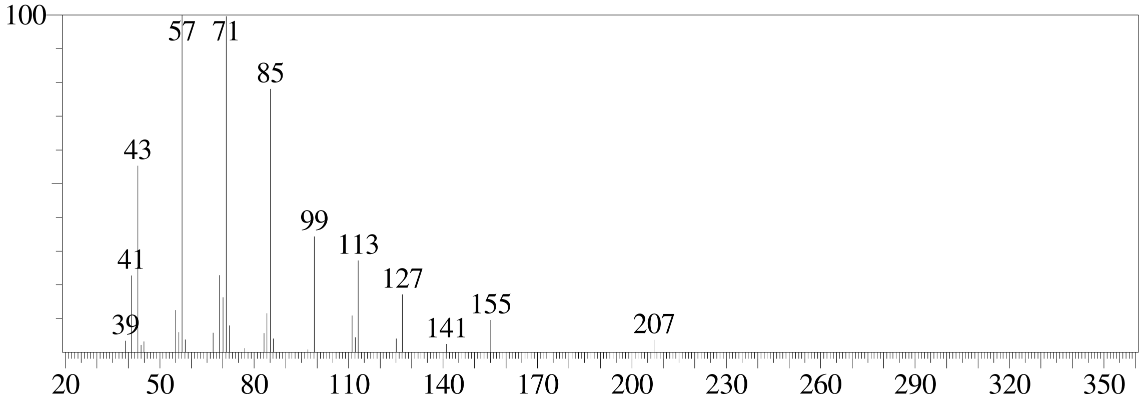

Hit#:1 Entry:73943 Library:NIST11.lib
SI:88 Formula:C16H34O CAS:0-00-0 MolWeight:242 RetIndex:1770
CompName:Tridecanol, 2-ethyl-2-methyl- $$ 2-Ethyl-2-methyl-1-tridecanol # $$

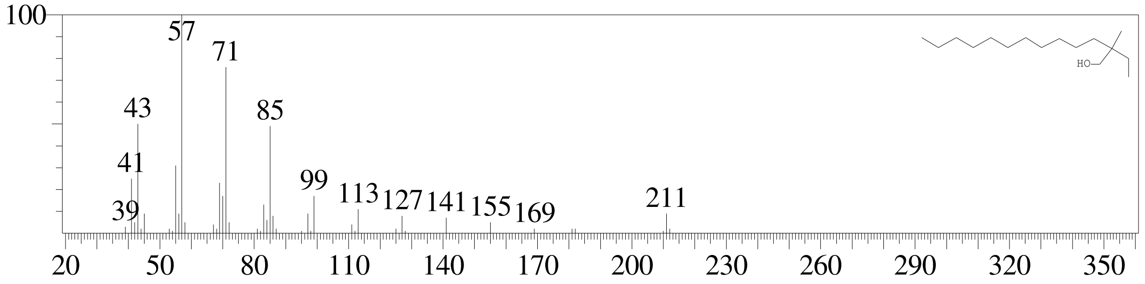

Hit#:2 Entry:156539 Library:NIST11.lib
SI:87 Formula:C16H33I CAS:544-77-4 MolWeight:352 RetIndex:2026
CompName:Hexadecane, 1-iodo- $$ Cetyl iodide $$ Hexadecyl iodide $$ 1-Iodohexadecane $$ n-H

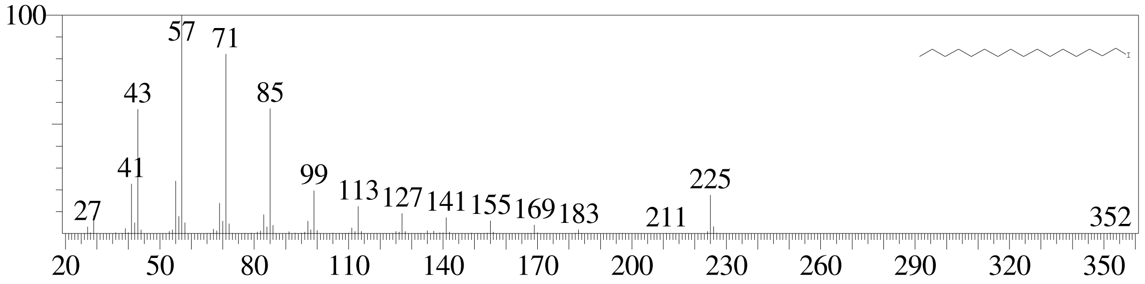

Hit#:3 Entry:17325 Library:NIST11s.lib
SI:87 Formula:C14H30 CAS:61141-72-8 MolWeight:198 RetIndex:1285
CompName:Dodecane, 4,6-dimethyl- $$ 4,6-Dimethyldodecane # $$

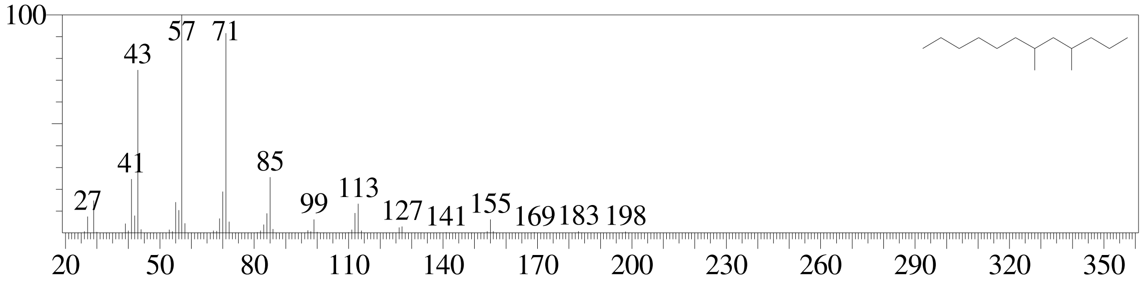

<< Target >>
Line#:9   R.Time:22.546(Scan#:4332) MassPeaks:31
RawMode:Averaged 22.542-22.550(4331-4333) BasePeak:57.05(14643)
BG Mode:Calc. from Peak

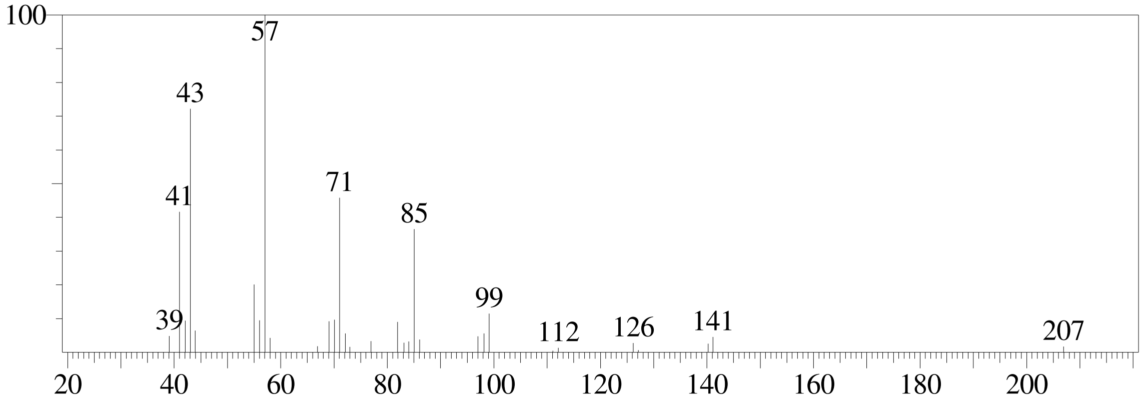

Hit#:1 Entry:19170 Library:NIST11s.lib
SI:94 Formula:C15H32 CAS:629-62-9 MolWeight:212 RetIndex:1512
CompName:Pentadecane $$ n-Pentadecane $$ CH3(CH2)13CH3 $$

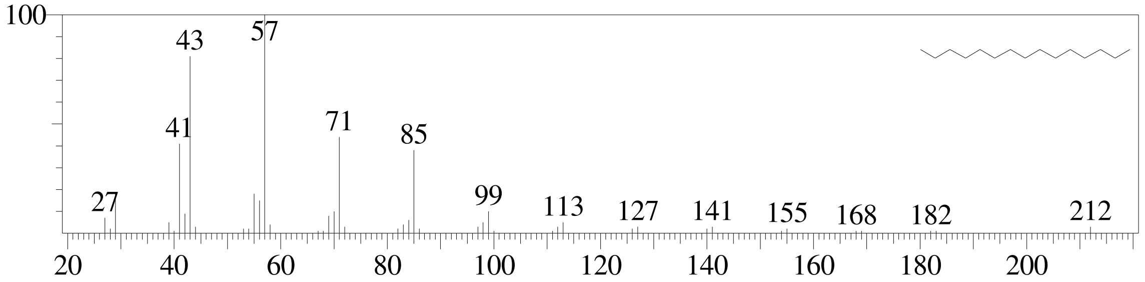

Hit#:2 Entry:111404 Library:WILEY7.LIB
SI:94 Formula:C15 H32 CAS:629-62-9 MolWeight:212 RetIndex:0
CompName:Pentadecane (CAS) n-Pentadecane $$ CH3(CH2)13CH3 $$

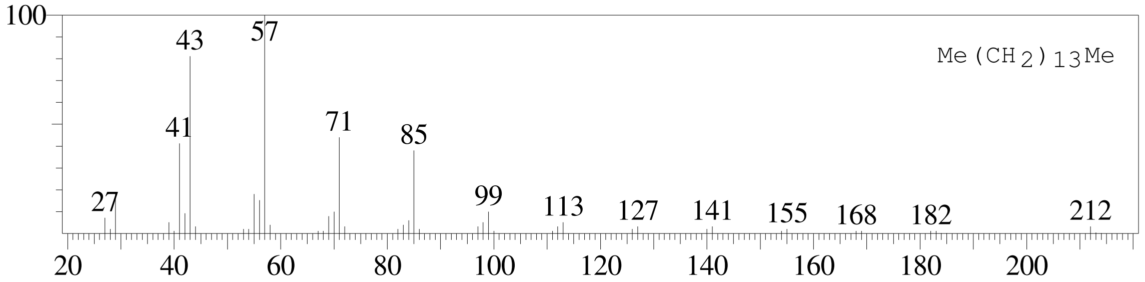

Hit#:3 Entry:755 Library:FFNSC1.3.lib
SI:93 Formula:C13 H28 CAS:629-50-5 MolWeight:184 RetIndex:1300
CompName:Tridecane <n->

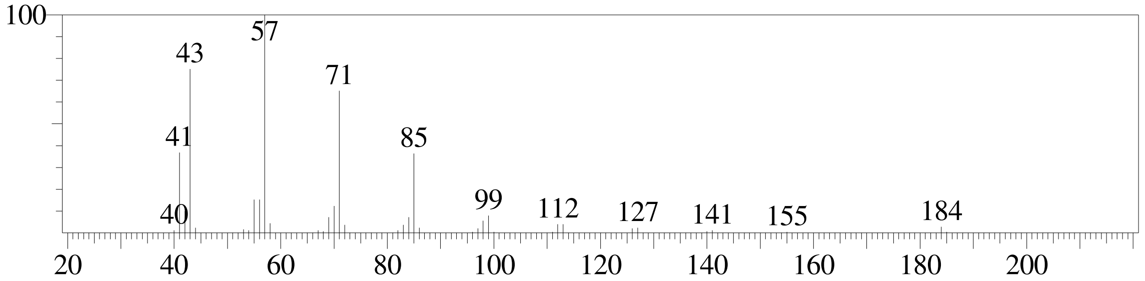

<< Target >>
Line#:10   R.Time:22.588(Scan#:4342) MassPeaks:28
RawMode:Averaged 22.583-22.592(4341-4343) BasePeak:57.05(4299)
BG Mode:Calc. from Peak

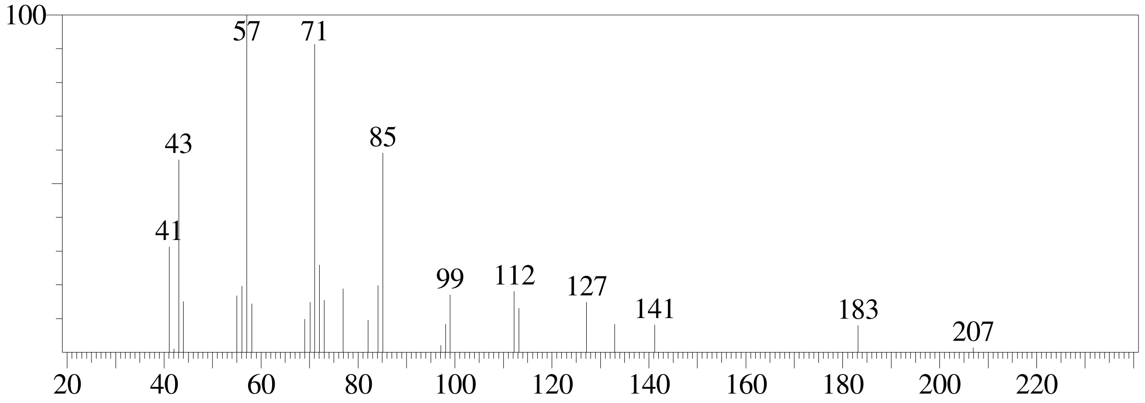

Hit#:1 Entry:82865 Library:NIST11.lib
SI:85 Formula:C18H38 CAS:20959-33-5 MolWeight:254 RetIndex:1746
CompName:Heptadecane, 7-methyl- $$ 7-Methylheptadecane $$

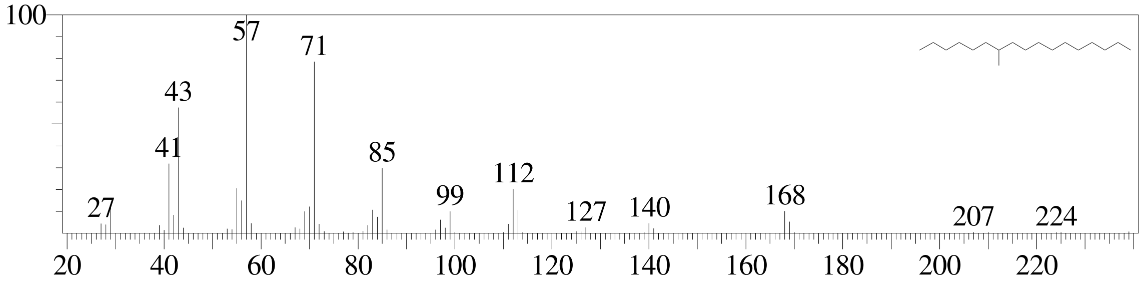

Hit#:2 Entry:17325 Library:NIST11s.lib
SI:85 Formula:C14H30 CAS:61141-72-8 MolWeight:198 RetIndex:1285
CompName:Dodecane, 4,6-dimethyl- $$ 4,6-Dimethyldodecane # $$

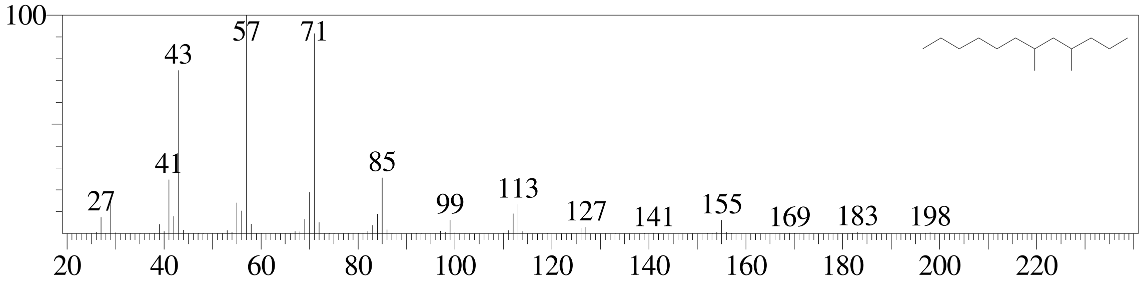

Hit#:3 Entry:755 Library:FFNSC1.3.lib
SI:85 Formula:C13 H28 CAS:629-50-5 MolWeight:184 RetIndex:1300
CompName:Tridecane <n->

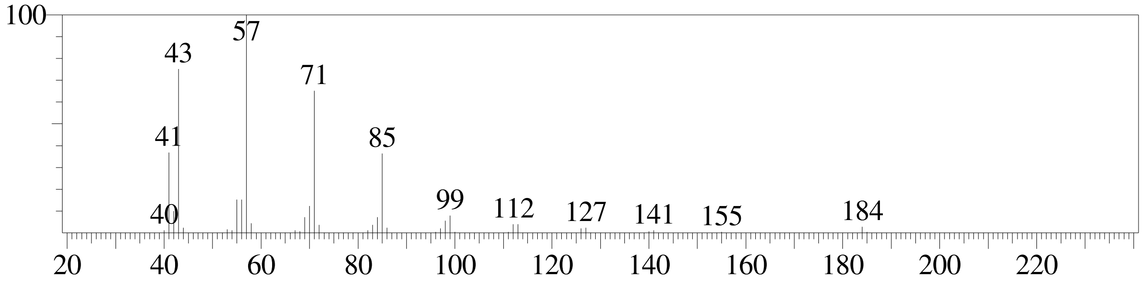

<< Target >>
Line#:11 R.Time:22.767(Scan#:4385) MassPeaks:19
RawMode:Averaged 22.763-22.771(4384-4386) BasePeak:57.05(16139)
BG Mode:Calc. from Peak

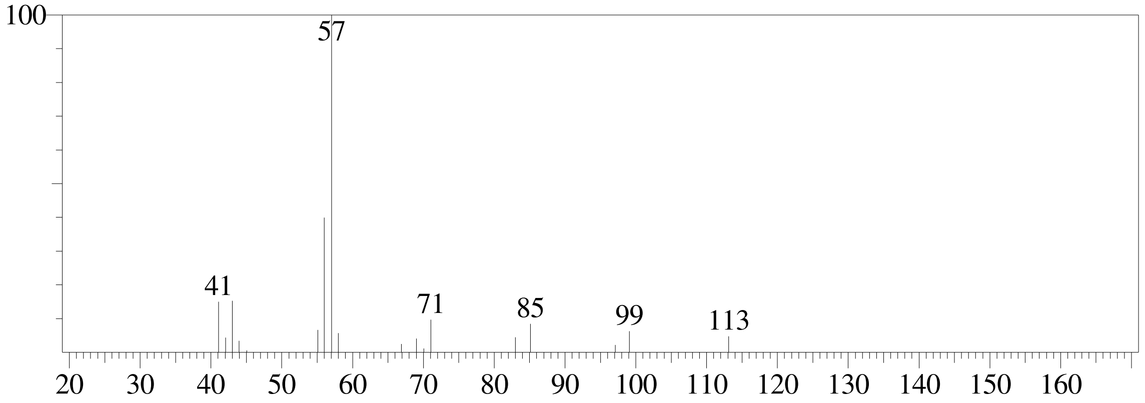

Hit#:1 Entry:61373 Library:WILEY7.LIB
SI:94 Formula:C12 H26 CAS:13475-82-6 MolWeight:170 RetIndex:0
CompName:Heptane, 2,2,4,6,6-pentamethyl- (CAS) 2,2,4,6,6-Pentamethylheptane $$

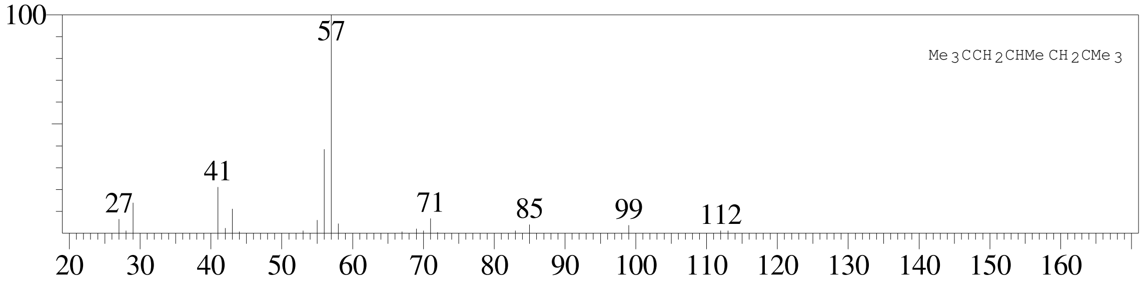

Hit#:2 Entry:61362 Library:WILEY7.LIB
SI:94 Formula:C12 H26 CAS:17302-37-3 MolWeight:170 RetIndex:0
CompName:Decane, 2,2-dimethyl- (CAS) 2,2-Dimethyldecane $$

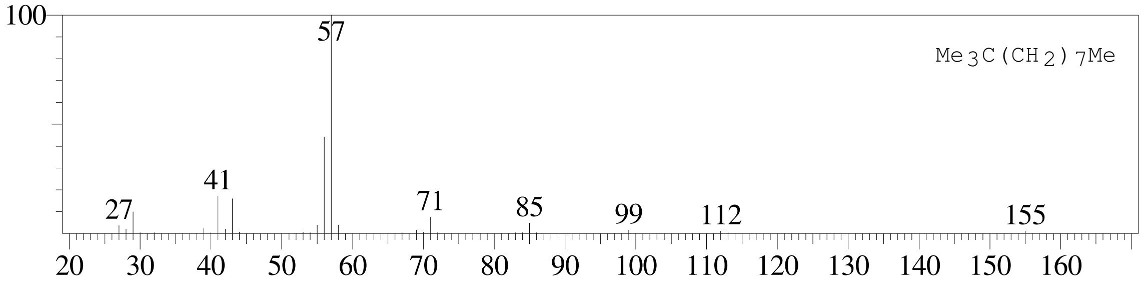

Hit#:3 Entry:26113 Library:NIST11.lib
SI:94 Formula:C12H26 CAS:17302-37-3 MolWeight:170 RetIndex:1130
CompName:Decane, 2,2-dimethyl- $$ 2,2-Dimethyldecane $$

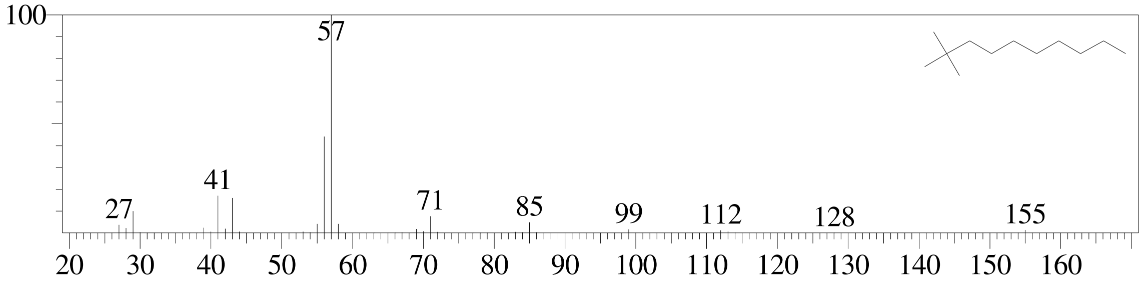

<< Target >>
Line#:12   R.Time:22.833(Scan#:4401) MassPeaks:31
RawMode:Averaged 22.829-22.838(4400-4402) BasePeak:57.05(6560)
BG Mode:Calc. from Peak

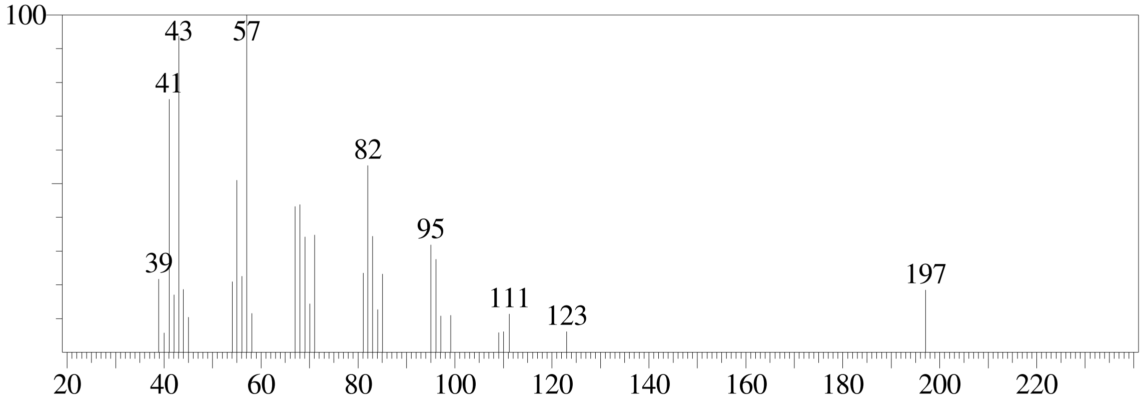

Hit#:1 Entry:22025 Library:NIST11s.lib
SI:91 Formula:C16H32O CAS:629-80-1 MolWeight:240 RetIndex:1800
CompName:Hexadecanal $$ Palmitaldehyde $$ 1-Hexadecanal $$ n-Hexadecanal $$

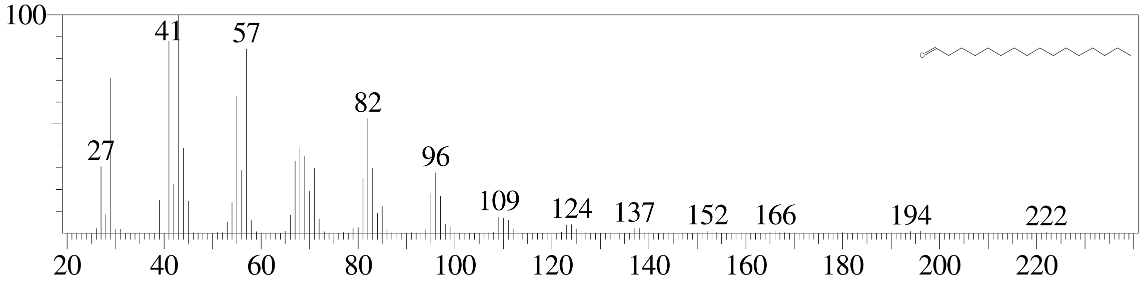

Hit#:2 Entry:111366 Library:WILEY7.LIB
SI:90 Formula:C14 H28 O CAS:124-25-4 MolWeight:212 RetIndex:0
CompName:Tetradecanal (CAS) Myristaldehyde $$ Myristylaldehyde $$ Tetradecylaldehyde $$ n-T

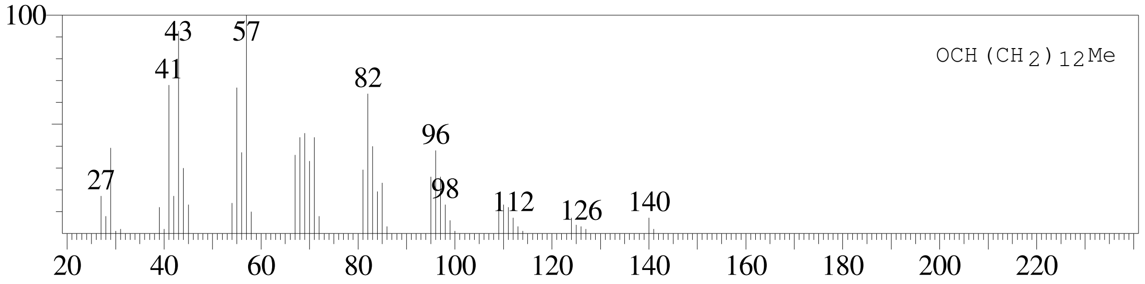

Hit#:3 Entry:15112 Library:NIST11s.lib
SI:90 Formula:C12H24O CAS:112-54-9 MolWeight:184 RetIndex:1402
CompName:Dodecanal $$ Lauraldehyde $$ n-Dodecanal $$ n-Dodecyl aldehyde $$ n-Dodecylic alc

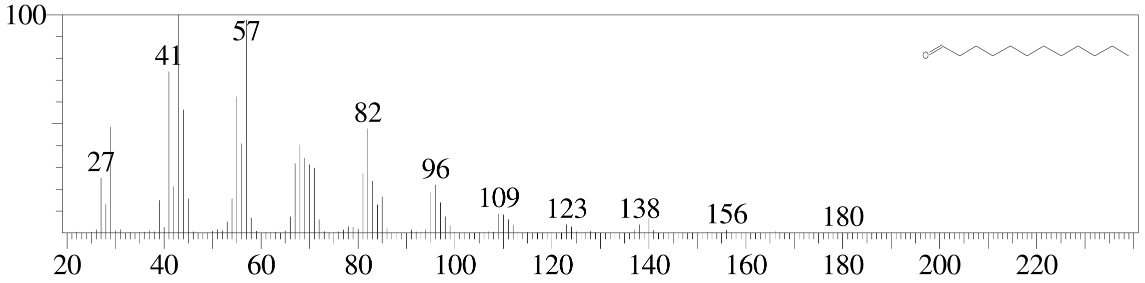

<< Target >>
Line#:13 R.Time:23.579(Scan#:4580) MassPeaks:74
RawMode:Averaged 23.575-23.583(4579-4581) BasePeak:105.00(1045006)
BG Mode:Calc. from Peak

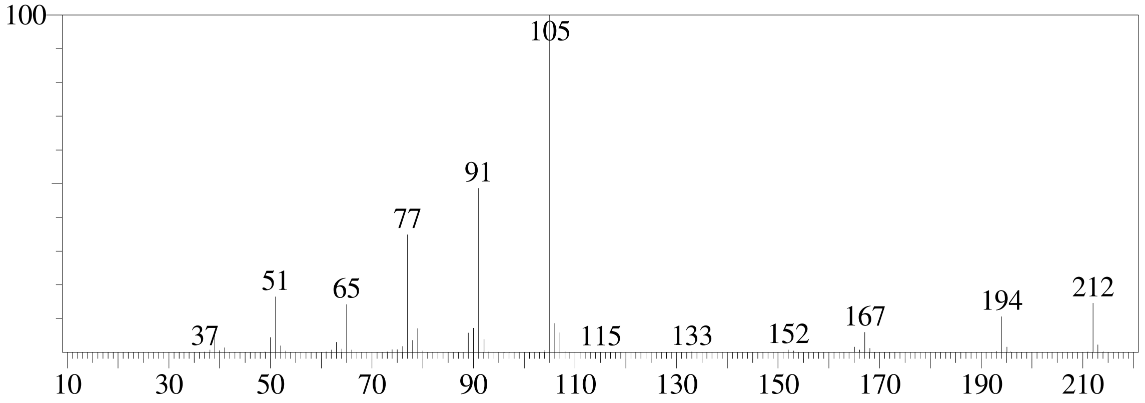

Hit#:1 Entry:19121 Library:NIST11s.lib
SI:98 Formula:C14H12O2 CAS:120-51-4 MolWeight:212 RetIndex:1733
CompName:Benzyl Benzoate $$ Benzoic acid, phenylmethyl ester $$ Benzoic acid, benzyl ester $$.

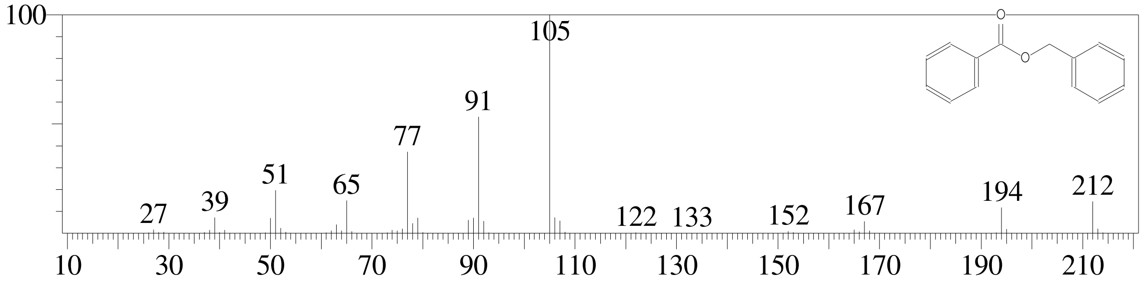

Hit#:2 Entry:111318 Library:WILEY7.LIB
SI:98 Formula:C14 H12 O2 CAS:120-51-4 MolWeight:212 RetIndex:0
CompName:Benzyl benzoate $$ Benzoic acid, phenylmethyl ester (CAS) BENZYL ESTER OF BEN

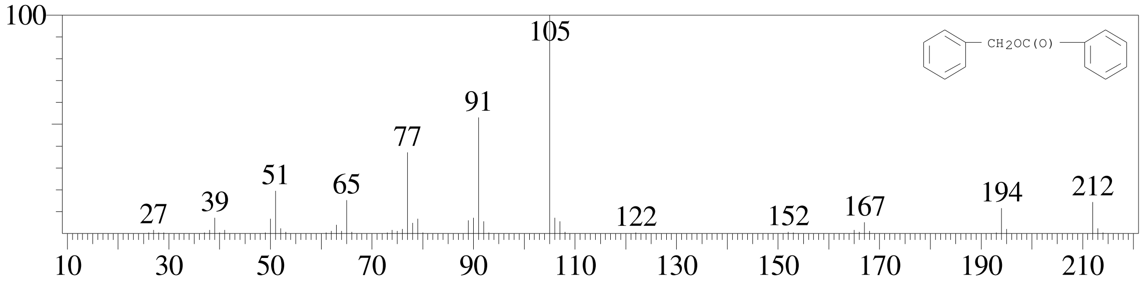

Hit#:3 Entry:52340 Library:NIST11.lib
SI:98 Formula:C14H12O2 CAS:120-51-4 MolWeight:212 RetIndex:1733
CompName:Benzyl Benzoate $$ Benzoic acid, phenylmethyl ester $$ Benzoic acid, benzyl ester $$.

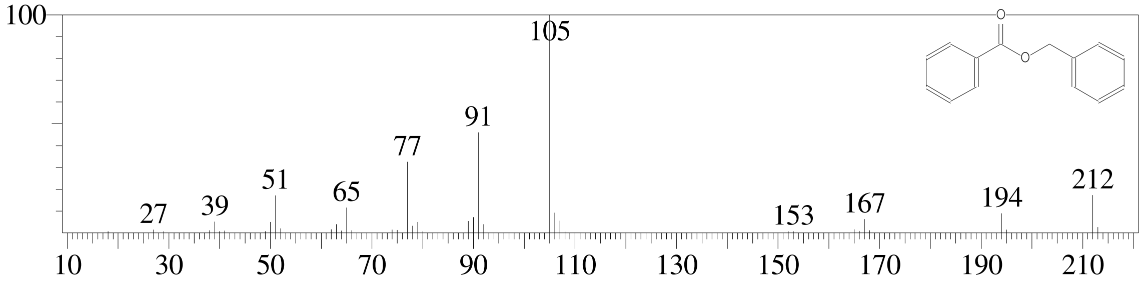

<< Target >>
Line#:14 R.Time:23.908(Scan#:4659) MassPeaks:61
RawMode:Averaged 23.904-23.913(4658-4660) BasePeak:57.05(91413)
BG Mode:Calc. from Peak

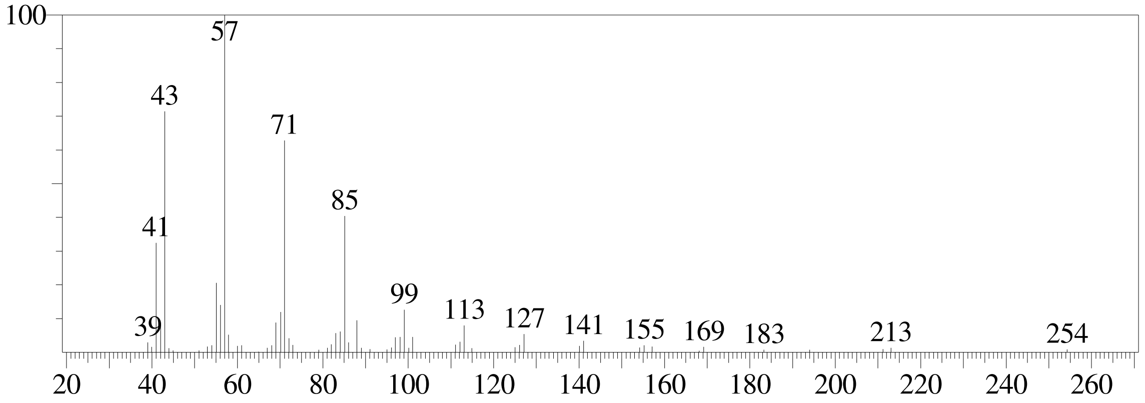

Hit#:1 Entry:111398 Library:WILEY7.LIB
SI:95 Formula:C15 H32 CAS:629-62-9 MolWeight:212 RetIndex:0
CompName:Pentadecane (CAS) n-Pentadecane $$ CH3(CH2)13CH3 $$

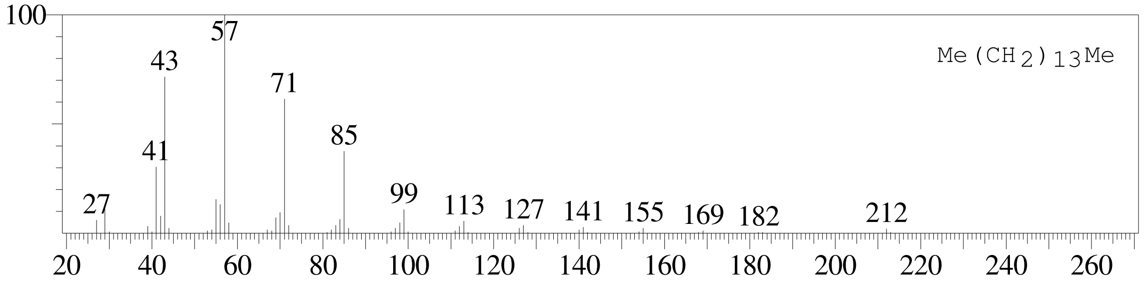

Hit#:2 Entry:815 Library:FFNSC1.3.lib
SI:95 Formula:C15 H32 CAS:629-62-9 MolWeight:212 RetIndex:1500
CompName:Pentadecane <n->

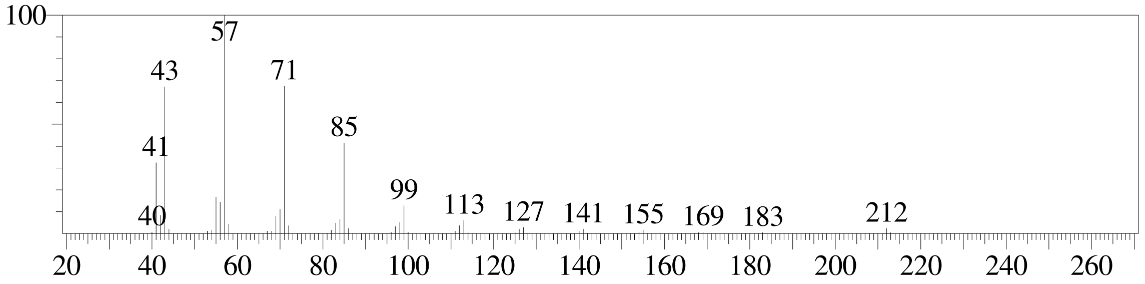

Hit#:3 Entry:178176 Library:WILEY7.LIB
SI:94 Formula:C19 H40 CAS:629-92-5 MolWeight:268 RetIndex:0
CompName:Nonadecane (CAS) n-Nonadecane $$

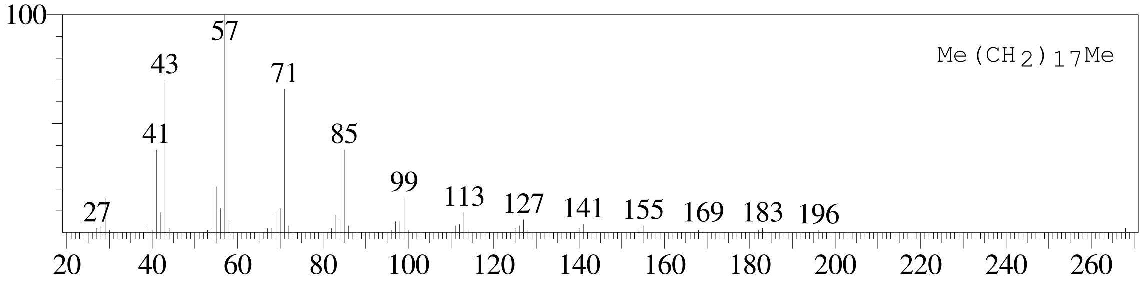

<< Target >>
Line#:15   R.Time:24.279(Scan#:4748) MassPeaks:53
RawMode:Averaged 24.275-24.283(4747-4749) BasePeak:43.00(19206)
BG Mode:Calc. from Peak

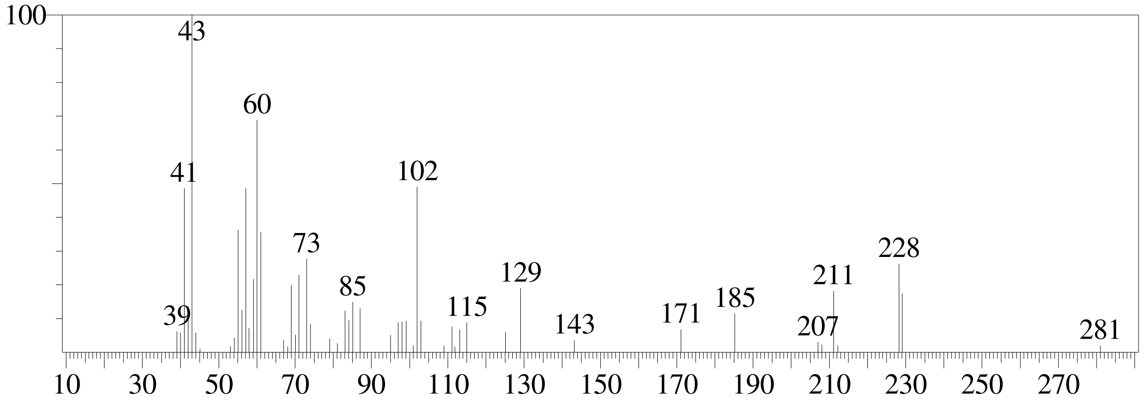

Hit#:1 Entry:95171 Library:NIST11.lib
SI:94 Formula:C17H34O2 CAS:110-27-0 MolWeight:270 RetIndex:1814
CompName:Isopropyl myristate $$ Tetradecanoic acid, 1-methylethyl ester $$ Myristic acid, isoprop

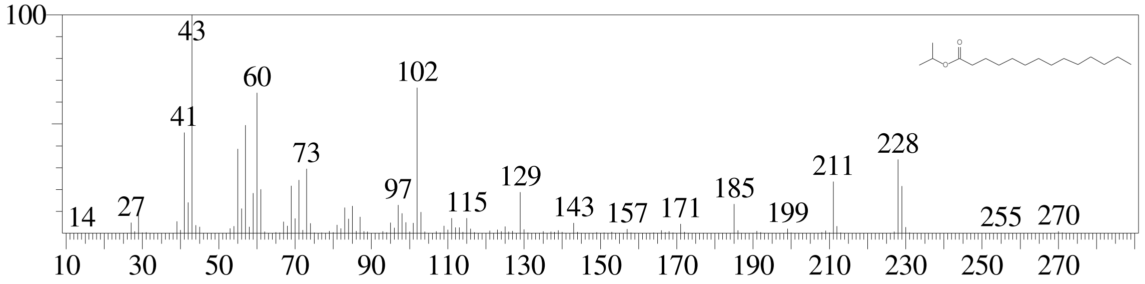

Hit#:2 Entry:370 Library:FFNSC1.3.lib
SI:94 Formula:C17 H34 O2 CAS:110-27-0 MolWeight:270 RetIndex:1826
CompName:Tetradecanoate <isopropyl->

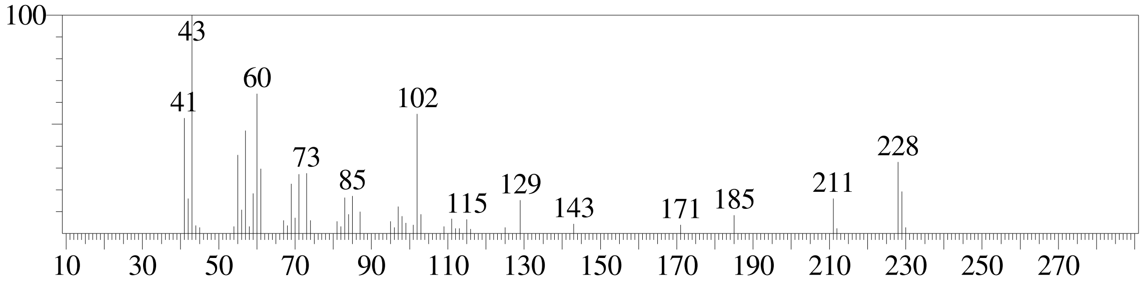

Hit#:3 Entry:180480 Library:WILEY7.LIB
SI:93 Formula:C17 H34 O2 CAS:110-27-0 MolWeight:270 RetIndex:0
CompName:Isopropyl myristate $$ Tetradecanoic acid, 1-methylethyl ester $$ D 50 $$ ISOPROPYI

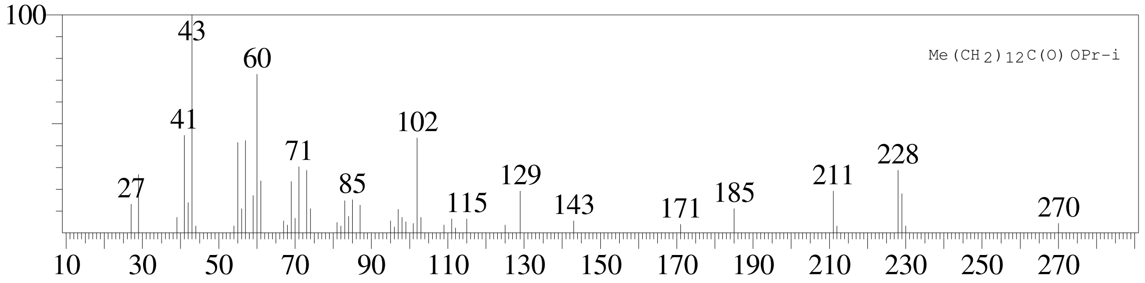

<< Target >>
Line#:16   R.Time:24.788(Scan#:4870) MassPeaks:42
RawMode:Averaged 24.783-24.792(4869-4871) BasePeak:149.00(116636)
BG Mode:Calc. from Peak

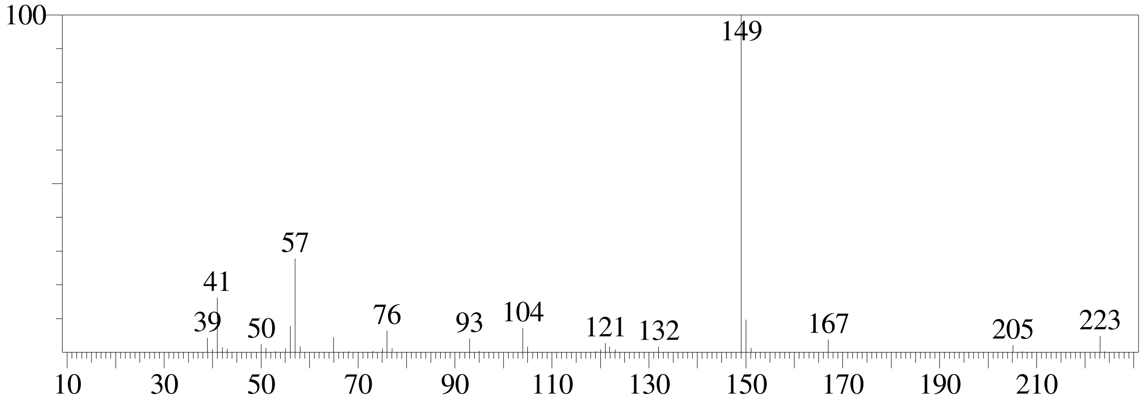

Hit#:1 Entry:24792 Library:NIST11s.lib
SI:95 Formula:C16H22O4 CAS:84-69-5 MolWeight:278 RetIndex:1908
CompName:1,2-Benzenedicarboxylic acid, bis(2-methylpropyl) ester $$ Phthalic acid, diisobutyl est

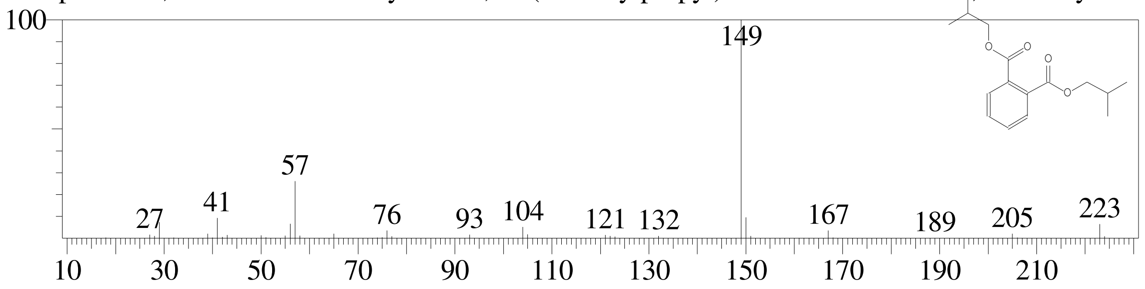

Hit#:2 Entry:188947 Library:WILEY7.LIB
SI:94 Formula:C16 H22 O4 CAS:84-69-5 MolWeight:278 RetIndex:0
CompName:1,2-Benzenedicarboxylic acid, bis(2-methylpropyl) ester (CAS) Isobutyl phthalate $$ IS

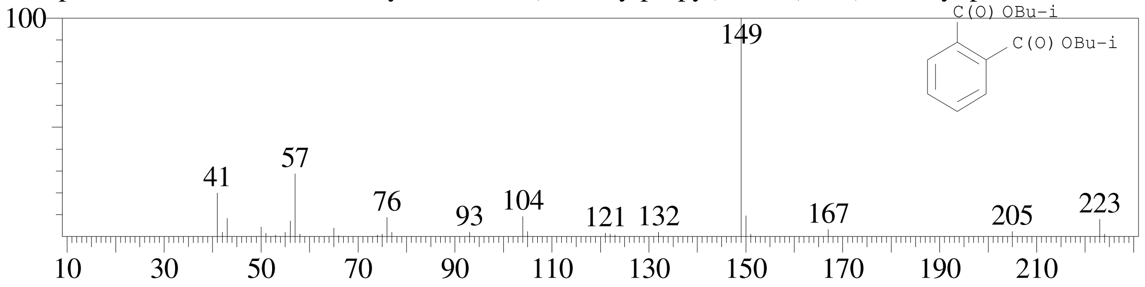

Hit#:3 Entry:188944 Library:WILEY7.LIB
SI:94 Formula:C16 H22 O4 CAS:84-69-5 MolWeight:278 RetIndex:0
CompName:1,2-Benzenedicarboxylic acid, bis(2-methylpropyl) ester (CAS) Isobutyl phthalate $$ IS

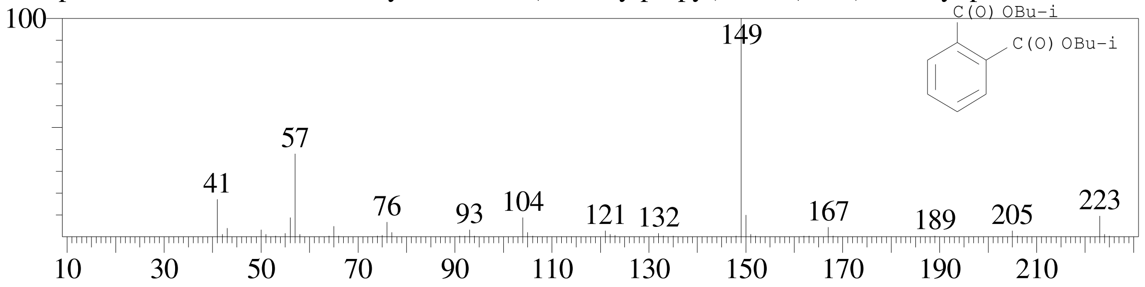

<< Target >>
Line#:17 R.Time:25.225(Scan#:4975) MassPeaks:38
RawMode:Averaged 25.221-25.229(4974-4976) BasePeak:57.05(16898)
BG Mode:Calc. from Peak

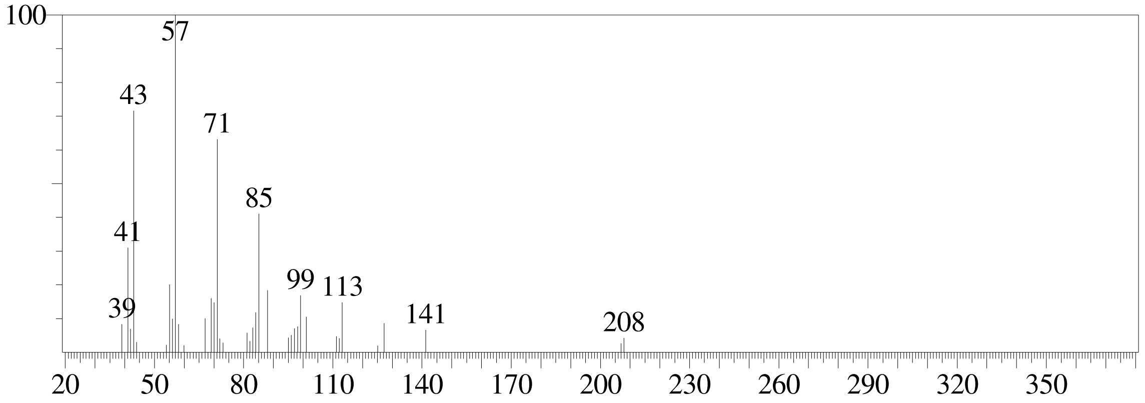

Hit#:1 Entry:273896 Library:WILEY7.LIB
SI:89 Formula:C27 H56 CAS:593-49-7 MolWeight:380 RetIndex:0
CompName:Heptacosane (CAS) n-Heptacosane $$

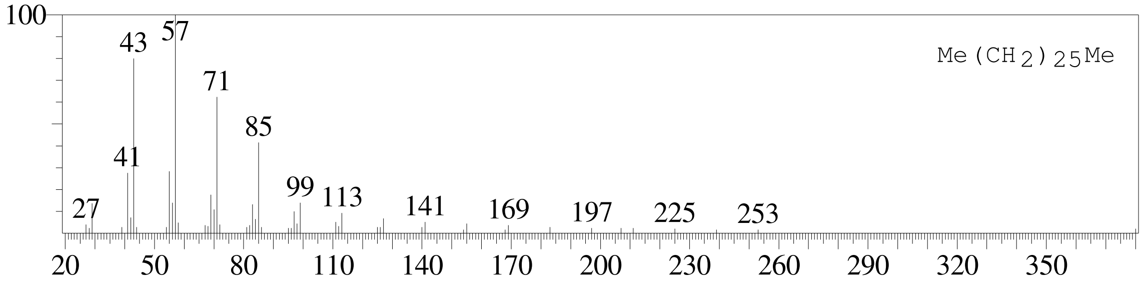

Hit#:2 Entry:245570 Library:WILEY7.LIB
SI:89 Formula:C24 H50 CAS:646-31-1 MolWeight:338 RetIndex:0
CompName:Tetracosane (CAS) n-Tetracosane $$

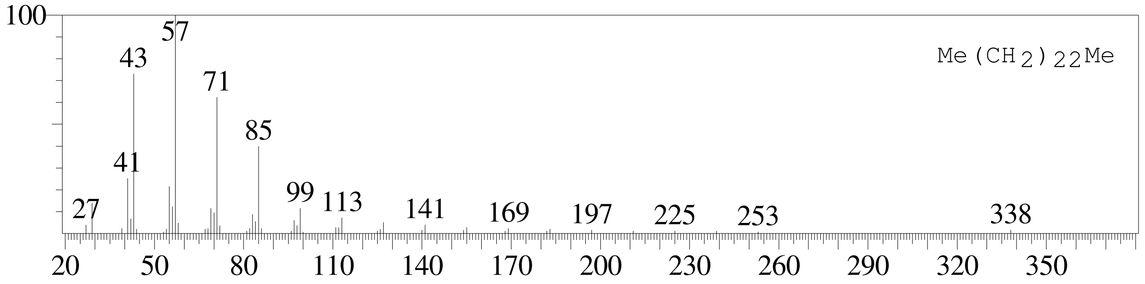

Hit#:3 Entry:221437 Library:WILEY7.LIB
SI:89 Formula:C22 H46 CAS:71005-15-7 MolWeight:310 RetIndex:0
CompName:PENTADECANE, 8-HEPTYL- $$

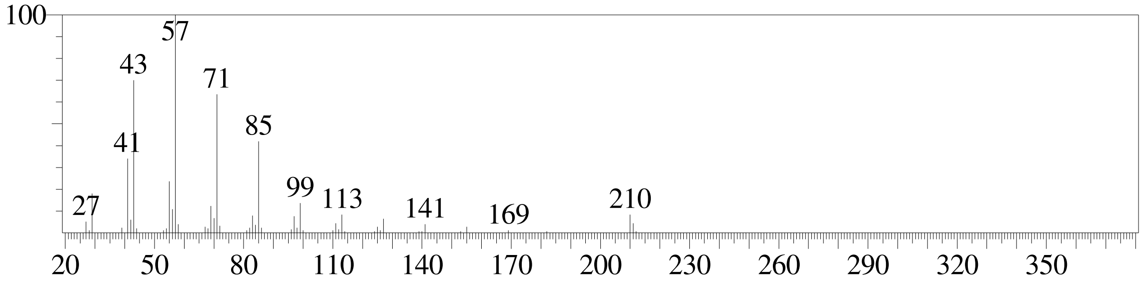

<< Target >>
Line#:18 R.Time:25.379(Scan#:5012) MassPeaks:43
RawMode:Averaged 25.375-25.383(5011-5013) BasePeak:105.05(15782)
BG Mode:Calc. from Peak

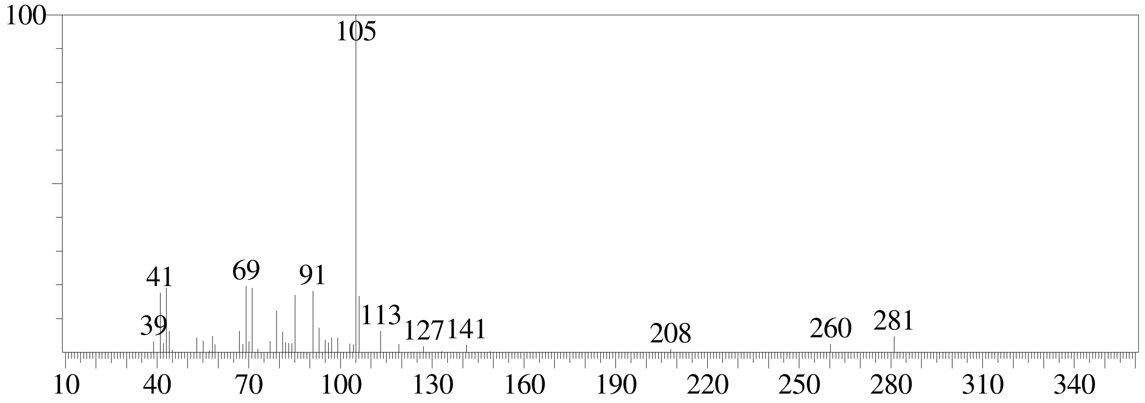

Hit#:1 Entry:260099 Library:WILEY7.LIB
SI:78 Formula:C26 H46 CAS:2398-66-5 MolWeight:358 RetIndex:0
CompName:Benzene, (1-methylnonadecyl)- (CAS) Eicosane, 2-phenyl- $$ 2-Phenyleicosane $$

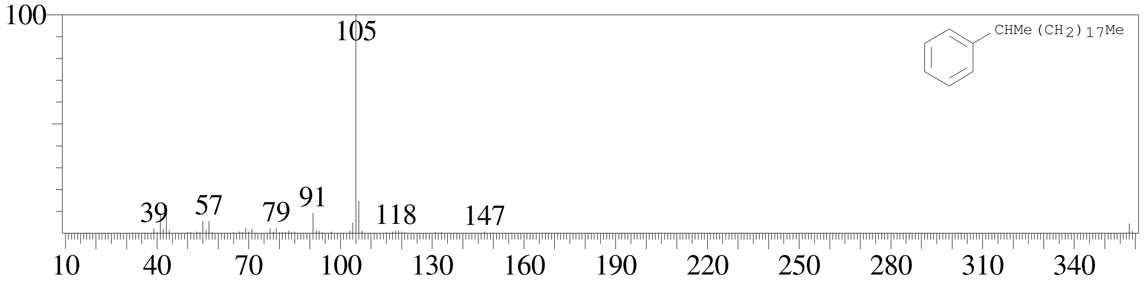

Hit#:2 Entry:152483 Library:WILEY7.LIB
SI:76 Formula:C18 H30 CAS:54986-44-6 MolWeight:246 RetIndex:0
CompName:Benzene, (1,3,3-trimethylnonyl)- (CAS) 2-PHENYL-4,4-DIMETHYLDECANE $$

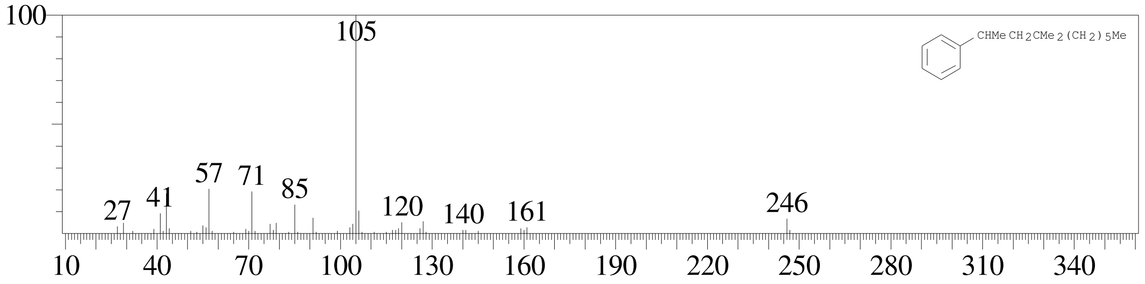

Hit#:3 Entry:76890 Library:NIST11.lib
SI:76 Formula:C18H30 CAS:54986-44-6 MolWeight:246 RetIndex:1738
CompName:Benzene, (1,3,3-trimethylnonyl)- $$ (1,3,3-Trimethylnonyl)benzene # $$

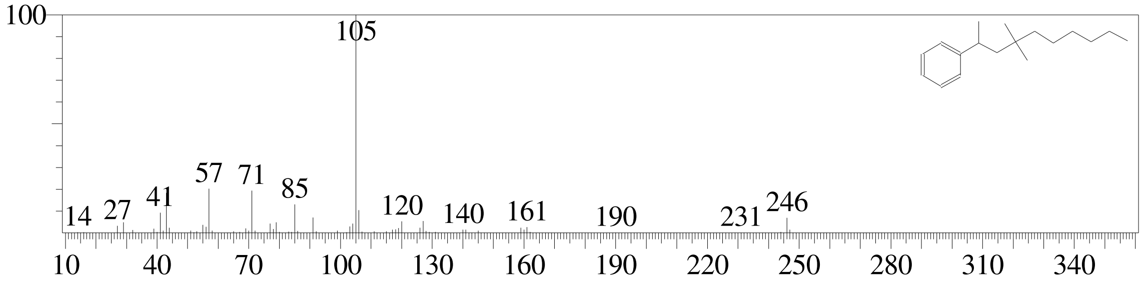

<< Target >>
Line#:19   R.Time:25.433(Scan#:5025) MassPeaks:33
RawMode:Averaged 25.429-25.438(5024-5026) BasePeak:57.05(27331)
BG Mode:Calc. from Peak

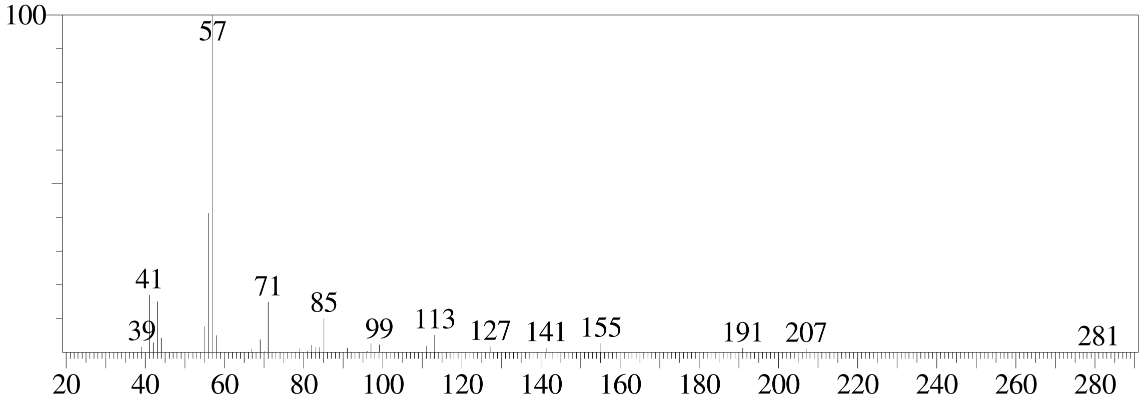

Hit#:1 Entry:128885 Library:WILEY7.LIB
SI:93 Formula:C16 H34 CAS:127204-12-0 MolWeight:226 RetIndex:0
CompName:DODECANE, 2,2,11,11-TETRAMETHYL- $$

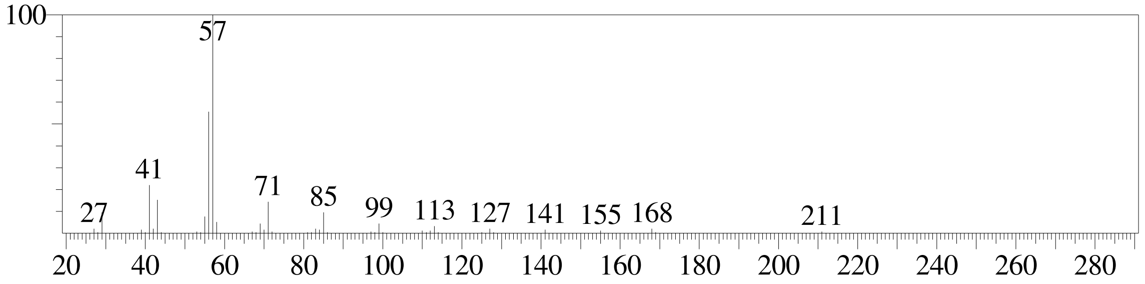

Hit#:2 Entry:62496 Library:NIST11.lib
SI:93 Formula:C16H34 CAS:127204-12-0 MolWeight:226 RetIndex:1443
CompName:Dodecane, 2,2,11,11-tetramethyl- $$ 2,2,11,11-Tetramethyldodecane # $$

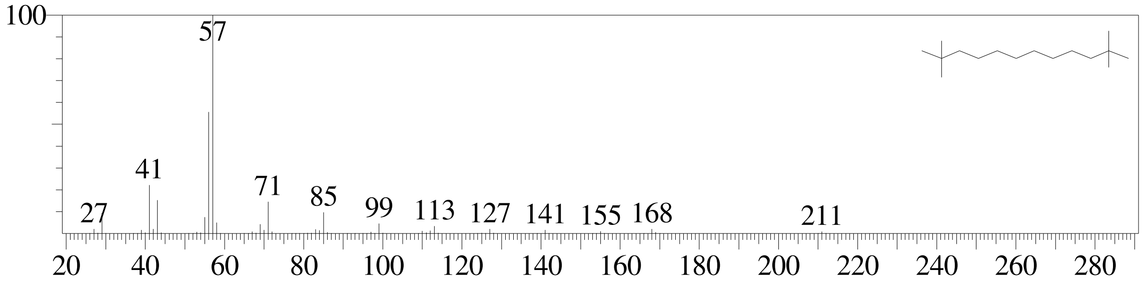

Hit#:3 Entry:62497 Library:NIST11.lib
SI:93 Formula:C16H34 CAS:59222-86-5 MolWeight:226 RetIndex:1527
CompName:Tetradecane, 2,2-dimethyl- $$ 2,2-Dimethyltetradecane # $$

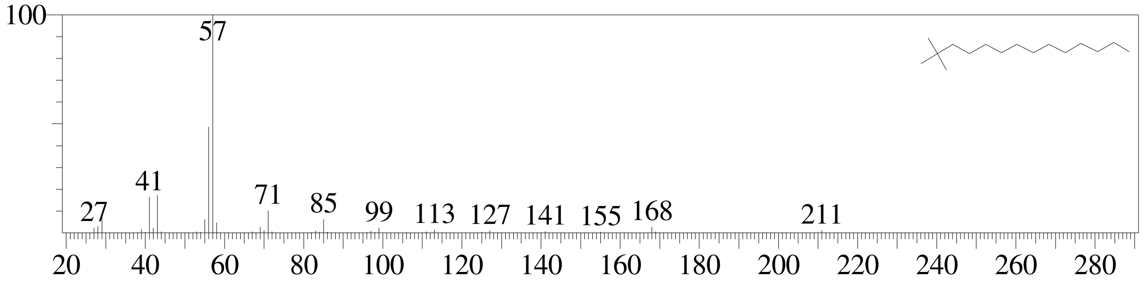

<< Target >>
Line#:20 R.Time:25.583(Scan#:5061) MassPeaks:59
RawMode:Averaged 25.579-25.588(5060-5062) BasePeak:74.05(55452)
BG Mode:Calc. from Peak

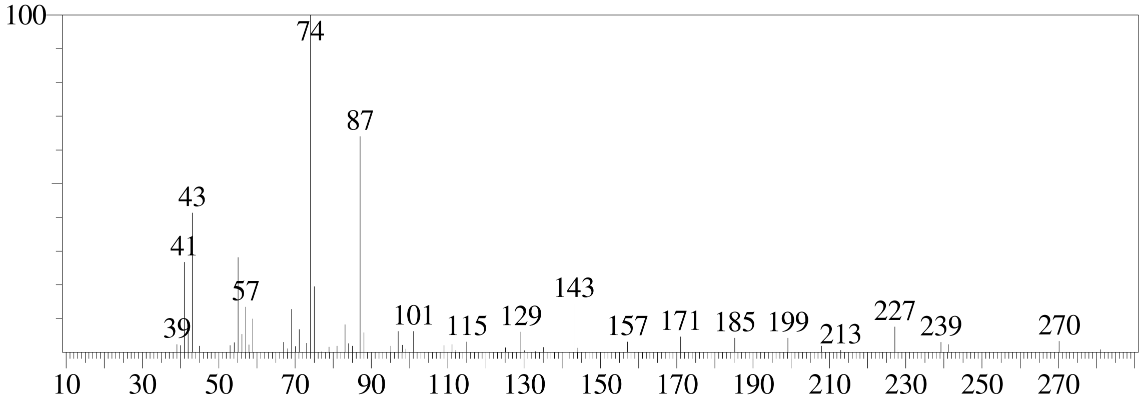

Hit#:1 Entry:180433 Library:WILEY7.LIB
SI:95 Formula:C17 H34 O2 CAS:112-39-0 MolWeight:270 RetIndex:0
CompName:Hexadecanoic acid, methyl ester (CAS) Methyl palmitate $$ Methyl hexadecanoate $$ [illegible]

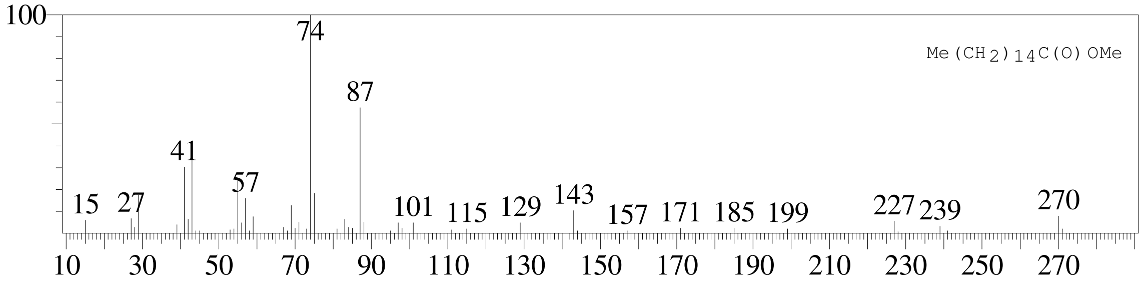

Hit#:2 Entry:24296 Library:NIST11s.lib
SI:95 Formula:C17H34O2 CAS:112-39-0 MolWeight:270 RetIndex:1878
CompName:Hexadecanoic acid, methyl ester $$ Palmitic acid, methyl ester $$ n-Hexadecanoic acid

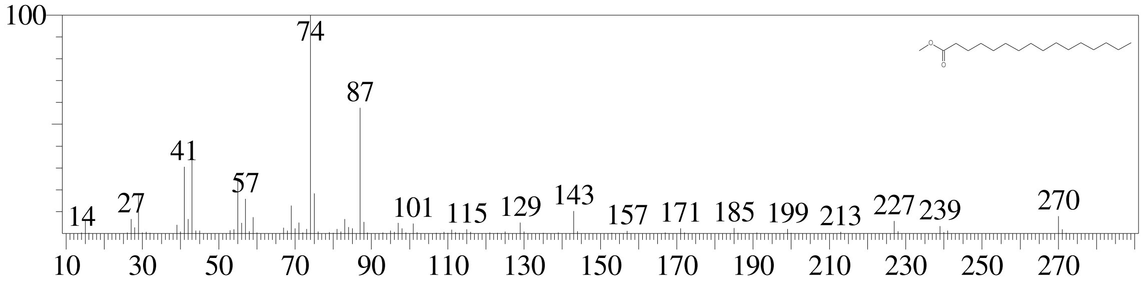

Hit#:3 Entry:180432 Library:WILEY7.LIB
SI:95 Formula:C17 H34 O2 CAS:112-39-0 MolWeight:270 RetIndex:0
CompName:Hexadecanoic acid, methyl ester (CAS) Methyl palmitate $$ Methyl hexadecanoate $$ [illegible]

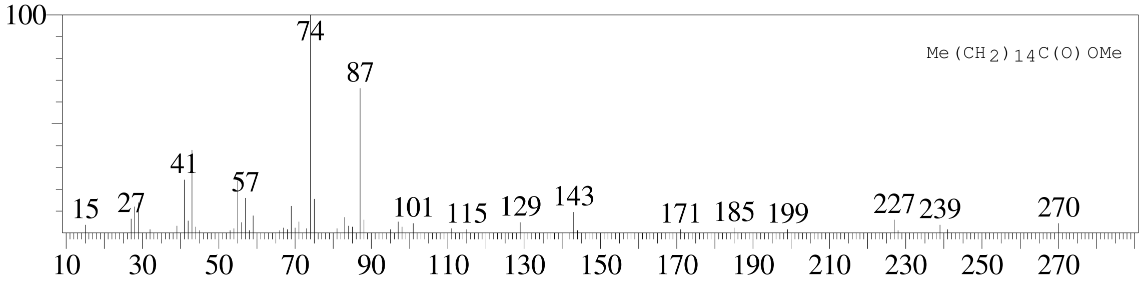

<< Target >>
Line#:21 R.Time:26.117(Scan#:5189) MassPeaks:59
RawMode:Averaged 26.113-26.121(5188-5190) BasePeak:149.00(21838)
BG Mode:Calc. from Peak

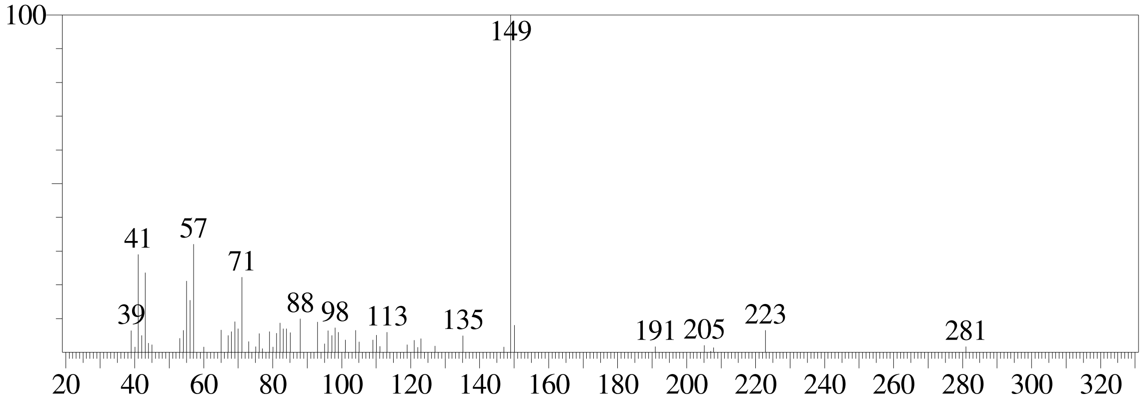

Hit#:1 Entry:171436 Library:NIST11.lib
SI:81 Formula:C23H36O4 CAS:0-00-0 MolWeight:376 RetIndex:2732
CompName:Phthalic acid, butyl undecyl ester

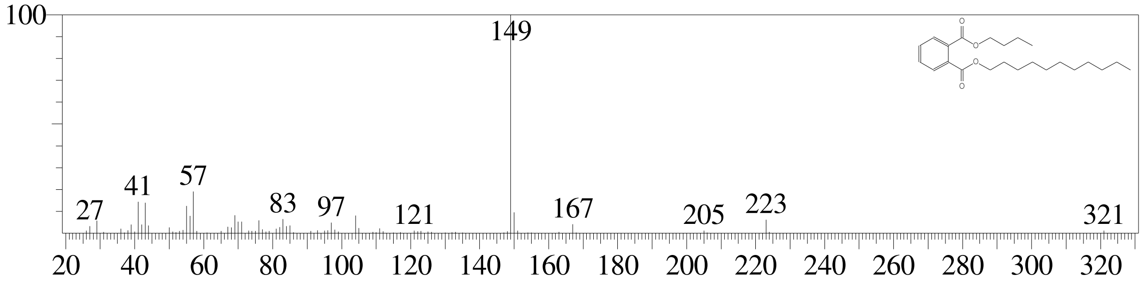

Hit#:2 Entry:292961 Library:WILEY7.LIB
SI:80 Formula:C26 H42 O4 CAS:28553-12-0 MolWeight:418 RetIndex:0
CompName:1,2-Benzenedicarboxylic acid, diisononyl ester $$ Phthalic acid, diisononyl ester $$ Dii

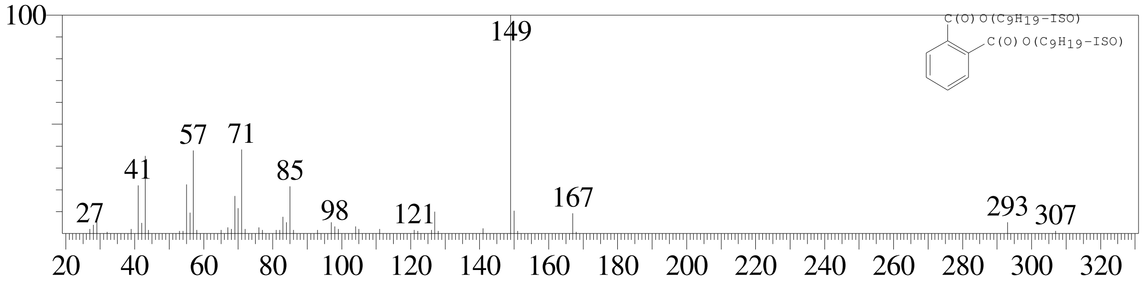

Hit#:3 Entry:29981 Library:NIST11s.lib
SI:80 Formula:C26H42O4 CAS:20548-62-3 MolWeight:418 RetIndex:2902
CompName:Phthalic acid, bis(7-methyloctyl) ester $$ 1,2-Benzenedicarboxylic acid, bis(7-methyloc

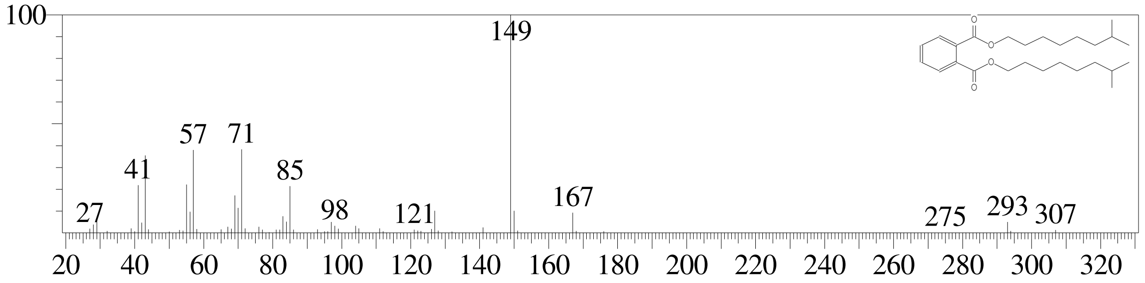

<< Target >>
Line#:22 R.Time:26.413(Scan#:5260) MassPeaks:90
RawMode:Averaged 26.408-26.417(5259-5261) BasePeak:88.05(54261)
BG Mode:Calc. from Peak

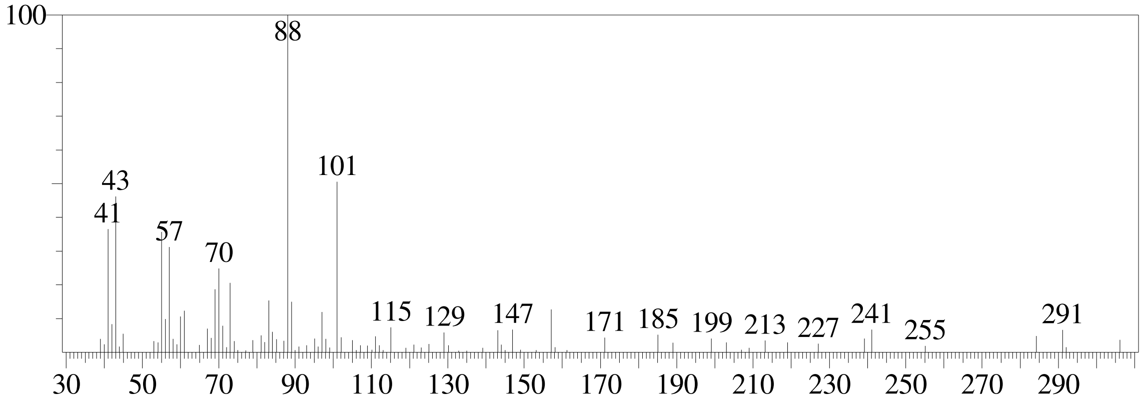

Hit#:1 Entry:195615 Library:WILEY7.LIB
SI:92 Formula:C18 H36 O2 CAS:628-97-7 MolWeight:284 RetIndex:0
CompName:Hexadecanoic acid, ethyl ester (CAS) Ethyl palmitate $$ HEXADECANOIC ACID ET

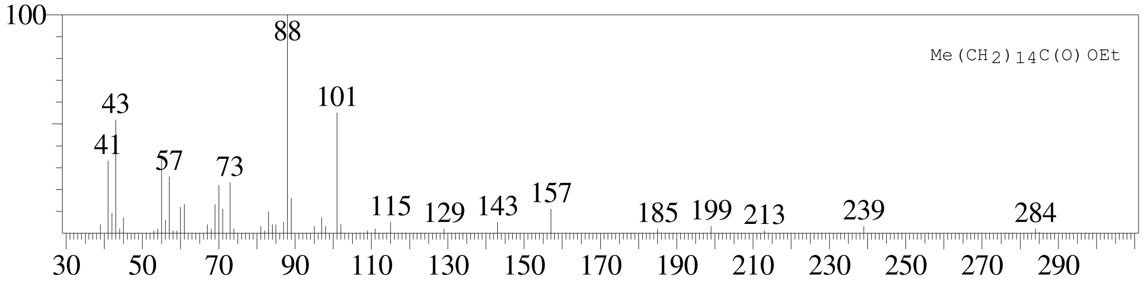

Hit#:2 Entry:374 Library:FFNSC1.3.lib
SI:91 Formula:C18 H36 O2 CAS:628-97-7 MolWeight:284 RetIndex:1993
CompName:Palmitate <ethyl->

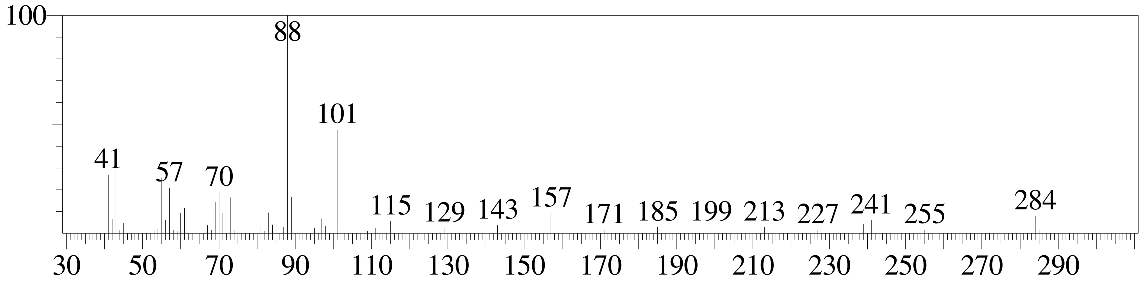

Hit#:3 Entry:180467 Library:WILEY7.LIB
SI:91 Formula:C17 H34 O2 CAS:41114-00-5 MolWeight:270 RetIndex:0
CompName:Pentadecanoic acid, ethyl ester $$ ethyl pentadecanoate $$ n-Pentadecanoic acid ethyl e

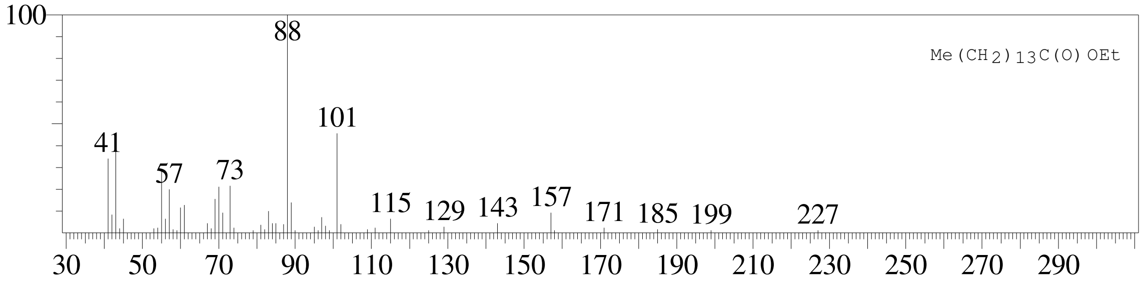

<< Target >>
Line#:23   R.Time:26.467(Scan#:5273) MassPeaks:43
RawMode:Averaged 26.463-26.471(5272-5274) BasePeak:57.05(27750)
BG Mode:Calc. from Peak

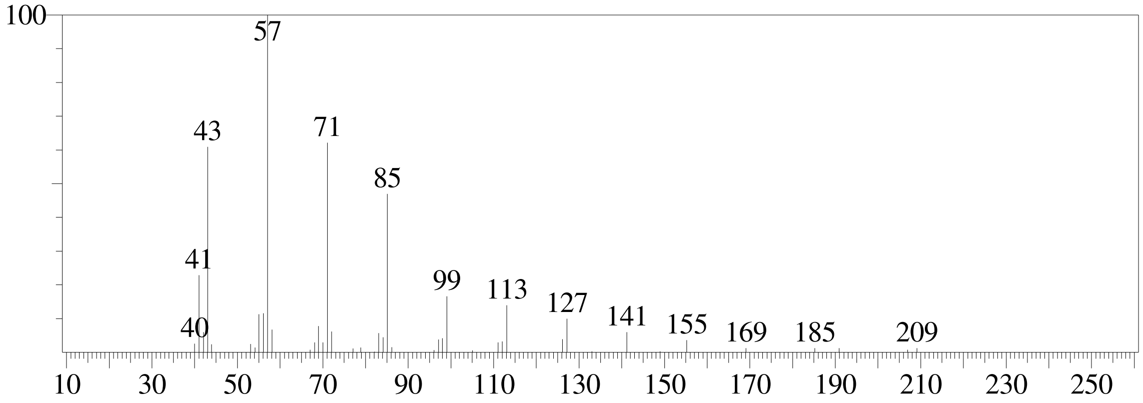

Hit#:1 Entry:902 Library:FFNSC1.3.lib
SI:94 Formula:C18 H38 CAS:593-45-3 MolWeight:254 RetIndex:1800
CompName:Octadecane <n->

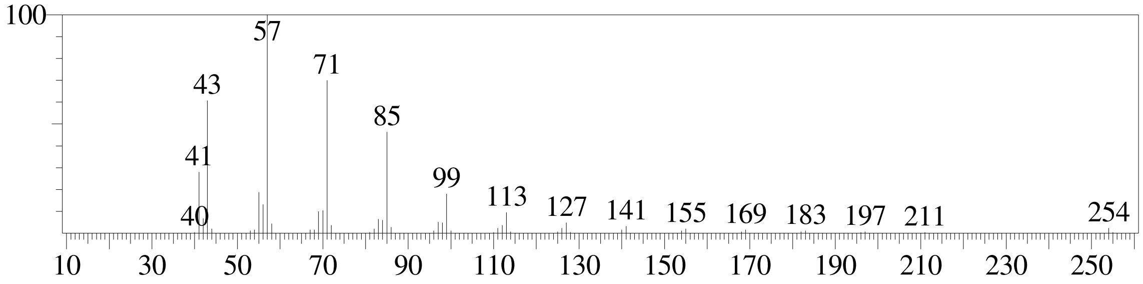

Hit#:2 Entry:895 Library:FFNSC1.3.lib
SI:94 Formula:C17 H36 CAS:629-78-7 MolWeight:240 RetIndex:1700
CompName:Heptadecane <n->

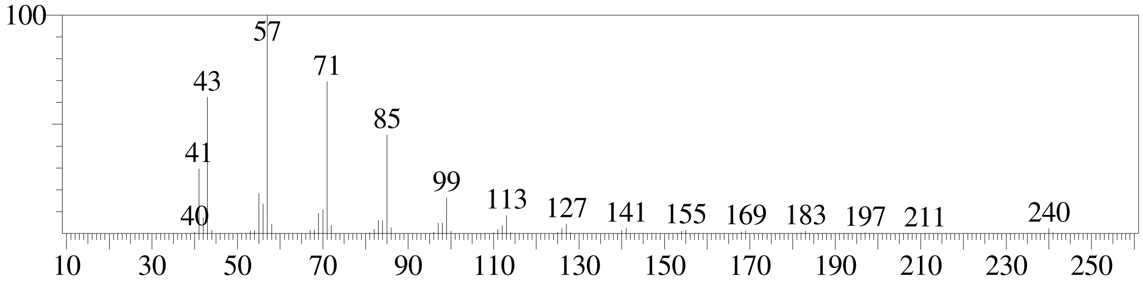

Hit#:3 Entry:72485 Library:NIST11.lib
SI:94 Formula:C17H36 CAS:629-78-7 MolWeight:240 RetIndex:1711
CompName:Heptadecane $$ n-Heptadecane $$ Normal-heptadecane $$

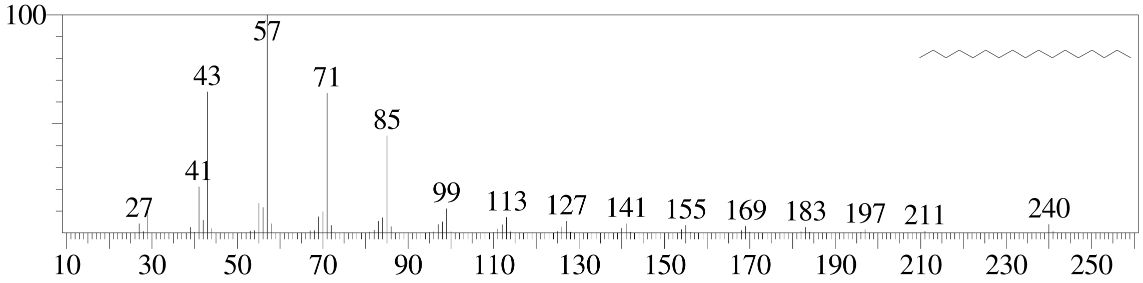

<< Target >>
Line#:24   R.Time:26.792(Scan#:5351) MassPeaks:49
RawMode:Averaged 26.788-26.796(5350-5352) BasePeak:43.00(8601)
BG Mode:Calc. from Peak

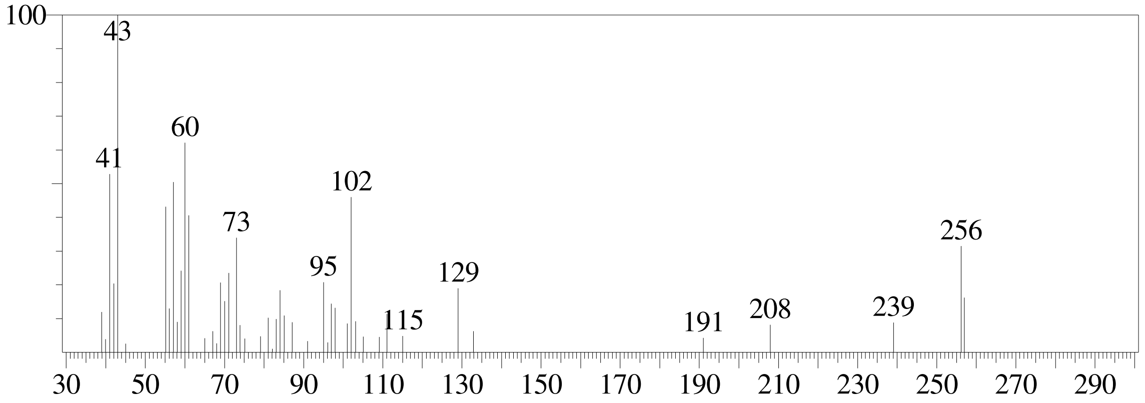

Hit#:1 Entry:599 Library:FFNSC1.3.lib
SI:87 Formula:C19 H38 O2 CAS:142-91-6 MolWeight:298 RetIndex:2023
CompName:Hexadecanoate <isopropyl->

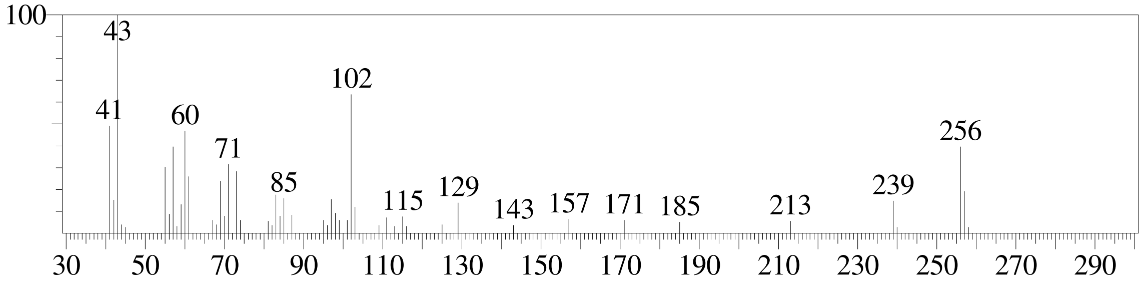

Hit#:2 Entry:117135 Library:NIST11.lib
SI:87 Formula:C19H38O2 CAS:142-91-6 MolWeight:298 RetIndex:2013
CompName:Isopropyl palmitate $$ Hexadecanoic acid, 1-methylethyl ester $$ Palmitic acid, isoprop

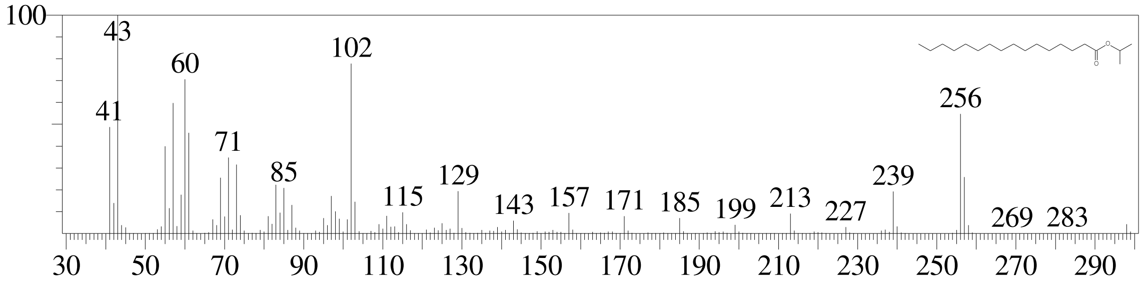

Hit#:3 Entry:209926 Library:WILEY7.LIB
SI:87 Formula:C19 H38 O2 CAS:142-91-6 MolWeight:298 RetIndex:0
CompName:Hexadecanoic acid, 1-methylethyl ester (CAS) Isopal $$ ISOPROPYL ESTER OF PAL

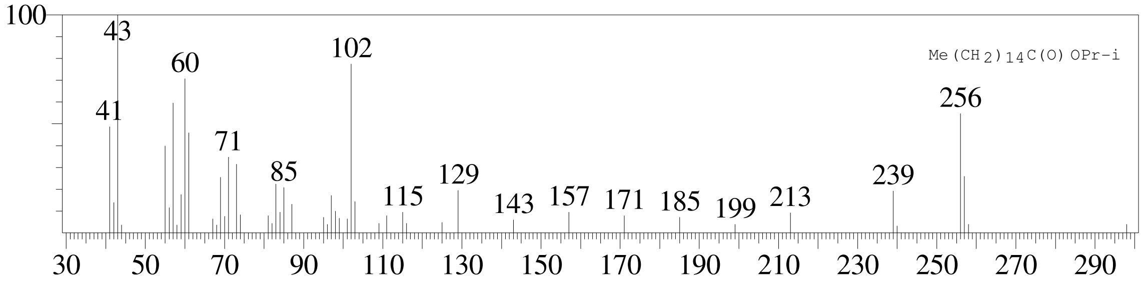

<< Target >>
Line#:25 R.Time:28.754(Scan#:5822) MassPeaks:84
RawMode:Averaged 28.750-28.758(5821-5823) BasePeak:88.00(77220)
BG Mode:Calc. from Peak

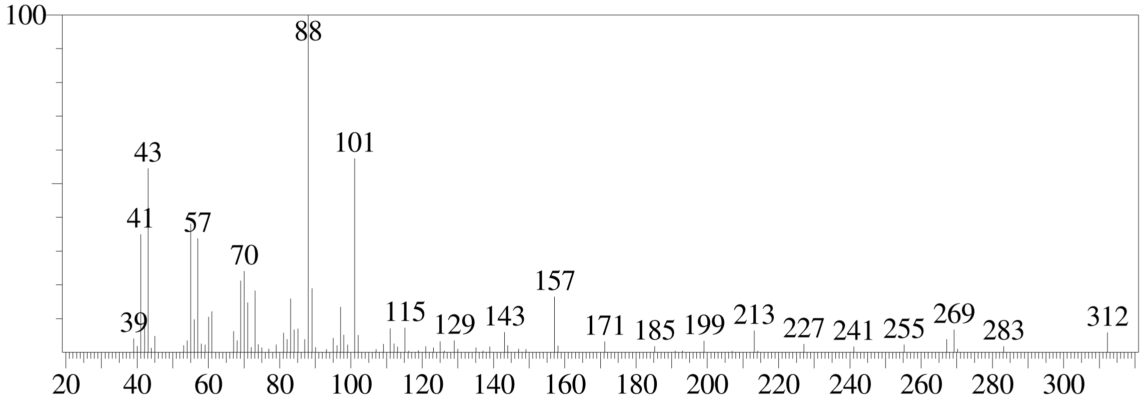

Hit#:1 Entry:26809 Library:NIST11s.lib
SI:94 Formula:C20H40O2 CAS:111-61-5 MolWeight:312 RetIndex:2177
CompName:Octadecanoic acid, ethyl ester $$ Stearic acid, ethyl ester $$ Ethyl n-octadecanoate $$ E

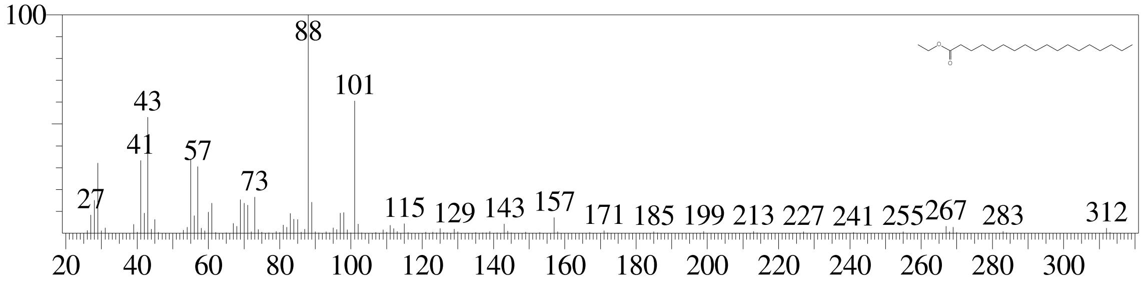

Hit#:2 Entry:223381 Library:WILEY7.LIB
SI:94 Formula:C20 H40 O2 CAS:111-61-5 MolWeight:312 RetIndex:0
CompName:Octadecanoic acid, ethyl ester (CAS) Ethyl stearate $$ ETHYL ESTER OF OCTADEC

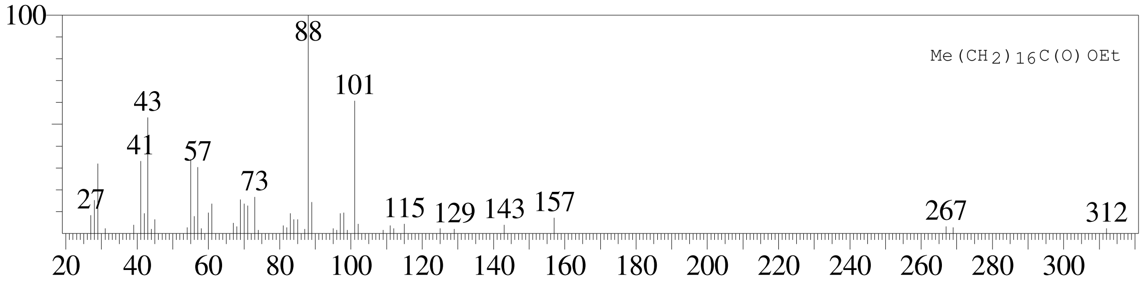

Hit#:3 Entry:223387 Library:WILEY7.LIB
SI:92 Formula:C20 H40 O2 CAS:111-61-5 MolWeight:312 RetIndex:0
CompName:Octadecanoic acid, ethyl ester (CAS) Ethyl stearate $$ ETHYL ESTER OF OCTADEC

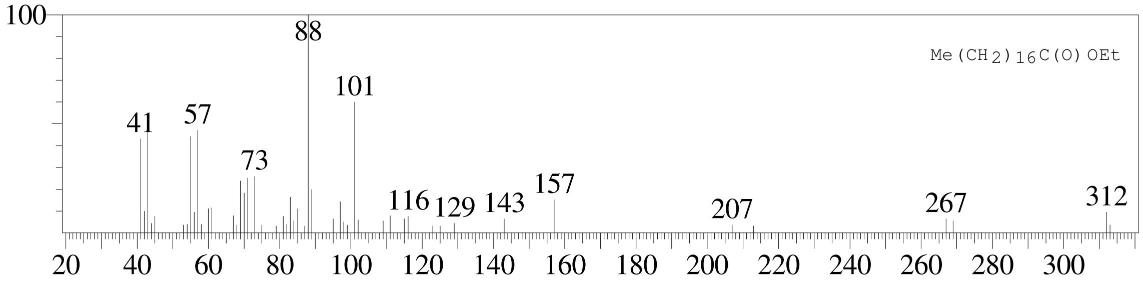

<< Target >>
Line#:26   R.Time:31.975(Scan#:6595) MassPeaks:36
RawMode:Averaged 31.971-31.979(6594-6596) BasePeak:57.10(10181)
BG Mode:Calc. from Peak

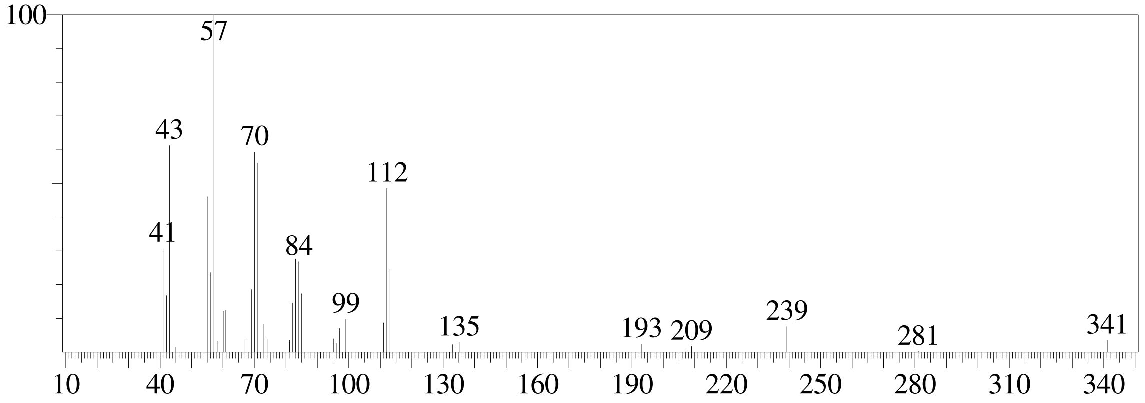

Hit#:1 Entry:223405 Library:WILEY7.LIB
SI:85 Formula:C20 H40 O2 CAS:0-00-0 MolWeight:312 RetIndex:0
CompName:LAURIC ACID, N-OCTYL ESTER $$ LAURINSAEURE, OCTYLESTER $$

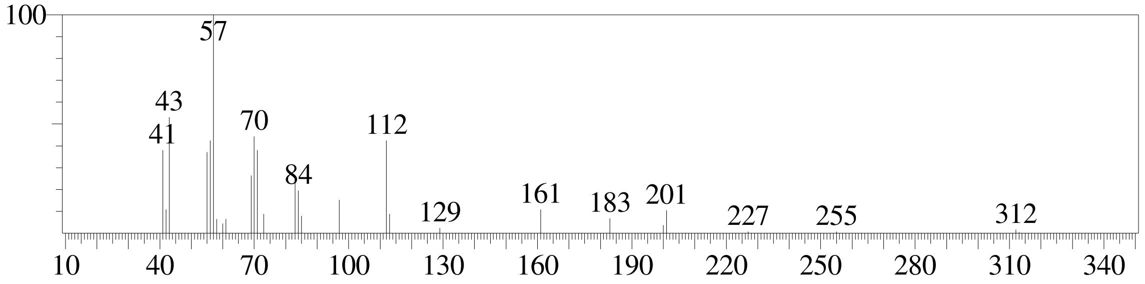

Hit#:2 Entry:148435 Library:WILEY7.LIB
SI:85 Formula:C16 H34 O CAS:629-82-3 MolWeight:242 RetIndex:0
CompName:Octane, 1,1'-oxybis- (CAS) n-Octyl ether $$ Antar $$ Octyl ether $$ Dioctyl ether $$ C

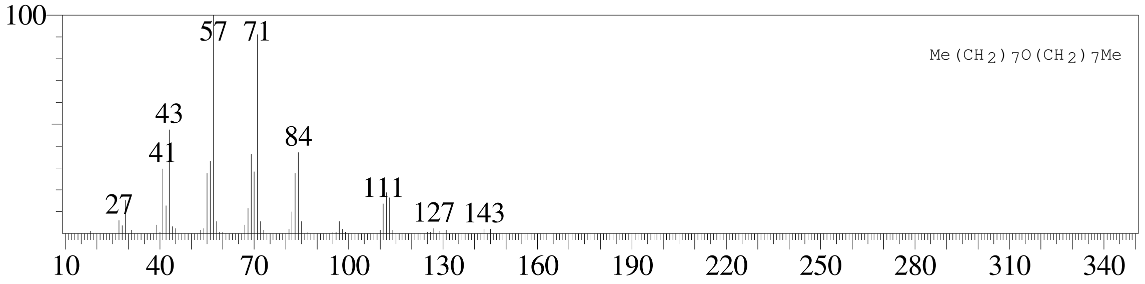

Hit#:3 Entry:236963 Library:WILEY7.LIB
SI:84 Formula:C19 H36 O4 CAS:0-00-0 MolWeight:328 RetIndex:0
CompName:DI(2-ETHYLHEXYL) MALONATE $$

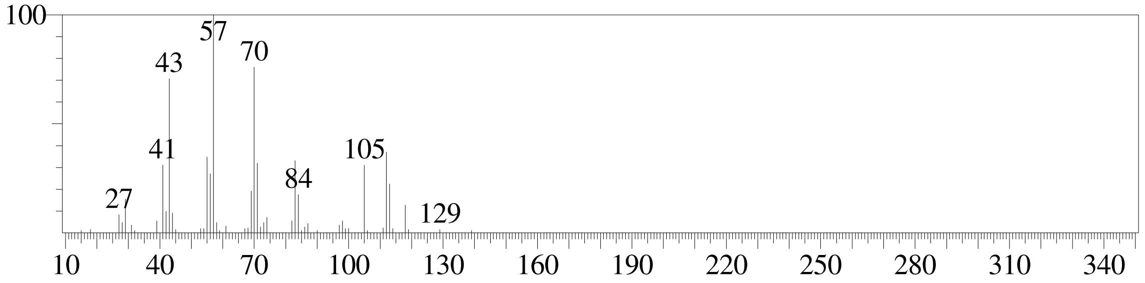

<< Target >>
Line#:27 R.Time:32.313(Scan#:6676) MassPeaks:50
RawMode:Averaged 32.308-32.317(6675-6677) BasePeak:149.00(36074)
BG Mode:Calc. from Peak

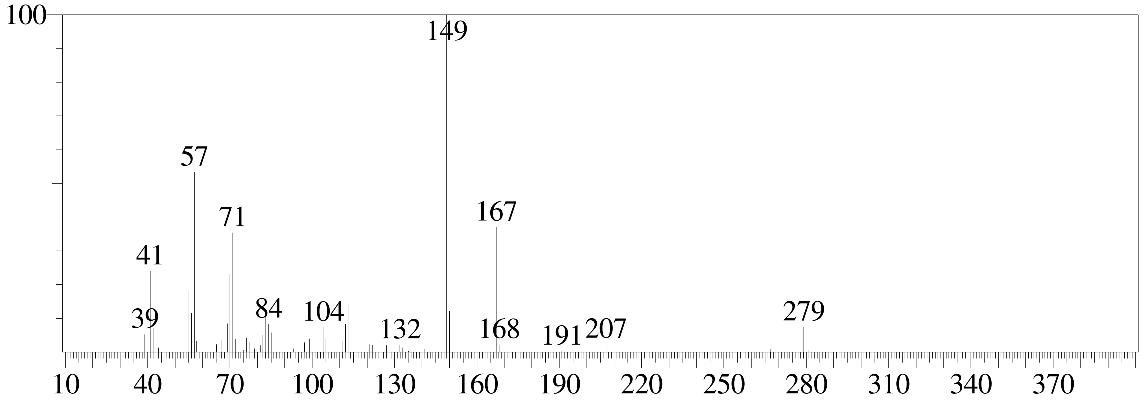

Hit#:1 Entry:29516 Library:NIST11s.lib
SI:92 Formula:C24H38O4 CAS:117-84-0 MolWeight:390 RetIndex:2832
CompName:Di-n-octyl phthalate $$ 1,2-Benzenedicarboxylic acid, dioctyl ester $$ Phthalic acid, dic

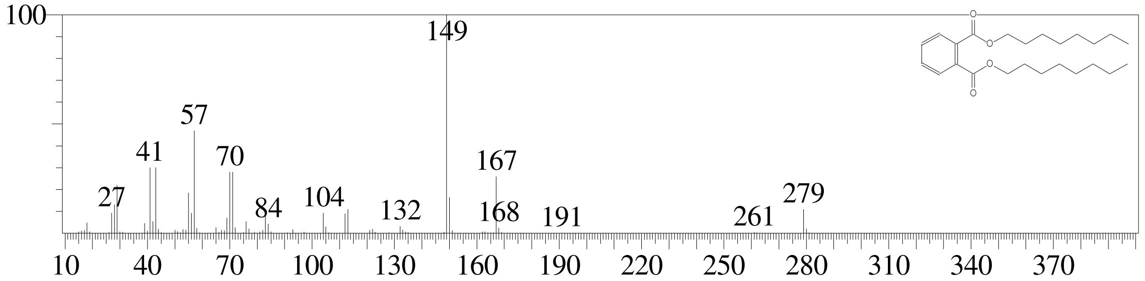

Hit#:2 Entry:279545 Library:WILEY7.LIB
SI:92 Formula:C24 H38 O4 CAS:117-84-0 MolWeight:390 RetIndex:0
CompName:1,2-Benzenedicarboxylic acid, dioctyl ester (CAS) Dioctyl phthalate $$ Dinopol NOP $

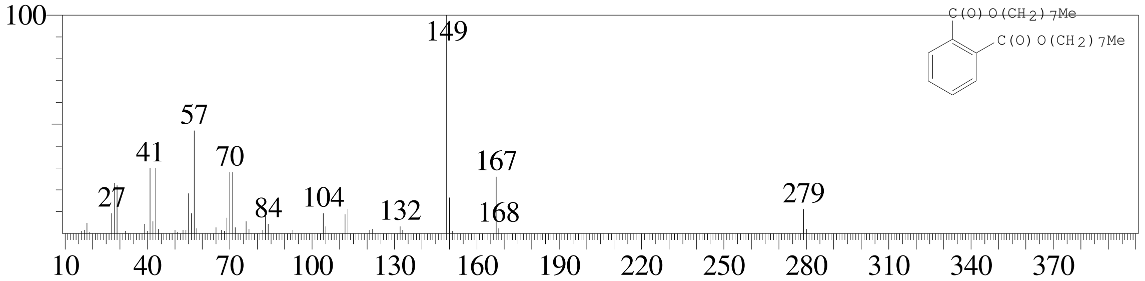

Hit#:3 Entry:279549 Library:WILEY7.LIB
SI:91 Formula:C24 H38 O4 CAS:117-84-0 MolWeight:390 RetIndex:0
CompName:1,2-Benzenedicarboxylic acid, dioctyl ester (CAS) Dioctyl phthalate $$ Dinopol NOP $

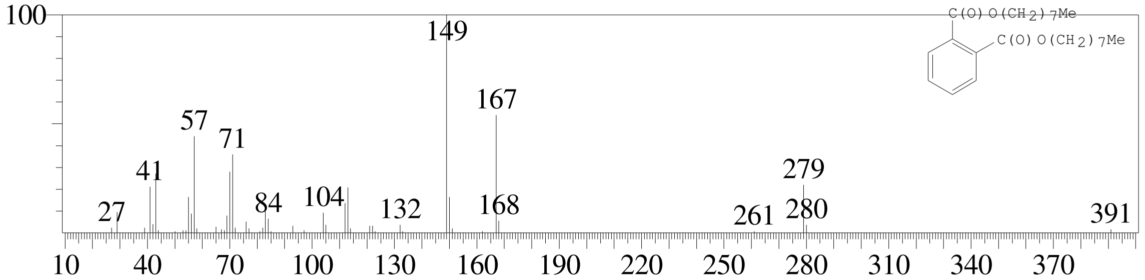

<< Target >>
Line#:28 R.Time:33.888(Scan#:7054) MassPeaks:48
RawMode:Averaged 33.883-33.892(7053-7055) BasePeak:57.10(11684)
BG Mode:Calc. from Peak

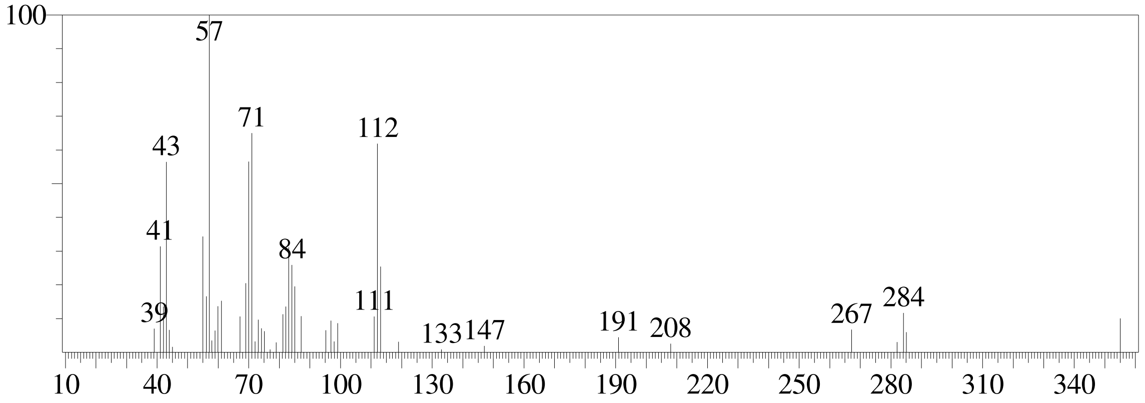

Hit#:1 Entry:148435 Library:WILEY7.LIB
SI:82 Formula:C16 H34 O CAS:629-82-3 MolWeight:242 RetIndex:0
CompName:Octane, 1,1'-oxybis- (CAS) n-Octyl ether $$ Antar $$ Octyl ether $$ Dioctyl ether $$ C

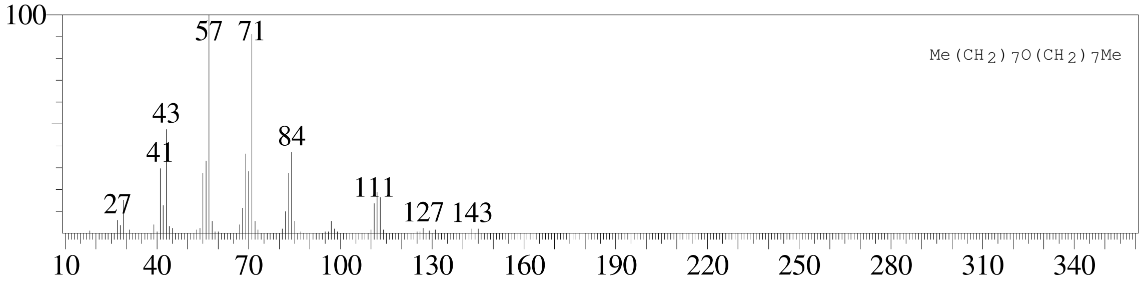

Hit#:2 Entry:150482 Library:WILEY7.LIB
SI:82 Formula:C13 H24 O4 CAS:5398-10-7 MolWeight:244 RetIndex:0
CompName:Propanedioic acid, hexyl-, diethyl ester (CAS) Diethyl hexylmalonate $$ Diethyl 2-hexy

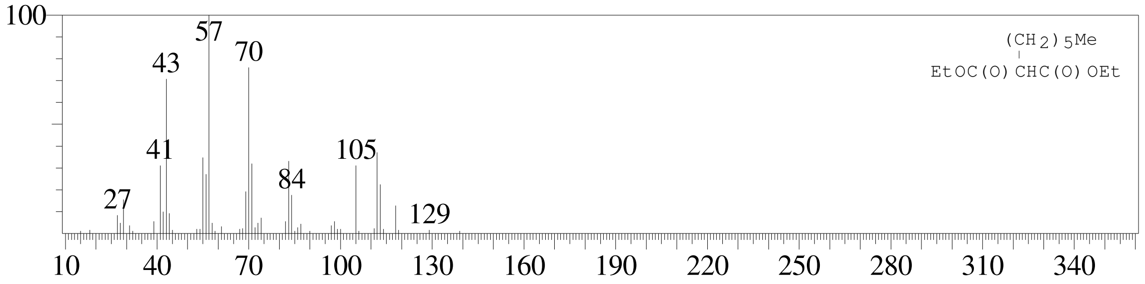

Hit#:3 Entry:236963 Library:WILEY7.LIB
SI:82 Formula:C19 H36 O4 CAS:0-00-0 MolWeight:328 RetIndex:0
CompName:DI(2-ETHYLHEXYL) MALONATE $$

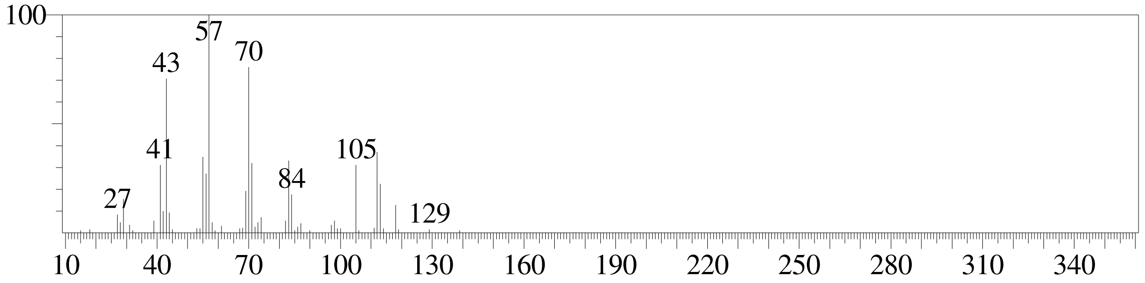

<< Target >>
Line#:29   R.Time:34.896(Scan#:7296) MassPeaks:101
RawMode:Averaged 34.892-34.900(7295-7297) BasePeak:69.05(303383)
BG Mode:Calc. from Peak

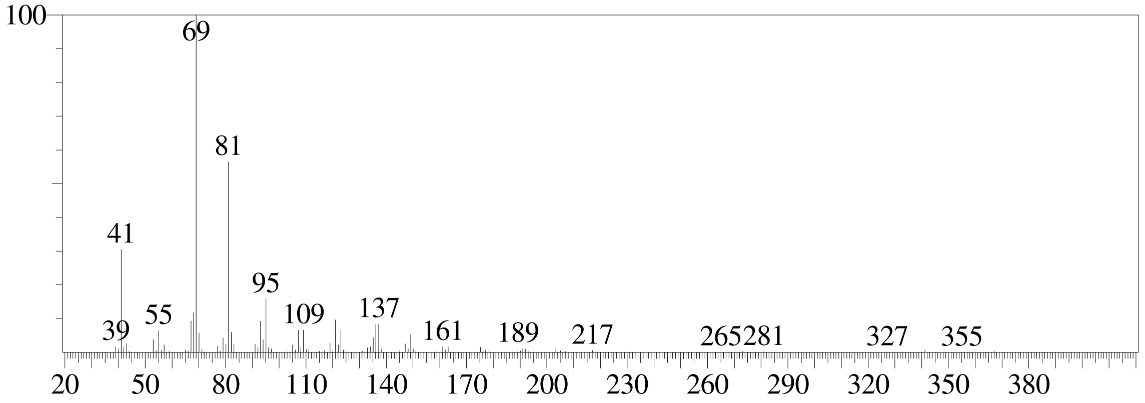

Hit#:1 Entry:29858 Library:NIST11s.lib
SI:97 Formula:C30H50 CAS:111-02-4 MolWeight:410 RetIndex:2914
CompName:Squalene $$ 2,6,10,14,18,22-Tetracosahexaene, 2,6,10,15,19,23-hexamethyl-, (all-E)- $

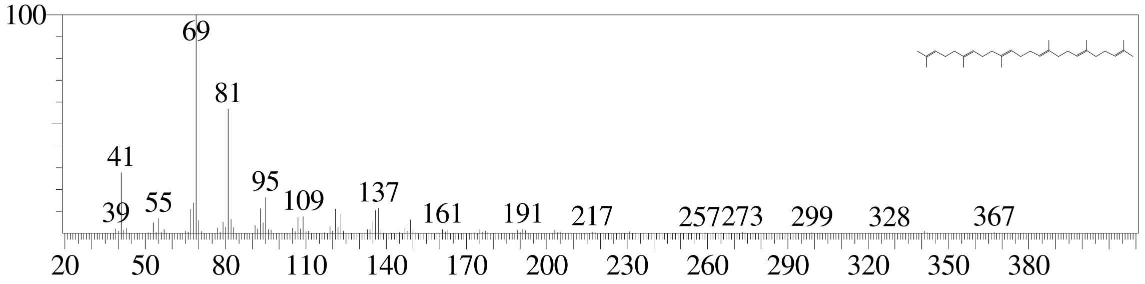

Hit#:2 Entry:186078 Library:NIST11.lib
SI:97 Formula:C30H50 CAS:111-02-4 MolWeight:410 RetIndex:2914
CompName:Squalene $$ 2,6,10,14,18,22-Tetracosahexaene, 2,6,10,15,19,23-hexamethyl-, (all-E)- $

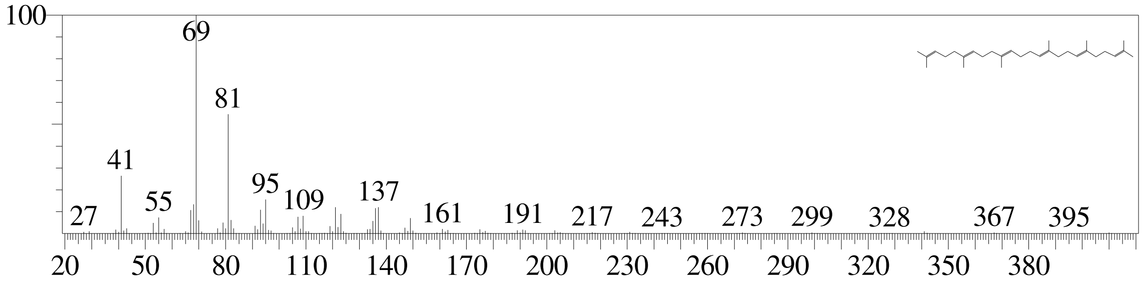

Hit#:3 Entry:289266 Library:WILEY7.LIB
SI:95 Formula:C30 H50 CAS:7683-64-9 MolWeight:410 RetIndex:0
CompName:2,6,10,14,18,22-Tetracosahexaene, 2,6,10,15,19,23-hexamethyl- (CAS) Squalene $$ Sk

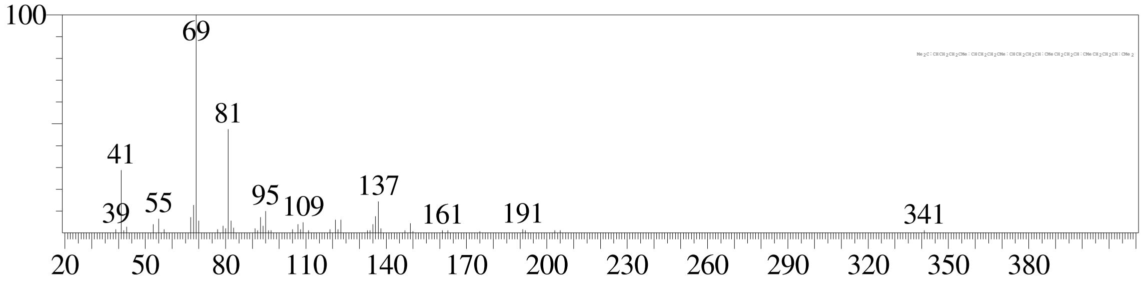

<< Target >>
Line#:30 R.Time:37.671(Scan#:7962) MassPeaks:87
RawMode:Averaged 37.667-37.675(7961-7963) BasePeak:43.05(7605)
BG Mode:Calc. from Peak

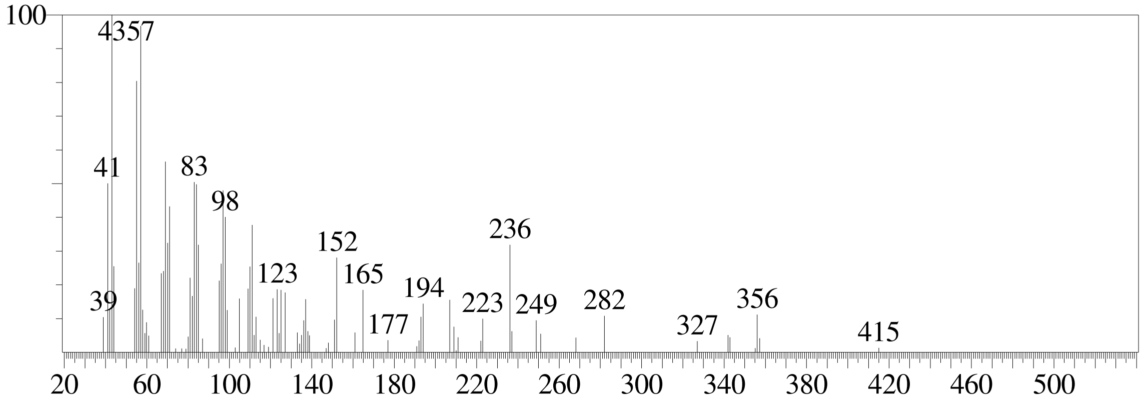

Hit#:1 Entry:317942 Library:WILEY7.LIB
SI:83 Formula:C34 H66 O2 CAS:22393-84-6 MolWeight:507 RetIndex:0
CompName:9-Hexadecenoic acid, octadecyl ester, (Z)- (CAS) OCTADECANYL 9-CIS-HEXADEC

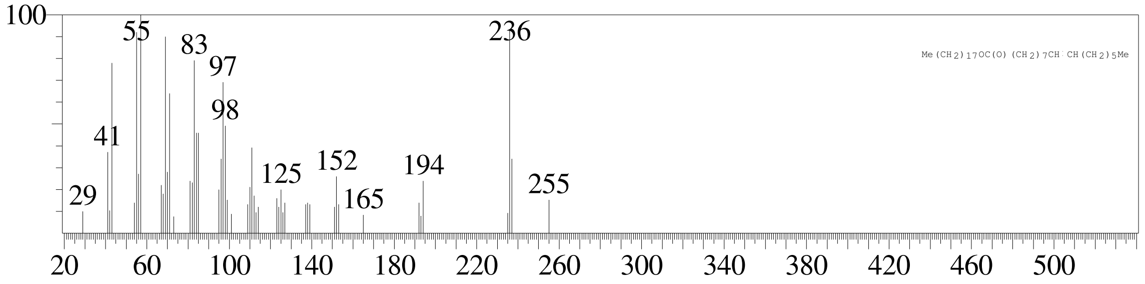

Hit#:2 Entry:205349 Library:NIST11.lib
SI:83 Formula:C34H66O2 CAS:22393-84-6 MolWeight:506 RetIndex:3576
CompName:9-Hexadecenoic acid, octadecyl ester, (Z)- $$ Octadecyl (9Z)-9-hexadecenoate # $$

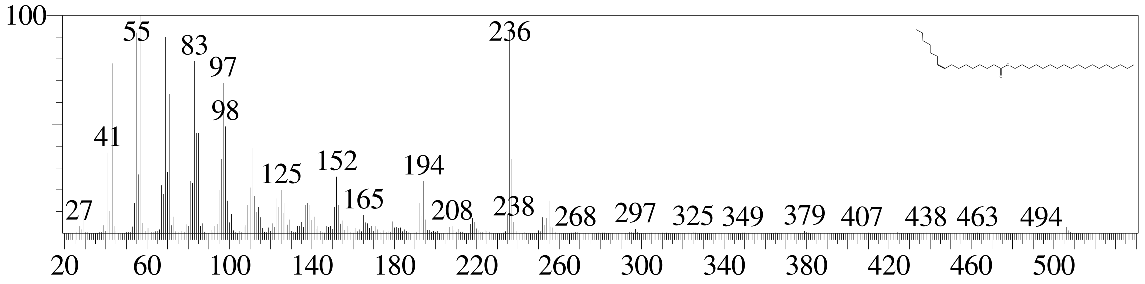

Hit#:3 Entry:207480 Library:NIST11.lib
SI:82 Formula:C36H70O2 CAS:22522-34-5 MolWeight:534 RetIndex:3775
CompName:9-Hexadecenoic acid, eicosyl ester, (Z)- $$ Icosyl (9Z)-9-hexadecenoate # $$

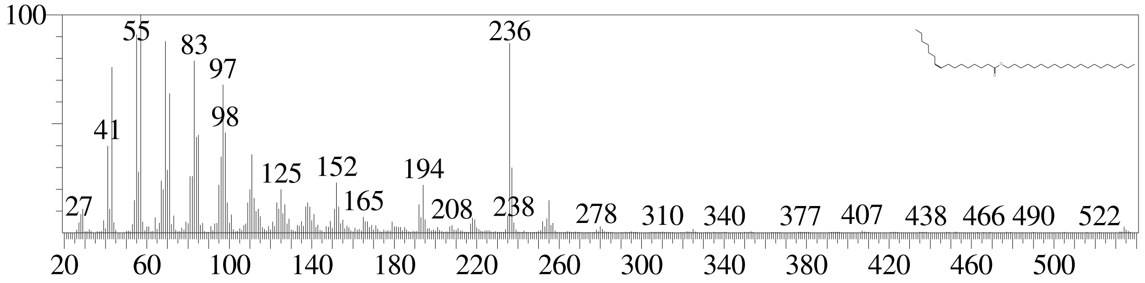

<< Target >>
Line#:31 R.Time:4.521(Scan#:6) MassPeaks:26
RawMode:Averaged 4.517-4.525(5-7) BasePeak:56.05(56917)
BG Mode:Calc. from Peak

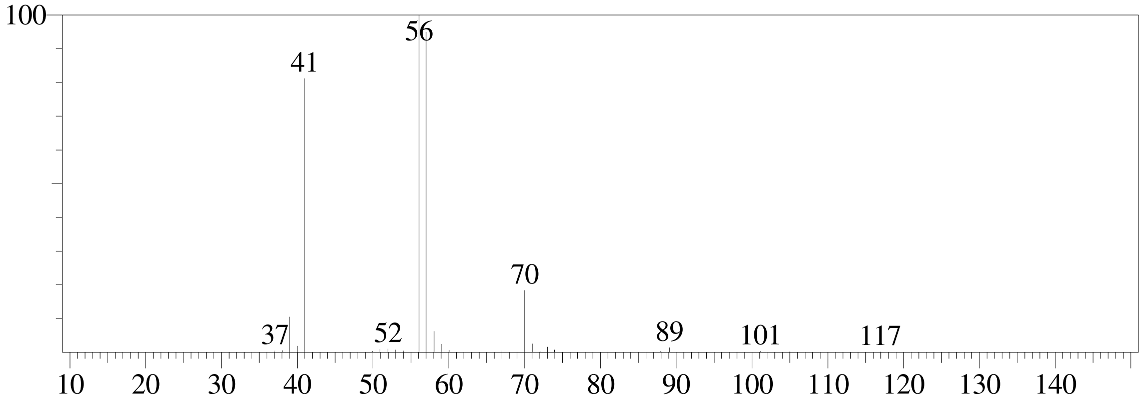

Hit#:1 Entry:4147 Library:WILEY7.LIB
SI:89 Formula:C5 H12 O CAS:123-51-3 MolWeight:88 RetIndex:0
CompName:1-Butanol, 3-methyl- (impure) (CAS) 3-Methyl-1-butanol $$ Isopentanol $$ 3-Methylbu

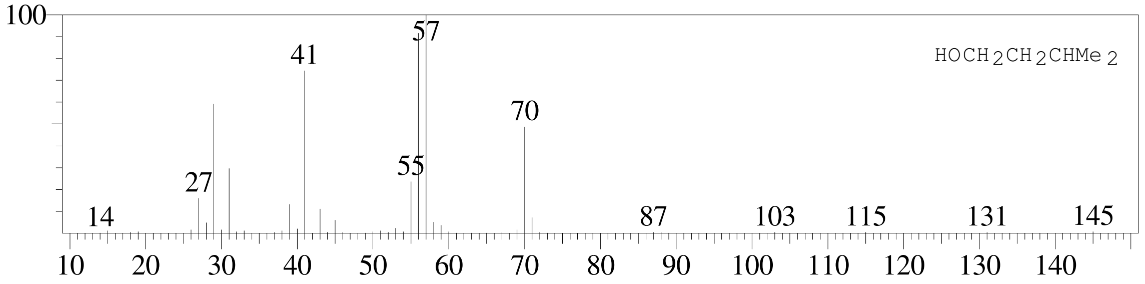

Hit#:2 Entry:4124 Library:WILEY7.LIB
SI:89 Formula:C5 H12 O CAS:137-32-6 MolWeight:88 RetIndex:0
CompName:1-Butanol, 2-methyl- (CAS) 2-Methyl-1-butanol $$ sec-Butylcarbinol $$ 2-Methyl-n-bu

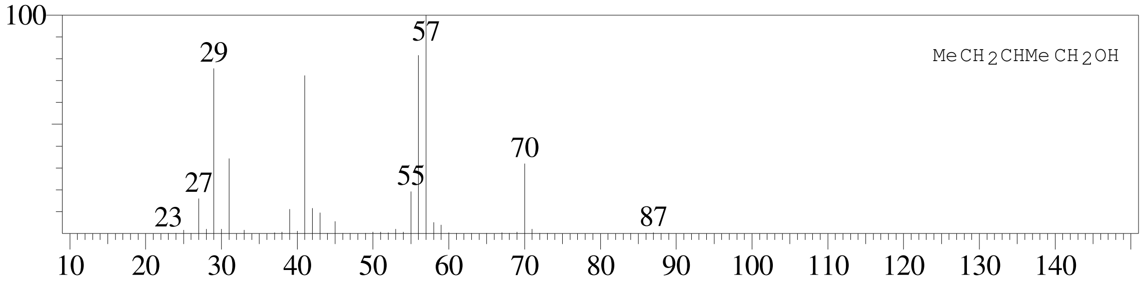

Hit#:3 Entry:4125 Library:WILEY7.LIB
SI:88 Formula:C5 H12 O CAS:137-32-6 MolWeight:88 RetIndex:0
CompName:1-Butanol, 2-methyl- (CAS) 2-Methyl-1-butanol $$ sec-Butylcarbinol $$ 2-Methyl-n-bu

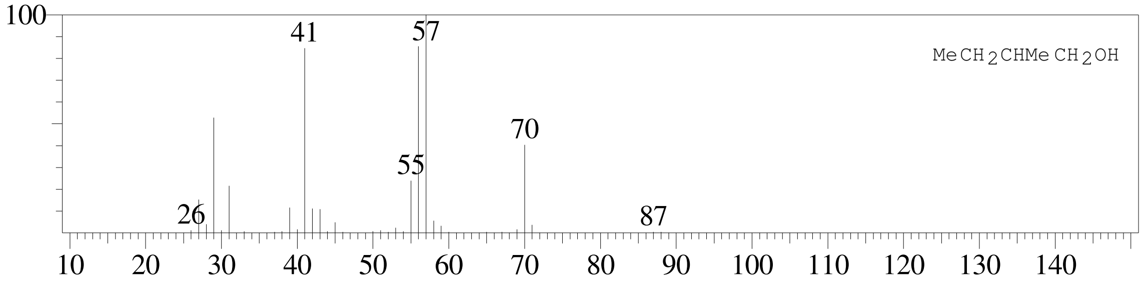

<< Target >>
Line#:32 R.Time:21.842(Scan#:4163) MassPeaks:14
RawMode:Averaged 21.800-21.904(4153-4178) BasePeak:105.00(9504)
BG Mode:Averaged 21.762-21.762(4144-4144)

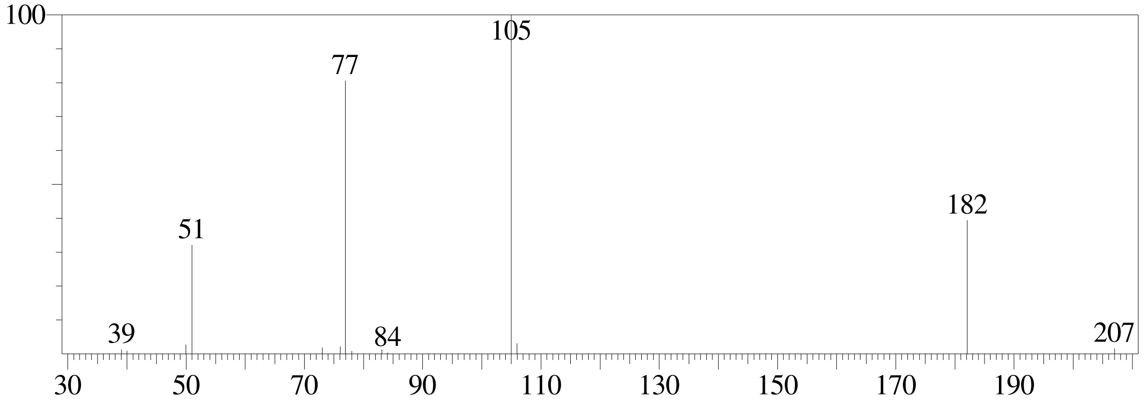

Hit#:1 Entry:74574 Library:WILEY7.LIB
SI:91 Formula:C13 H10 O CAS:119-61-9 MolWeight:182 RetIndex:0
CompName:Methanone, diphenyl- (CAS) Benzophenone $$ Diphenylmethanone $$ Phenyl ketone $$

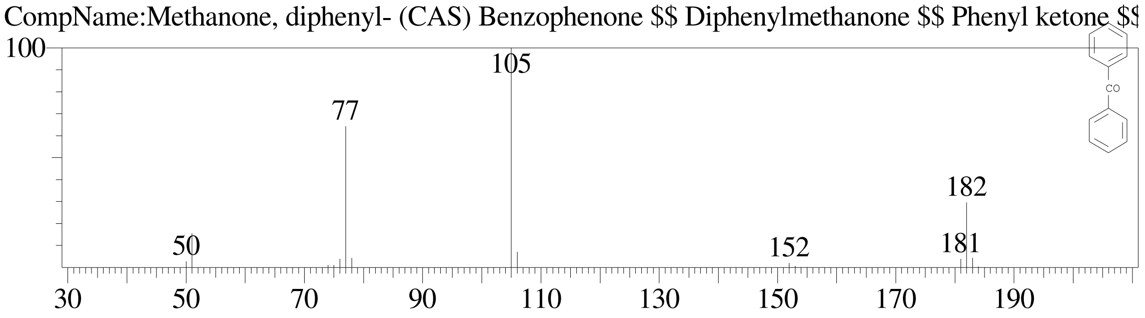

Hit#:2 Entry:74569 Library:WILEY7.LIB
SI:89 Formula:C13 H10 O CAS:119-61-9 MolWeight:182 RetIndex:0
CompName:Methanone, diphenyl- (CAS) Benzophenone $$ Diphenylmethanone $$ Phenyl ketone $$

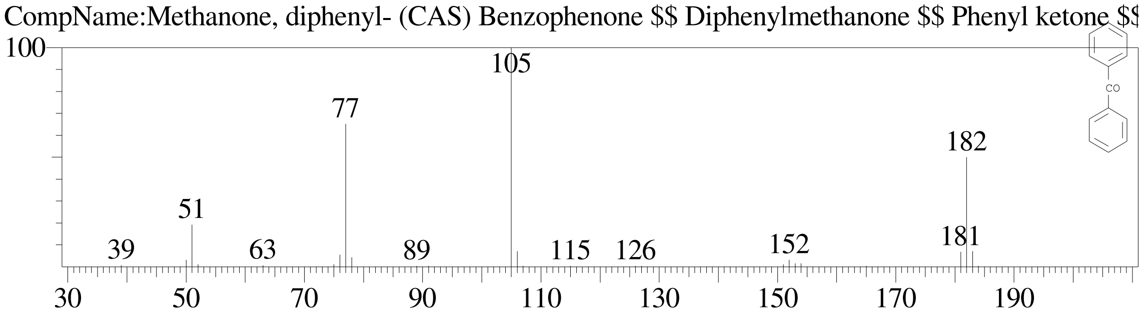

Hit#:3 Entry:304 Library:FFNSC1.3.lib
SI:89 Formula:C13 H10 O CAS:119-61-9 MolWeight:182 RetIndex:1627
CompName:Benzophenone

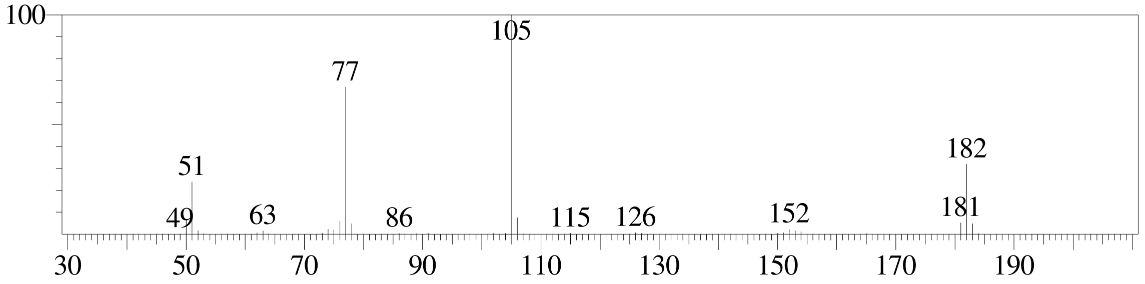

Supplement: S2 Supporting information — (PDF) [file pone.0212451.s002.pdf]
